# Shaping up for structural glycomics: a predictive protocol for oligosaccharide conformational analysis applied to *N*-linked glycans^☆^

**DOI:** 10.1016/j.carres.2013.10.011

**Published:** 2014-01-13

**Authors:** Benedict M. Sattelle, Andrew Almond

**Affiliations:** Faculty of Life Sciences, The University of Manchester, Manchester Institute of Biotechnology, 131 Princess Street, Manchester M1 7DN, UK

**Keywords:** Conformation analysis, Glycome, Microsecond timescale, Kinetics, Molecular dynamics, Puckering

## Abstract

The human glycome comprises a vast untapped repository of 3D-structural information that holds the key to glycan recognition and a new era of rationally designed mimetic chemical probes, drugs, and biomaterials. Toward routine prediction of oligosaccharide conformational populations and exchange rates at thermodynamic equilibrium, we apply hardware-accelerated aqueous molecular dynamics to model μs motions in *N*-glycans that underpin inflammation and immunity. In 10 μs simulations, conformational equilibria of mannosyl cores, sialyl Lewis (sLe) antennae, and constituent sub-sequences agreed with prior refinements (X-ray and NMR). Glycosidic linkage and pyranose ring flexing were affected by branching, linkage position, and secondary structure, implicating sequence dependent motions in glycomic functional diversity. Linkage and ring conformational transitions that have eluded precise quantification by experiment and conventional (ns) simulations were predicted to occur on μs timescales. All rings populated non-chair shapes and the stacked galactose and fucose pyranoses of sLe^a^ and sLe^x^ were rigidified, suggesting an exploitable 3D-signature of cell adhesion protein binding. Analyses of sLe^x^ dynamics over 25 μs revealed that only 10 μs were sufficient to explore all aqueous conformers. This simulation protocol, which yields conformational ensembles that are independent of initial 3D-structure, is proposed as a route to understanding oligosaccharide recognition and structure–activity relationships, toward development of carbohydrate-based novel chemical entities.

## Highlights

• Aqueous 10 μs simulations of *N*-linked mannosyl cores and sialyl Lewis (sLe) antennae are validated.
• Sequence dependent glycosidic linkage and pyranose ring μs motions are implicated in bioactivity.
• Stacked pyranoses in sLe^a^ and sLe^x^ are predicted to be atypically rigid on μs timescales.
• In a 25 μs simulation of sLe^x^, all known conformers were sampled within the initial 10 μs of dynamics.
• Unbiased 10 μs simulations are proposed as a route to systematic and accurate glycomic 3D-analysis.

## Graphical abstract

**Figure:**
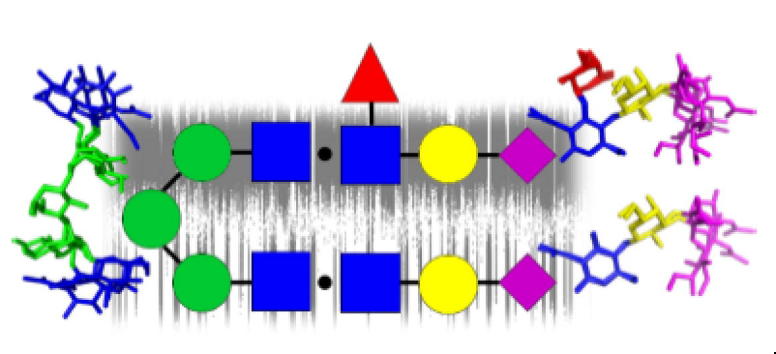

## 1 Introduction

**Figure 1:**
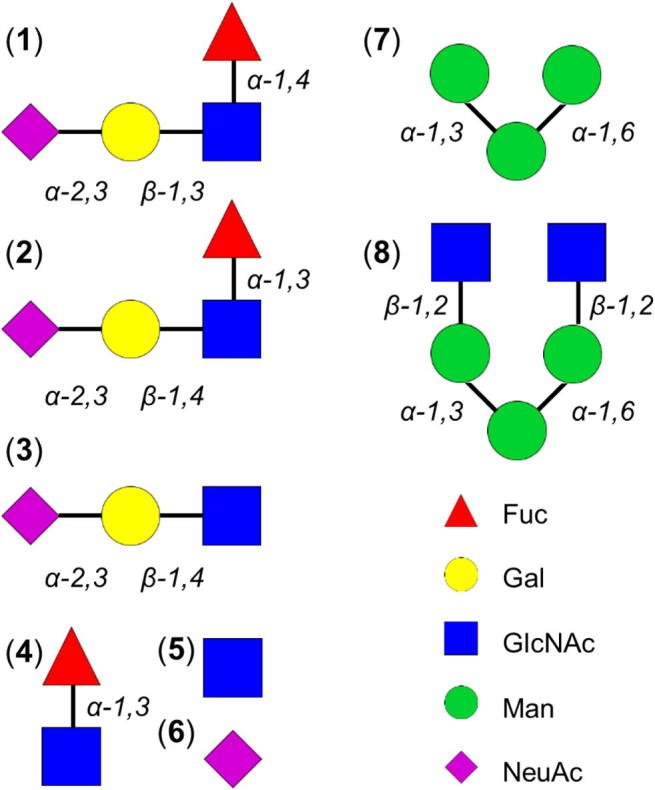
Model *N*-glycans and monosaccharides. Explicit solvent 10 μs simulations are performed for sialyl Lewis^a^**1**, sialyl Lewis^x^**2**, constituent tri-, di-, and monosaccharides **3**–**6**, mannotriose **7**, and a bianntennary *N*-linked core pentasaccharide **8** (for comparison, 25 μs are performed for **2**). Symbols follow the Consortium for Functional Glycomics notation. α-l-Fucose (Fuc), β-d-galactose (Gal), *N*-acetyl-β-d-glucosamine (GlcNAc), α-d-mannose (Man), and *N*-acetyl-α-d-neuraminic acid (NeuAc).

The *N*-linked glycome regulates protein folding, cell signaling, and development and is implicated in fertility, cancer, inflammation, and infection.^1^, ^2^ Although diverse in sequence, *N*-glycans share a common branched trimannosyl core and are often terminated with fucose and sialic acid (Fig. 1). The 3D-structural data encoded by oligosaccharides are largely undeciphered and represent a vast source of information for use in the design of new biomaterials, chemical probes, and drugs.^3^, ^4^ A case in point is the discovery of zanamivir (the first influenza drug to mimic sialic acid), which was aided by structure-based approaches.^5^ Limited 3D-information has been derived from nuclear magnetic resonance (NMR) spectroscopy and X-ray crystallography of *N*-linked sialyl Lewis antennae (**1**–**2**) and mannosyl cores (**7**–**8**).^6^, ^7^, ^8^, ^9^ However, such studies have not yet elucidated the crucial link between sequence and protein specificity for this subset of the human glycome, rendering the burgeoning outputs from high-throughput functional glycomics technologies increasingly difficult to interpret.^10^, ^11^, ^12^ New approaches for oligosaccharide conformational analysis, and their concomitant atomic-resolution insights, are needed to associate 3D-structure with function and to redress the balance.

Within the *N*-linked glycome, sialyl Lewis antigens (e.g., sLe^x^ **2**) have attracted major interest due to their central role in cell–cell recognition via interactions with selectin proteins. At a 3D-molecular level, the closest interactions between sLe^x^ and selectin active-site amino acids are to galactose and fucose.^9^ Forcibly restricting the inter-ring distance between these monosaccharides by chemical methods has led to increasingly potent in vitro selectin inhibitors, highlighting their importance.^13^ These two pyranose rings stack together, forming a compact secondary-structure in water and when protein bound,^14^ and they have been assumed to be rigid chair-like puckers. Recent computational work has questioned the presumption that glycomic pyranoses are inflexible.^15^, ^16^ Furthermore, transiently occupied non-chair pyranose shapes may be the basis for selectivity in protein–oligosaccharide interactions and can be specifically targeted by ring-locked chemical analogs to increase biological activity.^17^ This suggests that a similar approach could be applied to discover *N*-glycan conformational mimetics. Specifically, new 3D-insights into the molecular basis of sialyl Lewis antigen ring flexing, ring stacking, and solvation are needed to drive development of novel hydrophobic analogs for targeting inflammation and cancer.^8^

The branched *N*-linked mannosyl core exhibits flexible glycosidic bonds and conformational exchange at these linkages is an important determinant of protein binding. For example, the core trimannose unit **7** has been hypothesized to exchange between multiple conformers in water, many of which are also present when bound to mannose binding lectin from the innate immune system.^6^, ^7^ However, at the α(1→3) and α(1→6) linkage torsions, disparate conclusions with respect to the number of conformers and their geometric orientations have resulted from numerous NMR studies.^6^ Experiments have only been able to restrict the conformational exchange rates at these linkages to approximate time windows spanning three orders of magnitude. Furthermore, while the transient existence of non-chair puckers has been proposed for mannose in water,^16^ no studies have investigated whether similar ring flexing is present in the *N*-linked mannosyl core. Accurate linkage and ring conformational data for this sequence are of fundamental importance because of their presence in all *N*-glycans. Conversely, inaccuracies erroneously position pendant antennae in 3D-models, which are the basis for understanding multivalent associations with proteins. Therefore, establishing aqueous conformational preferences and exchange rates in the mannosyl core is essential to derive relationships between shape and biological activity in the wider *N*-linked glycome.

Inherent oligosaccharide flexibility hinders conformational analyses of aqueous *N*-linked oligosaccharides using current experimental approaches alone. In particular, crystal growth is impeded by molecular motions, and when possible, X-ray crystallography captures only static 3D-snapshots. Moreover, discrete and transient shapes, such as individual pyranose puckers, are frequently refined from NMR data to average geometries (which may have no true physical meaning^18^). Detailed modeling (including explicit water) could be used to improve interpretation of experiments. However, exchange between structurally-important conformations may not occur in conventional ns timescale simulations. Artificial enhanced sampling techniques allow exploration of longer timescale 3D-transitions, but here, freedom from sensitivity to the initial conformation is achieved at the expense of accurate kinetic predictions.^19^ Recent μs simulations, exploiting technological advances in hardware, have transformed biological understanding in the proteome (e.g., for control of ion channel conductance^20^, ^21^) and similar studies of free monosaccharides and glycosaminoglycans have hypothesized ring puckering to be a μs exchange phenomenon in the glycome.^15^, ^16^, ^22^, ^23^, ^24^ In these studies, pyranose puckering equilibria were also found to be inextricably dependent on sequence; transient pucker lifetimes and the rates of puckering exchange were significantly biased by stereochemistry and substituents. While secondary-structure and chain branching in oligosaccharides can be expected to impact conformation and thus be sources of functional diversity, the extent to which puckering impacts shape and associated biological activity in the *N*-linked glycome remains unknown.

Here, using model *N*-linked mannosyl cores, sialyl Lewis antennae, constituent sub-sequences and monosaccharides, and with the aid of hardware acceleration, we aim to validate a protocol that can accurately and routinely generate equilibrium conformation ensembles for *N*-glycans in water. We generate hypotheses for oligosaccharide conformational transition rates that have been inaccessible to direct experimental observation and conventional simulations (ns and enhanced sampling). The resultant unbiased equilibrium ensembles are used to investigate the effects of sequence on glycosidic linkage and pyranose ring conformational exchange kinetics in oligosaccharides that are central to inflammation and immunity. Toward an improved understanding of carbohydrate recognition, the simulations are used to test the hypothesis that pyranose ring puckering represents a basis for functional diversity in the *N*-glycome.

## 2 Results and discussion

### 2.1 Microsecond conformational exchange in branched oligosaccharides

The 10 μs simulations of **1**–**4**, **7**, and **8** (25 μs for **2**) successfully predict linkage conformers that correlate well with prior interpretations of solution NMR and X-ray data (Table 1), while simultaneously providing new insight into exchange between these states. Specifically, simulations of the α(2→3) and β(1→2) linkages from **1**–**3** and **8**, and also the α(1→3) and α(1→6) linkages from **7** and **8** (which are predicted to exist in diffuse free energy basins), extensively sample the (multiple) conformers refined from previous experiments. Where X-ray crystallography data are available, the consensus experimental linkage observations correlate with simulated conformers that are the most highly populated. Therefore, aqueous molecular dynamics simulations of at least 10 μs duration, comprising the most abundant monosaccharides and glycosidic linkages from the human glycome,^25^ are in good conformational agreement with key experimental data.

**Table 1:** Glycosidic linkage conformers for model *N*-linked oligosaccharides

Torsion	Molecule (s)	SIM (ns) (*ϕ*, *ψ*)°	SIM (μs) (*ϕ*, *ψ*)°	NMR (*ϕ*, *ψ*)°	X-ray^a^ (*ϕ*, *ψ*)°
***α(1**–**3)***					
Man-Man^b^	**7**, **8**	(70,−100)	(70,−100) (160,−80)	(80,−100) (60,180)	(72,−121)
Fuc-GlcNAc^c^	**2**	(−70,−100)	(−70,−100) (−150,−145)	(−72,−96)	(−72,−99)
Fuc-GlcNAc	**4**	(−70,−110)	(−75,−105) (−145,−140) (−80,75)	—	—

***α(1**–**4)***					
Fuc-GlcNAc^d^	**1**	(−70,135)	(−70,135)	(−77,142)	

***α(1**–**6)***					
Man-Man^b^	**7**, **8**	(70,−160)	(70,−160)	(64,180) (64,60)	(67,179) (60,94)

***α(2**–**3)***					
NeuAc-Gal^c^	**1**, **2**, **3**	(60,−120)	(60,−120) (−50,−140)	(60,−120) (−60,−120) (20,170)	(69,−125)

***β(1**–**2)***					
GlcNAc-Man^e^^(1→3)^	**8**	(−90,−70)	(−90,−70) (60,−90)	(−92,−83)	(−80,−98) (58,−87)
GlcNAc-Man ^(1→6)^	**8**	(−90,−70)	(−90,−70)	—	—

***β(1**–**3)***					
Gal-GlcNAc^d^	**1**	(−70,−110)	(−70,−110)	(−70,−99)	(−74,−132)

***β(1-4)***					
Gal-GlcNAc^c^	**2**	(−70,130)	(−70,130)	(−74,138)	(−71,132)
Gal-GlcNAc	**3**	(−70,130)	(−70,130) (−70,−50) (−150,−100)	—	—

Torsion states derived from the simulations (SIM) are from μs-simulations (μs) and the initial 100 ns of dynamics (ns) for **1**–**4**, **7**, and **8**. All μs data are calculated from 10 μs simulations except **2** (25 μs). Prior refinements from solution NMR^6^, ^30^, ^55^, ^56^ and X-ray^29^ (in various environments) are tabulated.

a Ref. 29.

b Ref. 6.

c Ref. 32.

d Ref. 57.

e Ref. 55. Experimental data for **1** is for Lewis^a^ (lacking sialic acid).^57^

Simulated ring puckering of constituent monosaccharides converges to equilibrium within 3–5 μs (Supplementary data), as seen previously for free pyranoses and in glycosaminoglycans.^15^, ^16^, ^22^, ^23^, ^24^ Ring puckering equilibria in the simulations firmly favor chairs, as expected (^4^*C*_1_ in d-pyranoses, ^1^*C*_4_ in l-Fuc and ^2^*C*_5_ in NeuAc). Chair inversions or transitions between chair and intermediate (boat and skew-boat) shapes (on μs-timescales) are present in 19 of the 23 rings modeled. Notably, the Gal and Fuc rings from the simulations of **1** and **2** are the only pyranoses that do not populate intermediate puckers. Comparing 55 chemically equivalent calculated and experimental pyranose ring ^1^H–^1^H three-bond vicinal spin-couplings (^3^*J*_HH_) from GlcNAc, Gal, NeuAc, and Man, 51 are within ±2.0 Hz (Supplementary data), in good agreement and consistent with expectations.^15^, ^16^, ^22^, ^23^, ^24^ The remaining four relate to ^*3*^*J*_4,5_ or ^*3*^*J*_5,6_ in NeuAc. Here, the predicted values are consistently smaller than their experimental equivalents (deviating by at most 2.4 Hz), suggesting a slight overestimation of non-chair puckers. This finding concurs with previous μs simulations of flexible idopyranoses,^22^, ^24^ where computed ring ^3^*J*_HH_ values were in trend agreement with experiment but absolute values deviated by a similar magnitude and force-field errors were suspected. Our results here also suggest that small force-field modifications to GLYCAM06^26^ may be required to improve the modeling of ring motions in NeuAc.

### 2.2 Secondary structure and microsecond motions in sialyl Lewis antennae

**Figure 2:**
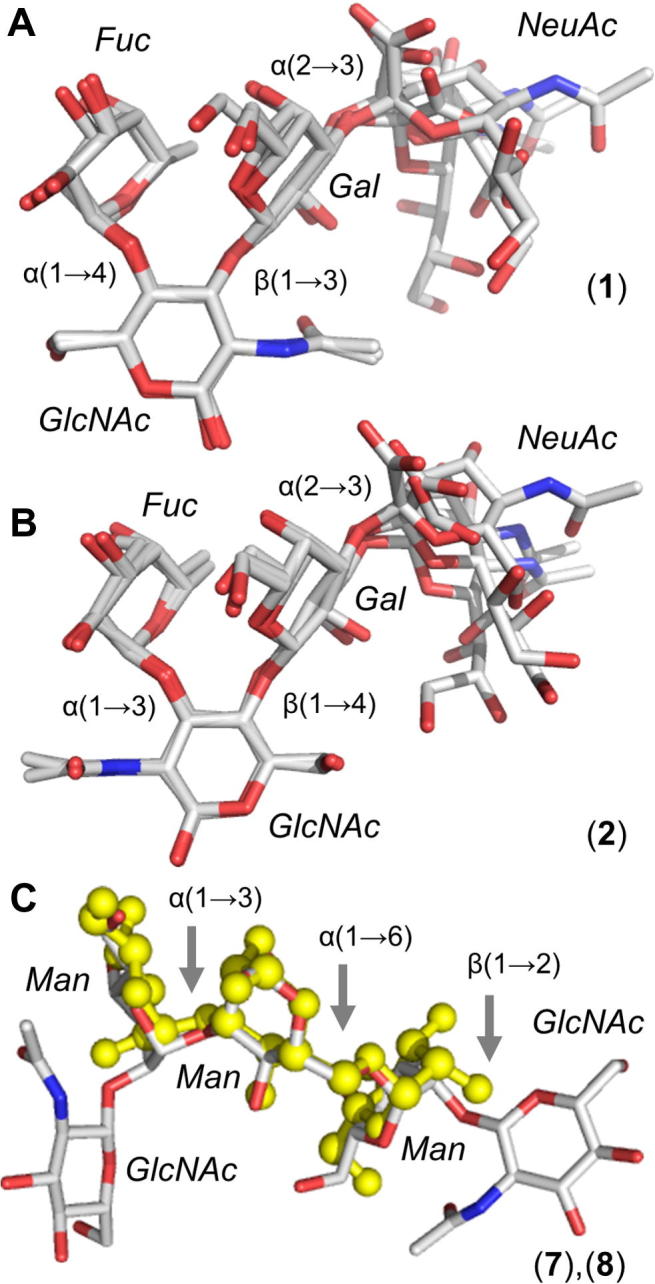
Experimentally consistent major aqueous conformers for model *N*-glycans. (A) sialyl Lewis^a^**1**, (B) sialyl Lewis^x^**2**, and (C) overlaid mannosyl core sequences **7** (ball and stick representation) and **8**. Depicted conformers are the most populated 3D-shapes in 10 μs simulations (25 μs for **2**) and are derived via hierarchical 3D-clustering. Hydrogen atoms are hidden for clarity.

The sialyl Lewis antigens have similar overall conformations in the simulations (Fig. 2, panels A and B). Centroids of the Gal and Fuc rings are on average 4.6 (±0.2) and 4.5 (±0.2) Å apart in **1** and **2**, respectively, consistent with 800 MHz aqueous NMR performed here (Supplementary data). This stems from the Gal and Fuc rings stacking to form tight local secondary structure, as concluded previously.^14^ Computed order parameters for intra-ring (calibration) and inter-ring (measured) motions fall within a narrow range (≈0.9 in **1** and ≈0.8 in **2**), providing confidence in the Gal:H2 to Fuc:H5 inter-ring distances derived from NMR intensity extrapolations (2–3 Å in **1** and **2**). The μs predictions are identical, confirming that the simulations accurately predict the close stacking interaction in **1** and **2** (Supplementary data).

**Figure 3:**
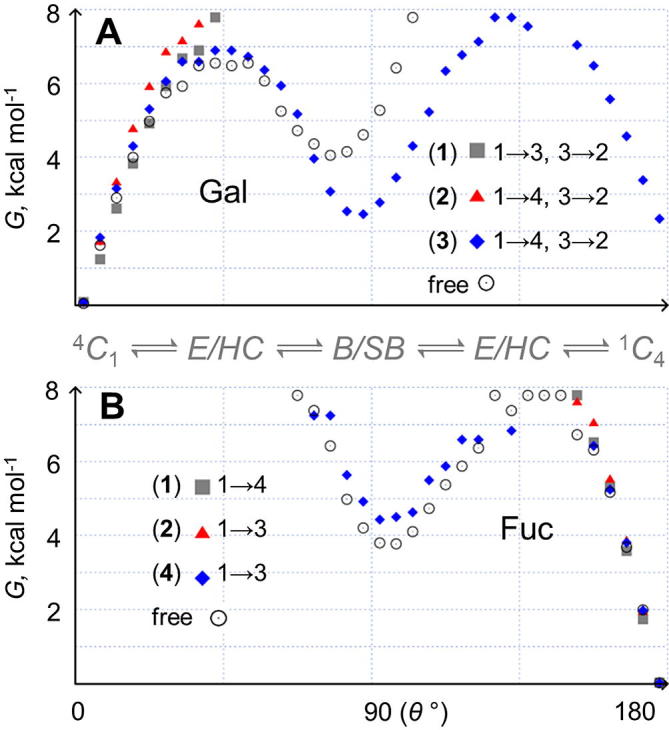
Microsecond pyranose puckering in stacked monosaccharides from Lewis antennae. (A) Gal and (B) Fuc. One dimensional puckering free energy surfaces are derived from 10 μs simulations of **1**–**3** (25 μs for **2**). For chain-linked rings, linkage positions are indicated and unlinked monosaccharides labeled as free (data from Sattelle & Almond, 2012).^16^ Chair (*C*), envelope or half-chair (*E*/*HC*) and boat or skew-boat (*B*/*SB*) conformational regions are noted. Errors are calculated to be ±0.01, ±0.2 and ±0.6 kcal mol^−1^ at Δ*G* values of 5, 7 and >9 kcal mol^−1^, respectively (see Supplementary data).

Coincidentally, in these oligosaccharide simulations Gal populates only chair and chair-like puckers; this is in stark contrast to a prior μs-simulation of the free Gal monosaccharide^16^ and the simulation of the trisaccharide **3**, where ring stacking is absent and frequent μs-timescale chair-chair exchange (ring inversion) via intermediate non-chair puckers is observed (Fig. 3, panel A). Comparison of Fuc puckering in the simulations of **1** and **2** with the disaccharide **4** reveals that the Fuc ring is also a rigidified chair in the tetrasaccharides exhibiting the stacking interaction (Fig. 3, panel B). In the modeled sialyl Lewis antigens, proximity to Gal causes fewer non-chair Fuc puckers to be sampled (cf. **4**) and the most accessible shapes are chair-like envelopes and half-chairs with relatively high free energies (greater than 6 kcal mol^−1^ above the lowest energy chair pucker). In the absence of stacking (i.e., in **4**) the Fuc residue reverts to a μs-puckering equilibrium resembling that of free aqueous Fuc.^16^ Based on these findings it is concluded that stacking of Gal and Fuc in Lewis antennae is responsible for their reduced ring flexibility. Importantly, this atypical rigidity is present in a region that closely interacts with the active site of selectin proteins and thus is potentially exploitable in the development of therapeutics. For instance, chemical analogs that can mimic the predicted rigidified ring characteristics of Gal and Fuc in type 1 and type 2 Lewis antigens could be prioritized.

**Figure 4:**
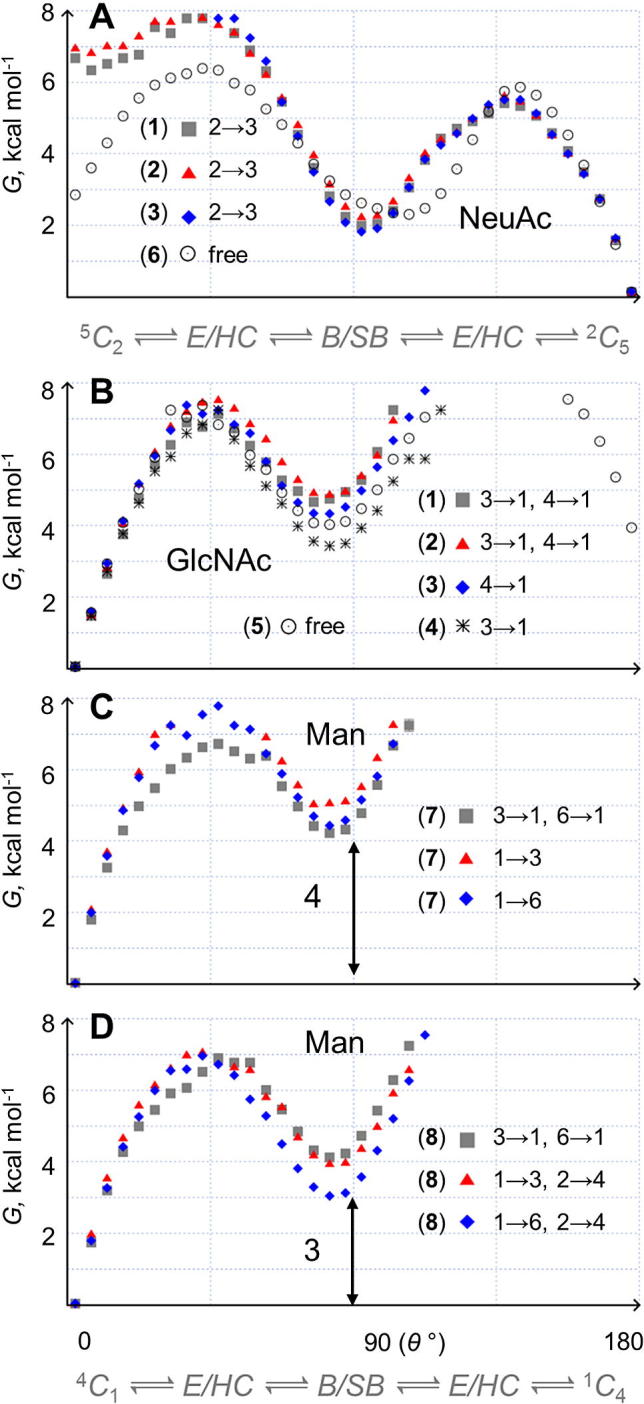
Microsecond pyranose ring puckering in model *N*-glycans and free monosaccharides **1**–**8**. (A) NeuAc, (B) GlcNAc, (C), and (D) Man. One dimensional puckering free energy surfaces are derived and depicted identically to those in Figure 3 (see Fig. 3 caption for details).

In **1** and **2** the GlcNAc monosaccharide is linked to both Gal and Fuc pyranoses and is key to the biologically important stacking interaction. In the μs-simulations of oligosaccharides **1**–**4** and free β-GlcNAc **5**, the GlcNAc pyranose rings are all metastable (^4^*C*_1_) chairs. However, the populations of non-chair puckers differ significantly (Fig. 4, panel B). Only free β-GlcNAc **5** inverts (to ^1^*C*_4_, at a rate of 0.5 μs^−1^), although slightly slower than computed previously for free α-GlcNAc (0.8 μs^−1^),^15^ implying that stereochemistry affects GlcNAc μs-puckering (as for idose).^23^ In the chain-linked GlcNAc rings of **1**–**4** the intermediate puckers are most populated in the di- and trisaccharides **4** and **3**,which both lack the stacking interaction and wherein GlcNAc is connected at either the 3- or 4-position only (Δ*G* ≈ 3–4 kcal mol^−1^, measured relative to the ^4^*C*_1_ conformer). In **1** and **2** GlcNAc is linked at both the 3- and 4-positions and here intermediate puckers are comparatively less stable (Δ*G* ≈ 5 kcal mol^−1^). This suggests that the GlcNAc pyranose attached to stacked Gal and Fuc rings in sialyl Lewis antennae, is a more stable chair compared to when it is connected at only one site in oligosaccharides lacking the stacking interaction. It remains to be determined whether Gal and Fuc stacking rigidifies the GlcNAc ring or if the relatively stable GlcNAc chair pucker in fact maintains the stacked shape of type 1 and type 2 sialyl Lewis antigens.

**Figure 5:**
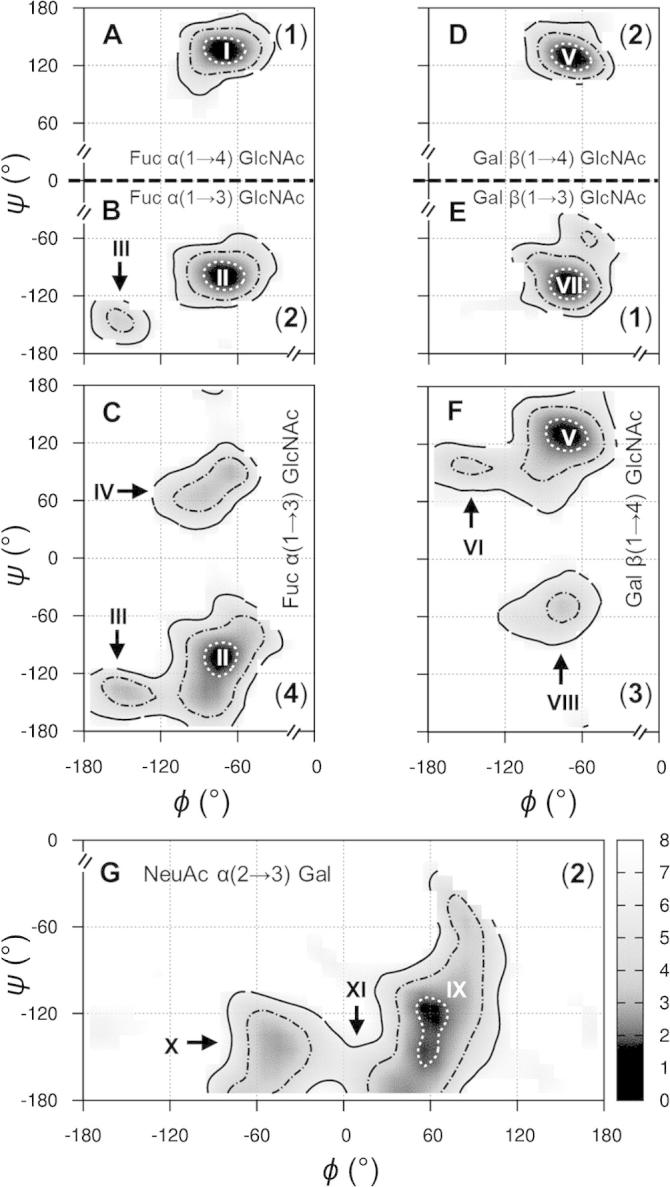
Aqueous glycosidic linkage free energy surfaces in model *N*-glycans **1**–**4**. Surfaces are derived from 10 μs simulations (25 μs for **2**). Free energies of binned conformers, relative to the most populated state, are color scaled in the range 0 (black) to >8 (white) kcal mol^−1^. Contours are depicted at 2, 4, and 6 kcal mol^−1^ using dashed-white, dashed-black, and solid-black lines, respectively. Low energy conformers discussed in the text are indicated with roman numerals and axes are truncated to show only the populated regions of conformation space.

Experimental NMR studies and ns-timescale simulations have attributed the restricted dynamic range of linkage conformations in Lewis antennae to steric crowding and decreased solvent accessibility.^27^, ^28^ At the α(1→3) and β(1→4) linkages in **1** and **2**, compared to their equivalents in the tri- and disaccharides **3** and **4**, reduced flexibility is also present in the μs-simulations (note the additional conformers **IV** and **VIII** in Fig. 5, panels C and F). The effect of solvent is confirmed by the finding that less water is present in the second solvation shell (3.0–5.0 Å) surrounding the α(1→3) and β(1→4) linkages in **2** (cf. **3** and **4**). However, radial distribution functions between glycosidic linkage oxygen and water hydrogen atoms (in **2**, **3** and **4**) suggest that in **2** the α(1→3) and β(1→4) linkage conformers are stabilized by oligosaccharide–water interactions (reduced in **3** and absent in **4**) within the first hydration shell of these glycosidic bonds (1.0–2.5 Å) (Supplementary data). Therefore, specific water–sLe^x^ interactions at the α(1→3) and β(1→4) linkages are proposed to be essential for maintaining the biologically important stacked shape of **2** (and presumably other similar oligosaccharides).

At the termini of **1**–**3** the most populated α(2→3) linkage conformation in the simulations concurs with numerous prior X-ray studies of **1**, **2** and structurally related molecules (state **IX**, Fig. 5, panel G).^29^, ^30^ Conversely, previous NMR refinements have proposed dynamic sub-ns exchange between three α(2→3) linkage states in **2**,^31^, ^32^ all of which are populated in the simulations of **1**–**3** (conformers **IX**, **X**, and **XI**). State **XI** has a low population in the simulations, consistent with previous experimental reports,^32^ and lies halfway between **IX** and **X**. Therefore, it is concluded that **XI** is likely a virtual conformer^18^ (i.e., an artifact of refining multiple conformations to a single state) that is not significantly populated in water. While the predicted α(2→3) linkage dynamics in **1**–**3** substantiate a generally conserved and mobile epitope for sialyl Lewis antennae termini, transition between **IX** and **X** is a μs-timescale exchange in the simulations, rather than the sub-ns equilibrium estimated previously from NMR relaxation measurements.^32^ Furthermore, the α(2→3) linkage motions are notably more alike in **1** and **2** compared to **3**, which has a restricted dynamic range that may be attributable to the absence of the stacking interaction.

The chain-terminating NeuAc rings in the μs-simulations of **1**–**3** are meta-stable (^2^*C*_5_) chairs (Fig. 4, panel A). All have a skew-boat (^6^*S*_2_) as the most populated intermediate, which is highly accessible at ≈1–2 kcal mol^−1^ above the lowest energy chair. Non-chair NeuAc puckers can persist for up to ≈0.8 μs and the major intermediate (^6^*S*_2_) is notably favored in **3** compared to in **1** and **2**, which possess the stacking interaction. Transition to inverted (^5^*C*_2_) chair-like puckers is extremely rare in the terminal NeuAc rings, resulting in a large free energy difference between the two chairs (Δ*G* ≈ 6–7 kcal mol^−1^). In contrast, free NeuAc **6** frequently inverts (22 μs^−1^), with the two chairs being much closer in free energy (Δ*G* ≈ 3 kcal mol^−1^) and the energetic barrier to inversion being lower (ΔΔ*G* ≈ 2 kcal mol^−1^). A further notable difference in the simulation of **6** is that the major intermediate is a boat (^4,O^*B*, with ^4^*S*_2_, ^O^*S*_3_ and ^6^*S*_2_ being the next most populated puckers, as in prior enhanced ns-simulations^33^). Additionally, at ≈2–3 kcal mol^−1^ above the most populated (^2^*C*_5_) chair free energy, intermediate puckers are comparatively disfavored and short-lived in **6** (cf. in **1**–**3**). Therefore, it is concluded that NeuAc μs-puckering in oligosaccharides is dependent on its connectivity and the local chemical environment, which could underlie the numerous and diverse biological roles associated with this monosaccharide. For example, any bias in the pyranose conformational equilibrium will affect the spatial orientation of ring substituents, which in NeuAc are chemical groups whose positioning determines selectin binding.^14^

### 2.3 Microsecond dynamics in the *N*-linked mannosyl core

In the simulations of **7** and **8** the predominant linkage states within the trimannosyl core are extremely similar (overlaid in Fig. 2, panel C), supported by prior NMR studies of related *N*-linked oligosaccharides that concluded no significant effect of terminal GlcNAc substitution on overall shape.^34^ However, the trimannose unit contains very flexible linkages, which differ in the simulations of **7** and **8**. Changes in μs-motions may affect protein recognition of *N*-glycans and have not been amenable to direct observation or precise quantification by experiment, as is the case for the conformations, relative free energies and exchange rates resolved in these simulations.

**Figure 6:**
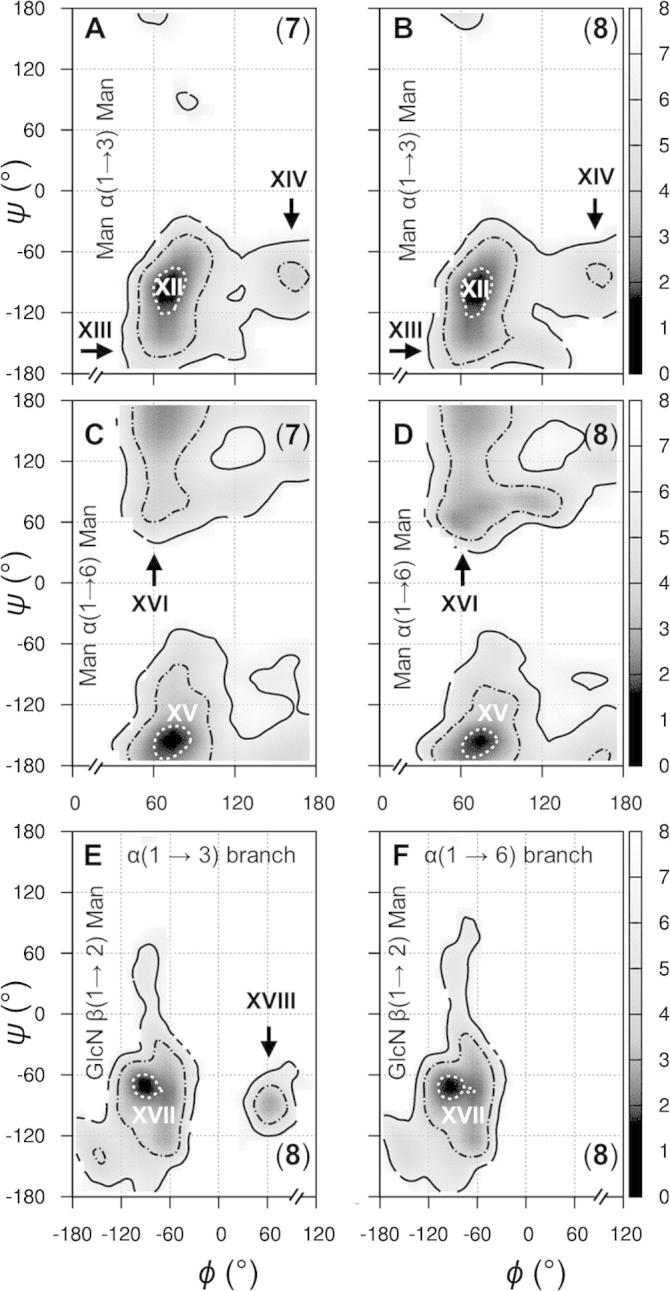
Aqueous glycosidic linkage free energy surfaces in model *N*-glycans **7** and **8**. Calculated surfaces and annotations are derived and depicted identically to those in Figure 5 (see Fig. 5 caption for details).

At the mannosyl core α(1→3) linkages, prior NMR studies of **7** in water (and when bound to mannose binding lectin) have concluded that a broad free energy minimum exists and two predominant states (**XII** and **XIII**) and minor conformer (**XIV**) were refined (Fig. 6, panels A and B).^6^, ^7^ The major states were proposed to be in fast ps to ns exchange and to be approximately equally populated.^6^, ^7^ In the simulations of **7** and **8**, states **XII**-**XIV** are located within diffuse free energy basins. Time series confirm ps to ns exchange at the α(1→3) *ψ* dihedrals in water and the simulations strongly suggest conformer **XII** to be favored, with the two previously refined major states being separated by ≈4 kcal mol^−1^. Exchange between **XII** and **XIV** is a μs-transition in the simulations and notably favored in **7** (cf. **8**).

**Figure 7:**
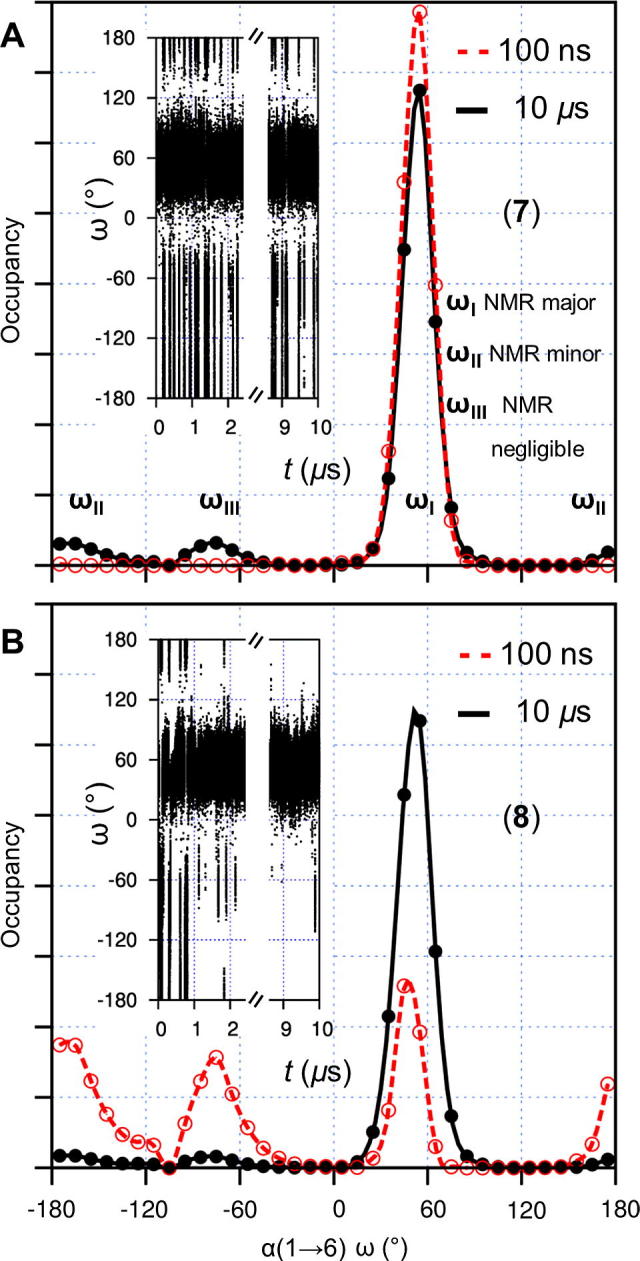
Microsecond motions at the α(1→6) linkage *ω* torsion in the model *N*-glycans **7** (A) and **8** (B). Broken axis simulated torsion time series are inset and plots show torsion occupancies derived from 10 μs simulations (black, closed circles) and from the initial 100 ns of dynamics (red, open circles). Conformers and their relative occupancies refined from prior solution NMR data^6^, ^7^ are indicated with roman numerals.

Considering the α(1→6) linkage *ϕ* and *ψ* torsions, one major state (**XV**) and one minor conformer (**XVI**) have been interpreted in prior NMR studies of **7** (Fig. 6, panels C and D).^6^ The simulations predict a single and extremely broad free energy minimum centered on state **XV** and spanning ≈120° in both *ϕ* and *ψ*. Interestingly, the α(1→6) linkage free energy surfaces of **7** and **8** possess different topologies at the minor conformer **XVI**, which is notably favored and relatively broad in **8** (cf. **7**). Considering the *ω* torsion, one major conformer (***ω*_I_**) and two minor states (***ω*_II_** and ***ω*_III_**) are present in the simulations and their orientations and relative populations concur with previous NMR refinements for **7** (Fig. 7, discrepancy in ns-simulations is notable).^6^ However, in this and another experimental study,^34^ exchange rates between ***ω*_I_** and ***ω*_II_** could not be interpreted more accurately than to within three orders of magnitude. The simulations here reveal the conformational exchange at the α(1→6) *ω* torsion of **7** to occur at ≈40 μs^−1^. In **8**, this exchange is notably slower as a result of terminal GlcNAc substitution (Fig. 7).

A further effect of the local chemical environment is seen at the GlcNAc β(1→2) Man linkages of **8**, present in all *N*-linked glycans. On the basis of 61 X-ray structures, two conformers are possible at this bond (Fig. 6, panels E and F), states **XVII** and **XVIII** (53 and 8 refinements, respectively).^29^ In the simulation of **8**, the β(1→2) linkages are centered on state **XVII**. However, on the α(1→3) branch only, this bond transitions after 9 μs in the simulation to state **XVIII**, which persists for ≈0.5 μs. The predicted relative populations of these conformers accord with the X-ray data, which have not been the case in prior ns-simulations (e.g., **XVIII** has been reported previously at <1% in the disaccharide^30^).

The simulations also suggest differences in puckering equilibria for the constituent GlcNAc and Man pyranoses in **7** and **8** (Fig. 4, panels B–D). In **8**, the α(1→3) branch terminal GlcNAc does not undergo chair–chair exchange during the simulation, while the α(1→6) branch GlcNAc ring inverts twice. All modeled Man ring puckers are meta-stable (^4^*C*_1_) chairs, the inverted (^1^*C*_4_) chair is not sampled in **7** or **8** and the most populated intermediates are the boat and skew-boat puckers *B*_3,O_ and ^2^*S*_O_. These two intermediates have similar free energies relative to the ^4^*C*_1_ chair in **7** and **8** (Δ*G* ≈ 4 kcal mol^−1^) at the branched Man rings (3- and 6-position attached). However, in the α(1→3) and α(1→6) linked Man rings, the *B*_3,O_ and ^2^*S*_O_ puckers are less favored in **7** compared to **8** (ΔΔ*G* ≈ 1 kcal mol^−1^). This implies that terminal GlcNAc substitution renders the preceding α(1→3) and α(1→6) linked Man rings more flexible by lowering the free energy barrier to inter-conversion from the ^4^*C*_1_ chair to intermediate puckers. Therefore, μs-puckering of Man rings in the ubiquitous mannosyl biantennary core sequence from *N*-glycans can be expected to be altered by chain elongation.

### 2.4 Is puckering a basis for functional diversity in the wider glycome?

The five monosaccharides considered here on μs-timescales are the most prevalent human glycomic building blocks. The most abundant is GlcNAc (32%), with Gal (25%), Man (19%), NeuAc (8%), and Fuc (7%) also being common and NeuAc is the predominant chain-terminating ring.^25^ Gaining new insights into μs-puckering in these pyranoses have extensive ramifications for rationalizing and exploiting the plethora of biological processes in which they are known to participate. Pyranose flexing in these fundamental sugars is not inconsistent with high-resolution crystallographic data (⩽2.0 Å); for example, non-chair puckers are present in protein X-ray co-crystals for Fuc,^16^ GlcNAc,^15^ Man,^35^ and NeuAc.^33^

Furthermore, NMR ring vicinal couplings for pyranoses are frequently lower than expected in ideal chair geometries, and single molecule studies have revealed experimental evidence for the presence and biological importance of non-chair puckers.^36^ In the case of flexible 2-*O*-sulfo-iduronic acid from heparin, a small aqueous population (≈10%)^22^ of the ^2^*S*_O_ pucker is responsible for initiating anticoagulation.^37^ Understanding this phenomenon from a structural perspective has resulted in new and safer conformationally-restrained anticoagulants.^17^ Here, populations of non-chair pyranose puckers are predicted to range from ≈1% (GlcNAc, Gal, Man, Fuc) to ≈16% (NeuAc) and to be dependent on ring substituents (as previously)^16^, ^23^ and also the local environment (i.e., ring stacking and linkage attachments). All of these rings participate in molecular recognition and have been found to do so in non-chair puckers. Thus, considered alongside previous experiments and μs glycosaminoglycan studies,^24^ the results herein provide a novel and compelling 3D-structural view of the entire glycome that includes hitherto overlooked μs conformational equilibria, while remaining consistent with experimental data.

Comparing free and chain-linked monosaccharides, calculated ^3^*J*_H,H_ values are within ±1 Hz and consistent with the magnitude of equivalent differences in prior measurements for free and chain-linked pyranoses (e.g., in Gal, GlcNAc, Man, and NeuAc),^6^, ^38^, ^39^, ^40^ yet significantly different ring puckering ensembles are predicted. The puckering found here in branched *N*-glycan sequences extend the hypothesis that ring flexing is inextricably related to function in the glycome.^24^ While pyranose μs-exchange and individual puckers remain difficult to observe by NMR, puckering in the simulations is not inconsistent with experimental data. If true, the elucidation of sequence-dependent puckering in *N*-glycans significantly modifies the conventional view of glycomic monosaccharide building blocks as rigid chairs and the data present new opportunities for understanding protein selectivity in specific glycomic sequences. The implication that ring puckering is modulated by chemical sequence, and that this is a molecular basis for functional diversity in the glycome, should be considered in future efforts to rationalize oligosaccharide biological activity and in structure-based design of conformational analogs.

### 2.5 A protocol for in silico glycomics

Analyses of the dynamics and comparisons with experiments suggest that μs-timescale explicit solvent simulations (using GLYCAM06^26^ and TIP3P^41^) water can reproduce conformational equilibria in biologically-relevant and chemically diverse oligosaccharide linkages and rings (at least within the resolution of current experimental data). This method was also sufficient to approach equilibrium in monosaccharides and linear sulfated glycosaminoglycans containing up to ten pyranose rings.^15^, ^16^, ^22^, ^23^, ^24^ Reanalysis of the initial 100 ns from each simulation reveals that all modeled glycosidic linkages remain within the free energy basin of the starting conformer, a finding that is at odds with prior experiments (Table 1). In particular, NMR^6^, ^32^ and X-ray studies^29^ have proposed multiple states at the considered β(1→2) and α(2→3) linkages, for which ns-simulations appear insufficient to traverse the aqueous free energy barriers.

To assess the benefit of even longer simulations for oligosaccharides, analyses of **2** are compared over both 10 and 25 μs. Average linkage torsions, the number of predicted linkage states and average distances are found to be identical in 10 and 25 μs trajectories. Furthermore, in the 25 μs simulation of **2** averaging beyond 10 μs results in identical puckering ensembles. Therefore, oligosaccharide linkage and ring motions are well sampled in 10 μs simulations and hence are close to equilibrium. Consequently, on this timescale the predicted conformational ensembles are independent of the starting conformation and initial geometries for simulations can be prepared by non-experts in the knowledge that aqueous linkage and puckering states present in experimental data will be sampled. This finding paves the way for routine in silico conformational analysis in a sizable fraction of the human glycome.

Advancements in massively parallel computing, including new algorithms^42^ and hardware,^20^ promise sustained progress in carbohydrate modeling. Our findings (e.g., Table 1) are consistent with previous reports, which have concluded that extending carbohydrate simulations to μs timescales is essential to explain experimental observations.^43^ While for large biopolymers longer timescales and alternate coarse-grained methods^24^ may be needed, this is not the case for 95% of mammalian *O*- and *N*-glycans, which contain less than eight monosaccharides.^25^ For these molecules, and in particular where aqueous 3D-shapes are unknown a priori, oligosaccharide simulations that approach thermodynamic equilibrium on μs timescales can be used to routinely generate 3D-ensembles that are independent of the initial structure.^24^ Furthermore, any deviation of calculated properties from experiment can be attributed to force-fields rather than incomplete sampling, permitting iterative improvement of theoretical methods.

Overcoming the sampling limitations of ns modeling and gaining μs kinetic insights (which are difficult to obtain from NMR spectra and biased in artificially enhanced simulations^19^) are essential to advance beyond reconfirming initial 3D-structures and to derive new experimentally-consistent structural hypotheses from oligosaccharide conformational ensembles at thermodynamic equilibrium. The proposed hardware-accelerated simulation protocol currently requires extensive computational resources and the management of large data sets. For example, approximately two months run-time (on a single graphics processing unit) and 100 gigabytes of storage are needed for the 10 μs explicit solvent simulation of **1**. However, these issues are currently not prohibitive (exemplified by recent ms timescale protein simulations^20^) and they will be ameliorated by technological innovation, promising routine μs conformational insights for aqueous oligosaccharides in the near future.

## 3 Conclusions

In summary, 10 μs simulations of model *N*-linked oligosaccharides are proposed to be sufficient to achieve thermodynamic equilibrium of molecular motions and are in agreement with the experiment. In these simulations, *N*-linked trimannosyl core linkage and ring dynamics were sensitive to terminal substitution, stacked rings in sialyl Lewis antennae were unusually rigid and all other pyranoses exhibited unique and flexible conformational equilibria. Considered alongside previous studies of linear glycosaminoglycans,^24^ these findings for branched *N*-glycans finalize initial exploration of μs-dynamics in model glycomic sequences and implicate linkage position, secondary-structure, and puckering in functional diversity. The experimentally-consistent results herein suggest that 10 μs unbiased explicit solvent simulations can be applied routinely to derive accurate oligosaccharide conformational information for use in structure-based design of novel chemical probes, drugs, and biomaterials.

## 4 Computational methods

### 4.1 Simulations

Explicit solvent molecular dynamics simulations of **1**–**8** (Fig. 1) were performed for 10 μs each using NVIDIA GeForce GTX TITAN graphics processors (Kepler architecture) and ACEMD^44^ (Acellera Ltd), 25 μs were performed for **2**. The molecules and water solvent were modeled using the standard GLYCAM06^26^ and TIP3P^41^ force fields, respectively. Each carbohydrate was solvated in an explicit water box of equal side length using the Amber12^45^ tool tleap, resulting in water boxes of ≈40 Å.^3^ Solute atoms were positioned at least 12 Å from the solvent box edge and the assemblies were neutralized, where appropriate, by adding explicit Na^+^ ions. Following initial conjugate-gradient energy minimization (1000 steps), each assembly was heated from 0 to 298 K and equilibrated in the NPT ensemble (for 20 ns) prior to NVT production dynamics, the first 250 ns were discarded and data were recorded at 10 ps intervals. The velocity-Verlet integration algorithm and a hydrogen mass repartitioning scheme allowed a 4 fs time-step without affecting the equilibrium distribution.^46^ Hydrogen atoms were constrained (using M-SHAKE^47^) and electrostatic interactions were calculated via the particle mesh Ewald method with a grid spacing of less than or equal to 1.0 Å (in the *X*, *Y* and *Z* dimensions). Electrostatic and van der Waals interactions were truncated at 9 Å and the recommended scaling factor for carbohydrate 1–4 interactions (1.0) was employed.^26^ Oligosaccharide trajectories were generated at a rate of ≈250 ns per day using one graphics processing unit.

### 4.2 Molecular properties

Complete 10 μs trajectories (i.e., 1,000,000 data points) were used to compute all molecular properties (25 μs for **2**). For comparison, linkage dynamics were also analyzed using only the initial 100 ns. The *ϕ* and *ψ* torsions in (1→*n*) linkages (where *n* = 2, 3, 4, or 6) were computed using the IUPAC formalism *ϕ* = O_5_–C_1_–O–C*_n_* and *ψ* = C_1_–O–C*_n_*–C_(_*_n_* _−_ _1)_. The α(2→3) torsions were defined as *ϕ* = O_6_–C_2_–O–C_3_ and *ψ* = C_2_–O–C_3_–C_2_ and the α(1→6) *ω* angle was defined as O_6_–C_6_–C_5_–C_4_. Periodicity was accounted for when computing torsion averages and standard deviations. Puckers were quantified using the Cremer–Pople^48^ parameters *θ*, *φ*, and *Q* (derived using the GROMACS^49^ tool g_puckering). Here, the *θ* angle was defined as O_5_–C_1_–C_2_–C_3_–C_4_–C_5_ (O_6_–C_2_–C_3_–C_4_–C_5_–C_6_ in NeuAc) and convergence was monitored by inspection of 〈*cosθ*〉. Relative free energies (Δ*G*) of binned linkages and puckers were computed, as previously,^15^, ^16^, ^22^, ^23^, ^24^ using the standard relationship Δ*G* = *kT* ln(*p*_1_/*p*_2_), where *k* is the Boltzmann constant, *T* is temperature (289 K), and *p* is the probability. Linkage free energy surfaces were generated by 2D-binning (*ϕ*, *ψ*) in 10° increments. Pucker populations and exchange rates were computed by 1D-binning of conformers in 15° increments of *θ* and counting transitions (as previously),^15^, ^16^, ^22^, ^23^, ^24^ 2D-binning (in Cremer–Pople parameters *θ* and *φ*) was also performed (see Supplementary data). Pyranose ring ^1^H–^1^H three-bond vicinal spin-couplings (^3^*J*_H,H_) were calculated using the substituent-adjusted Karplus equations of Altona and Haasnoot (estimated errors are reported in the Supplementary data).^50^ The Amber12^45^ tool ptraj was used to calculate the predominant oligosaccharide conformers via hierarchical 3D-clustering (with a sampling frequency of 50 and 80 frames for 10 μs and 25 μs simulations, respectively) and also radial distribution functions, with the bin spacing set to 0.05 Å. Order parameters were calculated using isotropic Reorientational Eigenmode Dynamics^51^ and fitting to a double exponential function (Supplementary data).

## 5 Experimental

### 5.1 NMR spectroscopy

Spectra for natural abundance **1** and **2** (Dextra, UK) were recorded at 25 °C using a Bruker spectrometer equipped with a *z*-gradient CPTCI cryoprobe and with ^1^H and ^13^C frequencies of 800 and 200 MHz, respectively. The [^1^H]-1D spectra were recorded with 32,768 complex points (all following numbers of points are complex), an acquisition time of 1278 ms and a dwell time of 39 μs. Standard [^1^H,^1^H]-COSY, [^1^H,^1^H]-TOCSY and heteronuclear [^1^H,^13^C]-HSQC, [^1^H,^13^C]-HMBC, and [^1^H,^13^C]-HSQC-TOCSY experiments were performed for assignment. For the heteronuclear experiments the dwell time, ^13^C carrier frequency, and ^13^C spectral width were set to 52 μs and 65 ppm and 40 ppm, respectively. A 2D [^1^H,^1^H]-NOESY spectrum of **1** and **2** was recorded with a mixing time of 600 ms, known to be appropriate from previous work.^52^ A sweep width of 11,000 Hz was used in both dimensions and the respective number of points collected in the direct and indirect dimensions were 2048 and 256. Spectra were processed and analyzed using NMRPipe^53^ and Sparky.^54^ Proton chemical shifts were referenced relative to internal DSS and heteronuclei were referenced indirectly. Appropriate linear prediction, window functions, and zero-filling were used to achieve the maximum possible resolution from each dataset.^52^

## Supplementary data

Supplementary data This document contains Supplementary figures.
