# Supplementary material for: Shaping up for structural glycomics: a predictive protocol for oligosaccharide conformational analysis applied to N-linked glycans
Source: Carbohydr Res. 2014 Jan 13;383:34–42. doi: 10.1016/j.carres.2013.10.011 (PMC3909462; doi:10.1016/j.carres.2013.10.011)
Supplement: Supplementary data — This document contains Supplementary figures. [file mmc1.pdf]

**S1**	Structural definitions used in the manuscript
**S2**	Structures of model *N*-linked oligosaccharides and constituent monosaccharides **1-8**
**S3-S9**	Torsion time series and histograms (100 ns, 10 μs and 25 μs simulations of **1-8**)
**S10-S13**	Glycosidic linkage free energy surfaces (100 ns and 10 μs simulations of **1-8**)
**S14-S20**	Ring puckering convergence and time series (10 μs and 25 μs simulations of **1-8**)
**S21-S24**	Computed ring pucker populations and free energies (10 μs simulations of **1-8**)
**S25**	Calculated and experimental NMR vicinal couplings (10 μs simulations of **1-8**)
**S26**	Water-oligosaccharide radial distribution functions (10 μs simulations of **1-2**)
**S27**	Calculation of errors in one-dimensional puckering free energy surfaces
**S28-S30**	NMR data (800 MHz) for **1** and **2** (nOes, ^{1}H and ^{13}C chemical shift assignments)
**S31**	Calculation of order parameters (S^2)
**S32-S35**	Cartesian coordinates for the most populated conformers of **1**, **2**, **7** and **8**

Figure S1. Nomenclature and structural definitions employed in the manuscript

A: Atom naming convention and example pyranose ring conformations ($^{1}C_{4}$ and $^{4}C_{1}$ chair puckers)

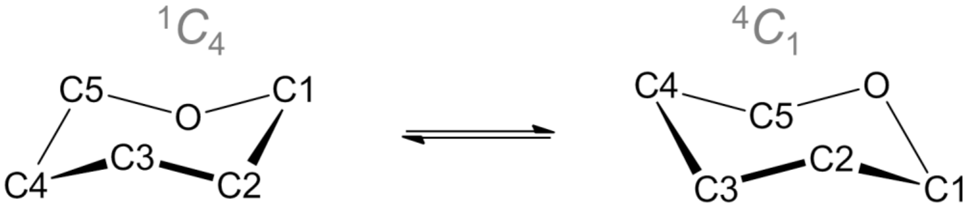

B: Oligosaccharide glycosidic linkage definitions, ϕ = O_5-C_1-O-C_n ψ = C_1-O-C_n-$C_{(n-1)}$

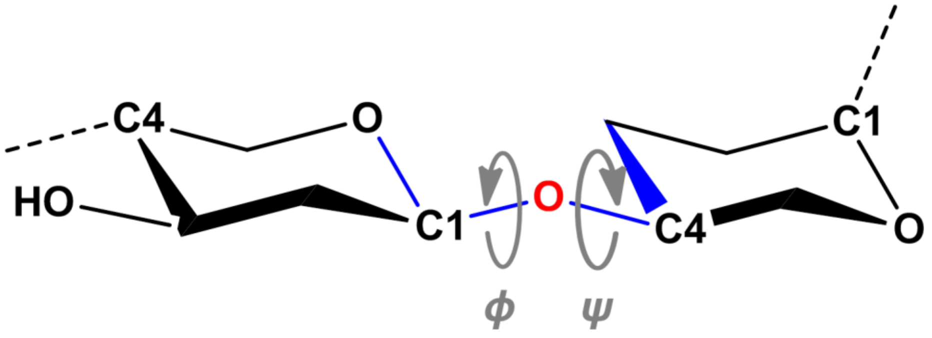

C: Example pyranose ring three bond proton-proton (^{1}H-^{1}H) vicinal spin-coupling ($^{3}J_{H,H}$)

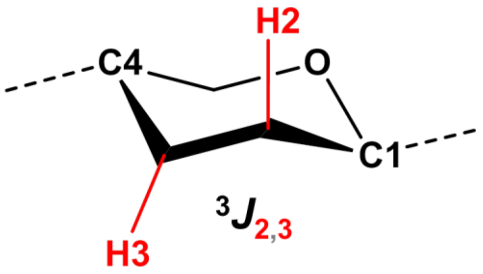

Figure S2. Structures of model oligosaccharides **1-8** (also see Figure 1 caption)

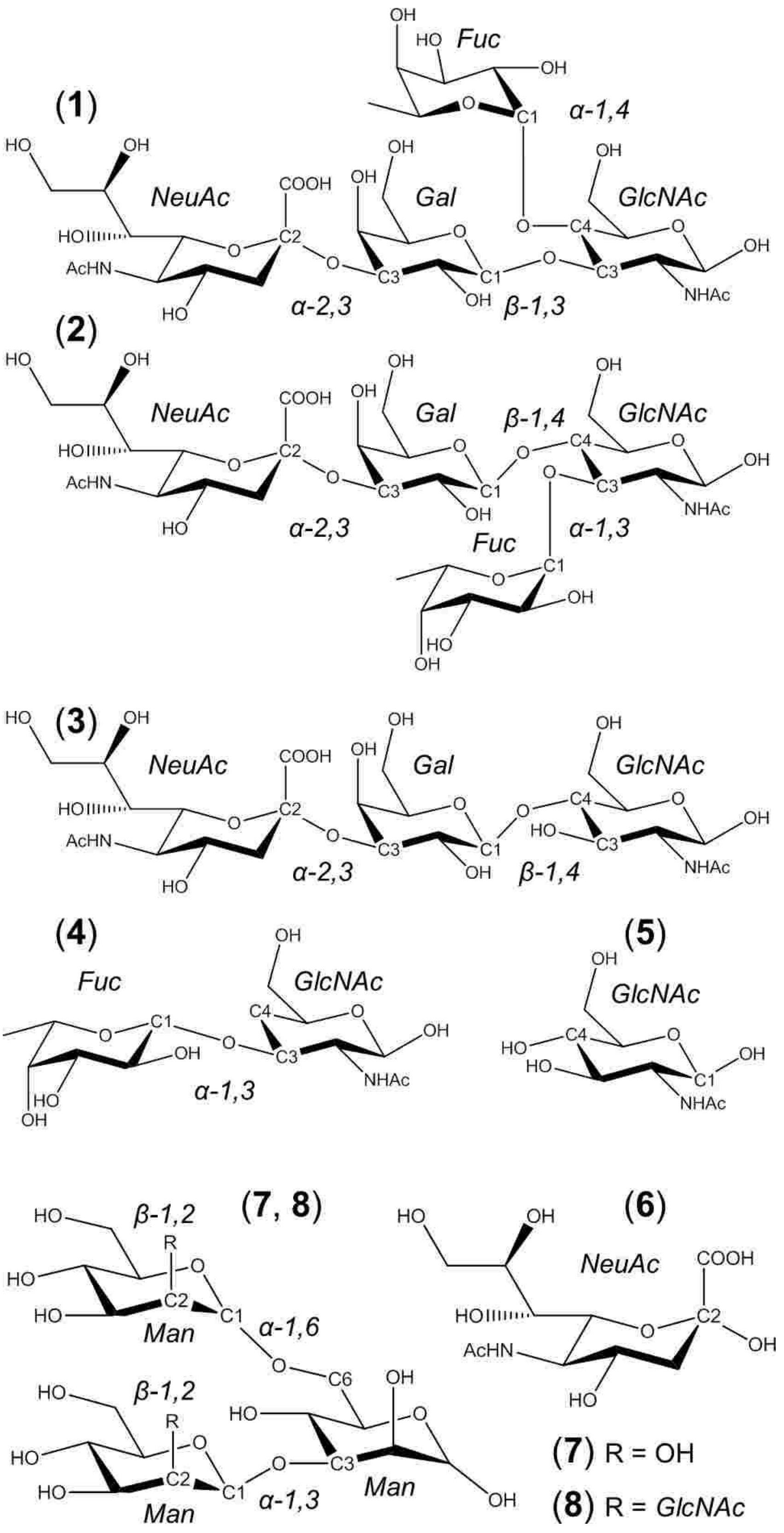

Figure S3. Time series of linkage torsions (10 µs and initial 100 ns): molecule **1**

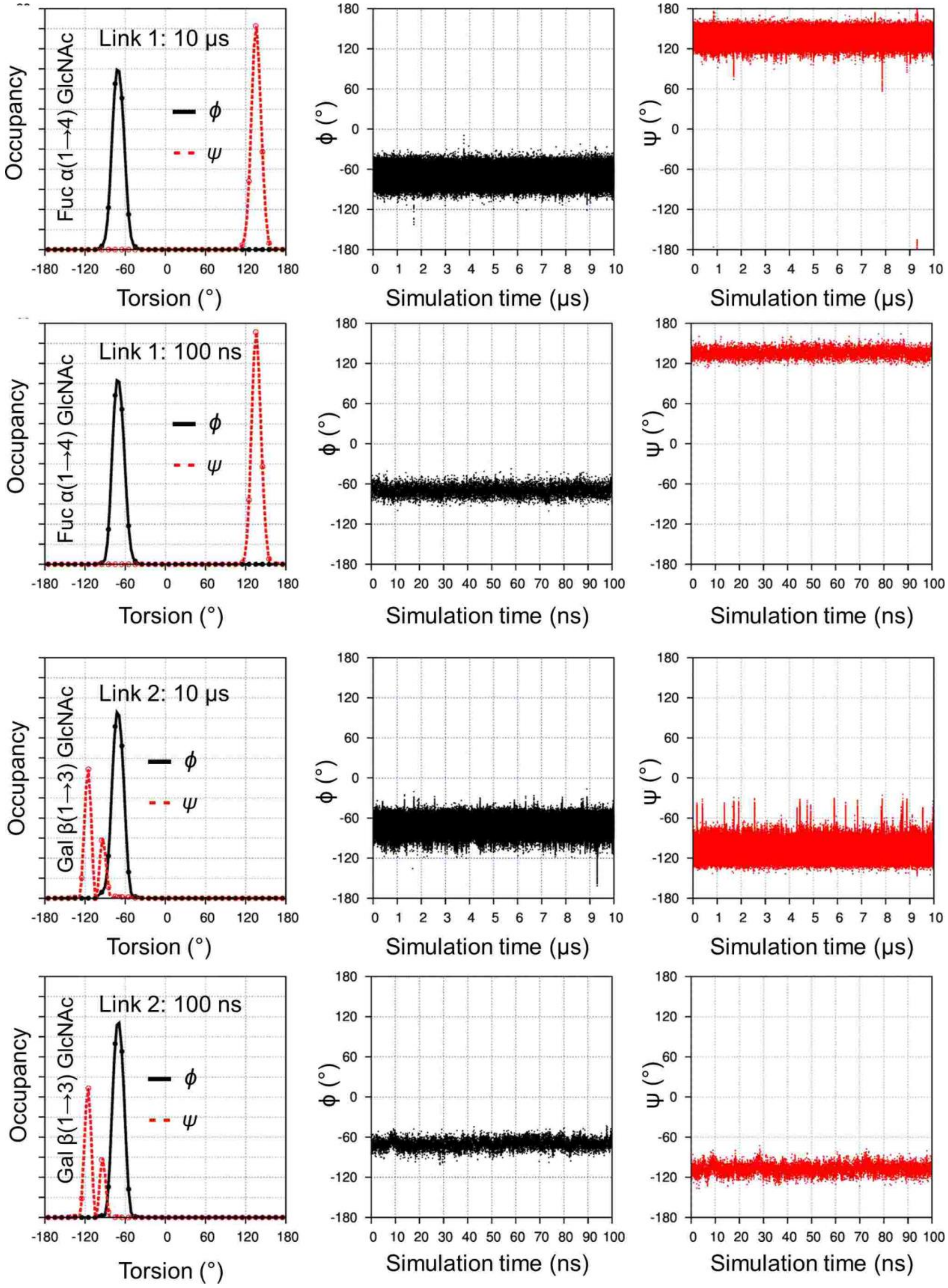

Figure S3 continued

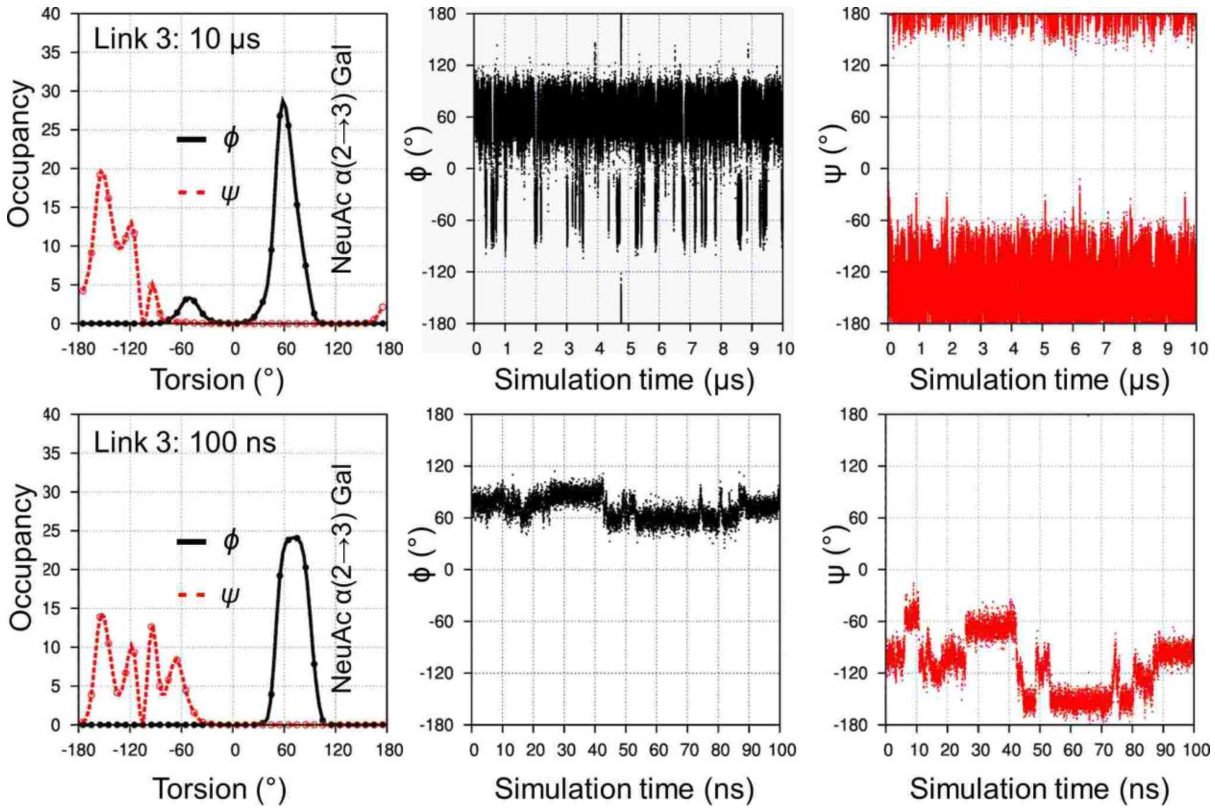

Figure S4. Time series of linkage torsions (10 μs and initial 100 ns): molecule **2**

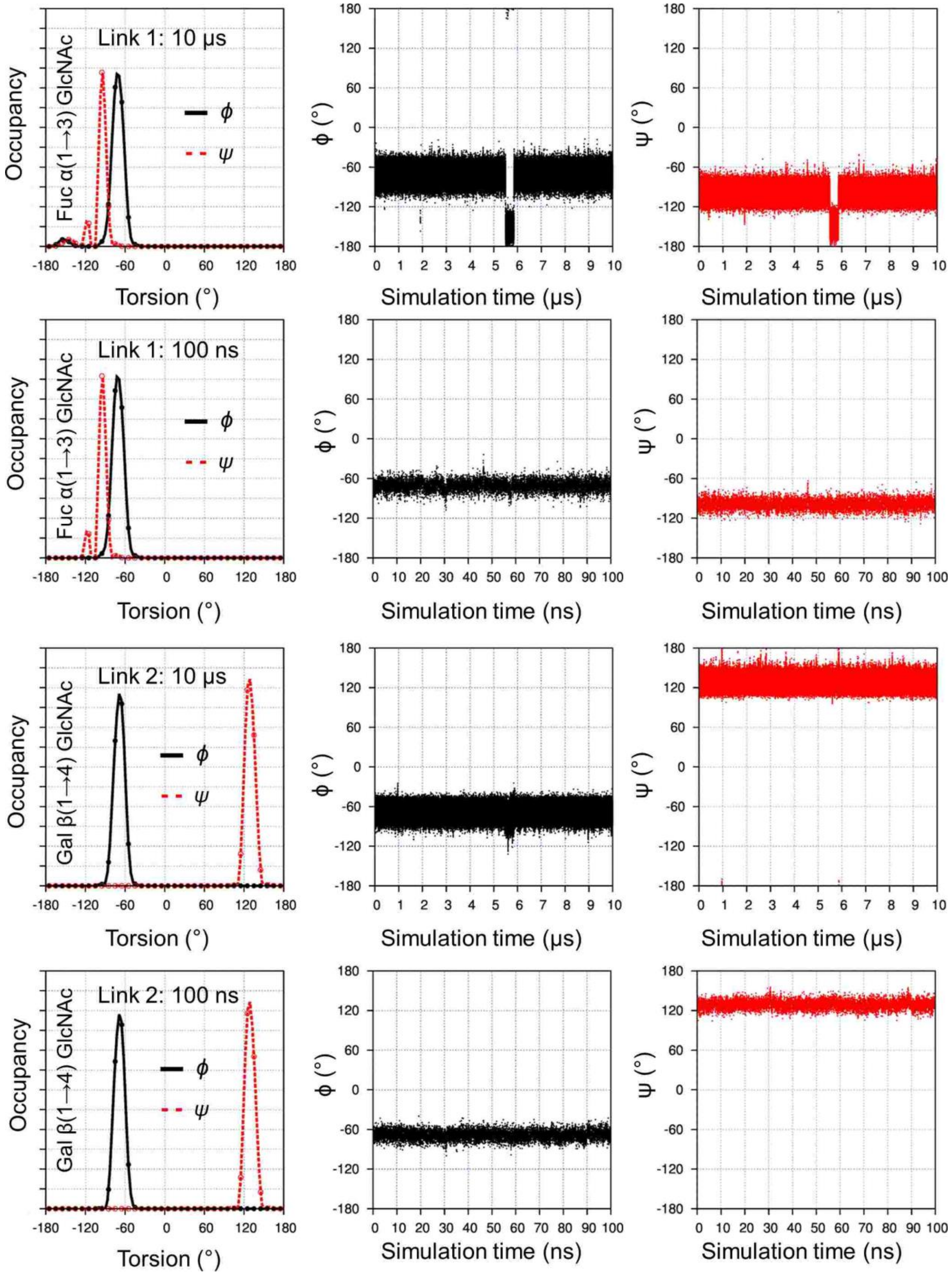

Figure S4 continued

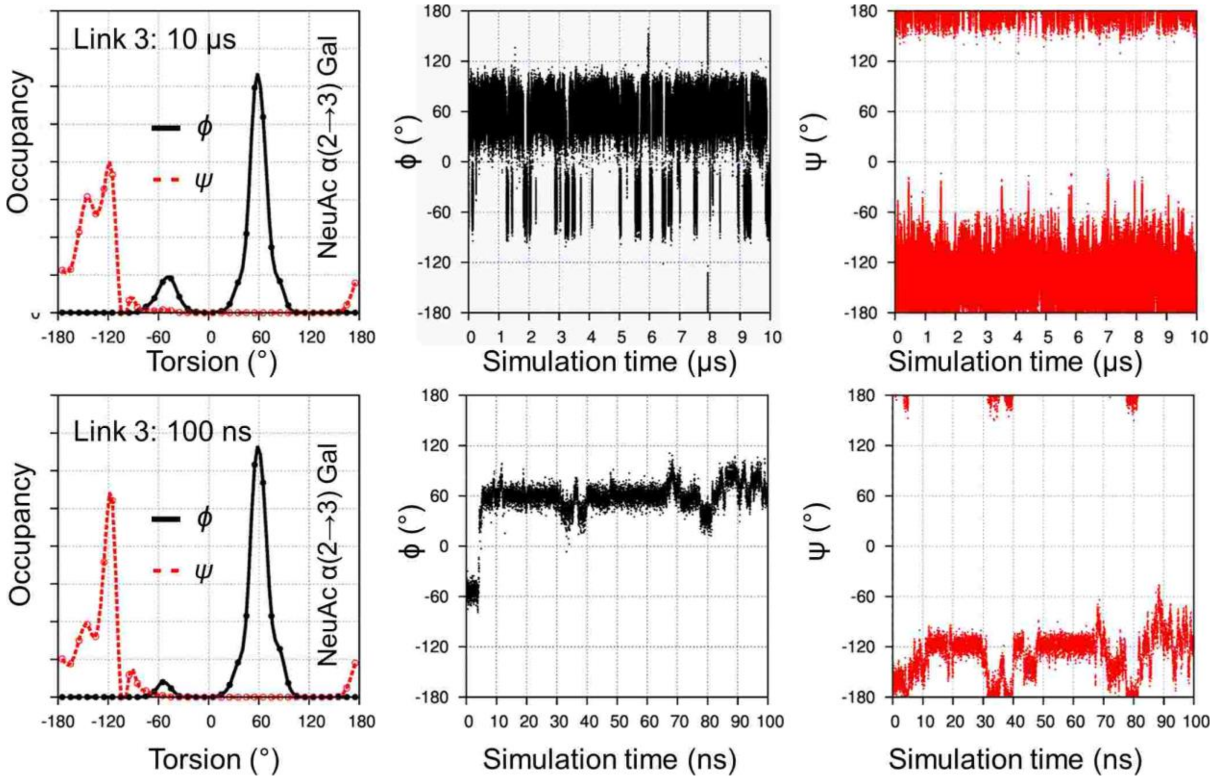

Figure S5. Time series of linkage torsions from a 25 µs simulation of sLe^{x}: molecule **2**

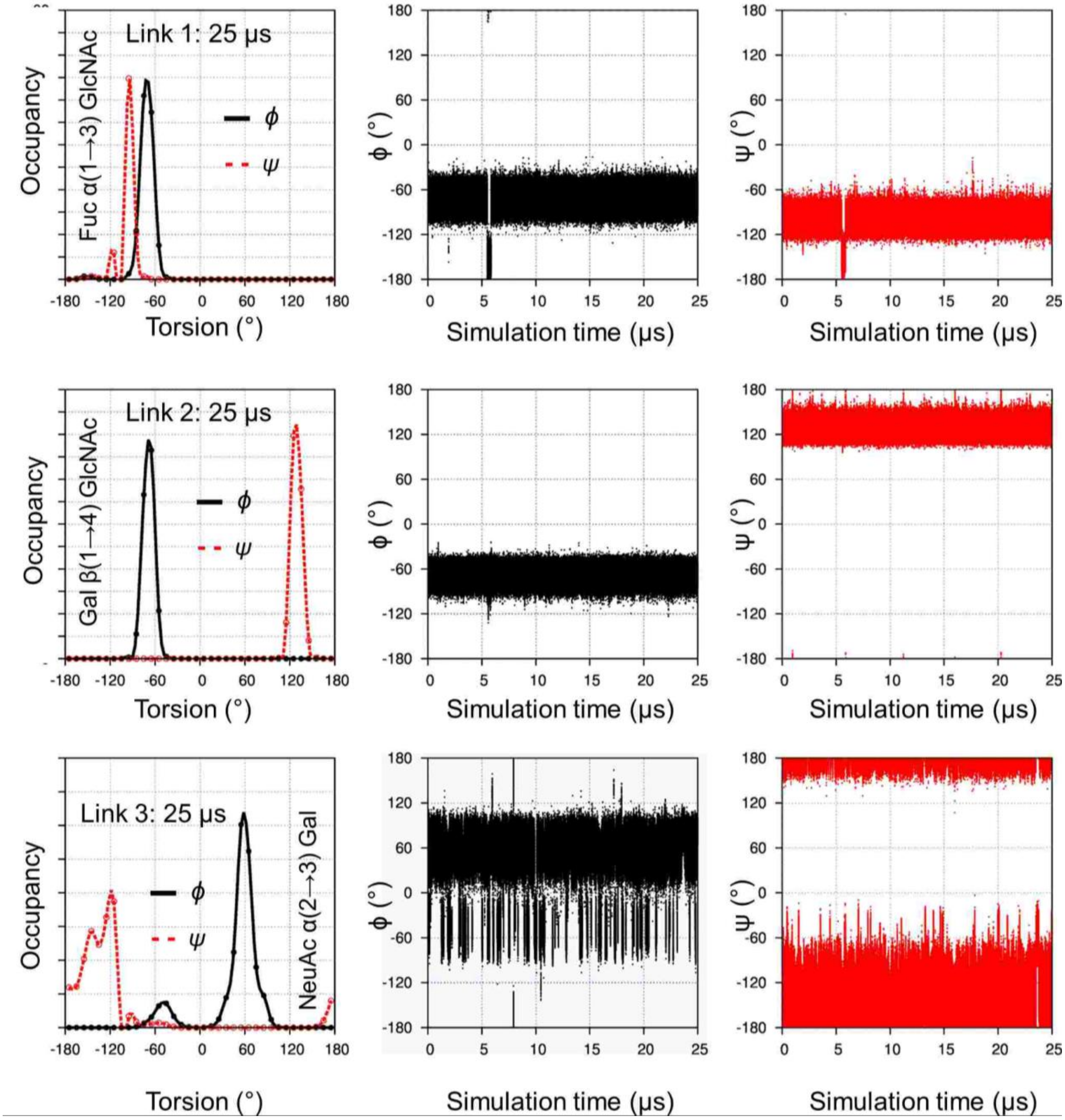

Figure S6. Time series of linkage torsions (10 µs and initial 100 ns): molecule **3**

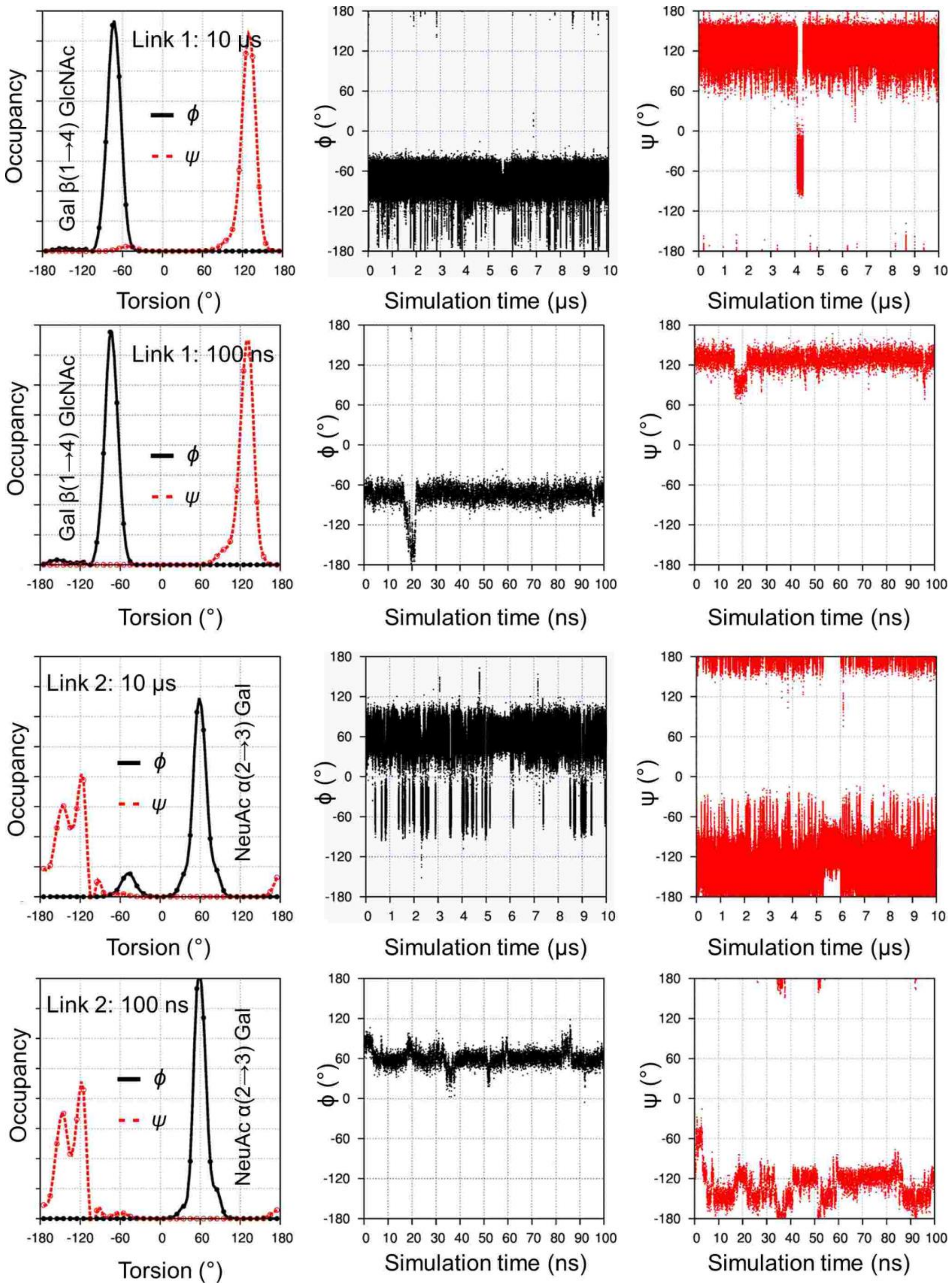

Figure S7. Time series of linkage torsions (10 µs and initial 100 ns): molecule **4**

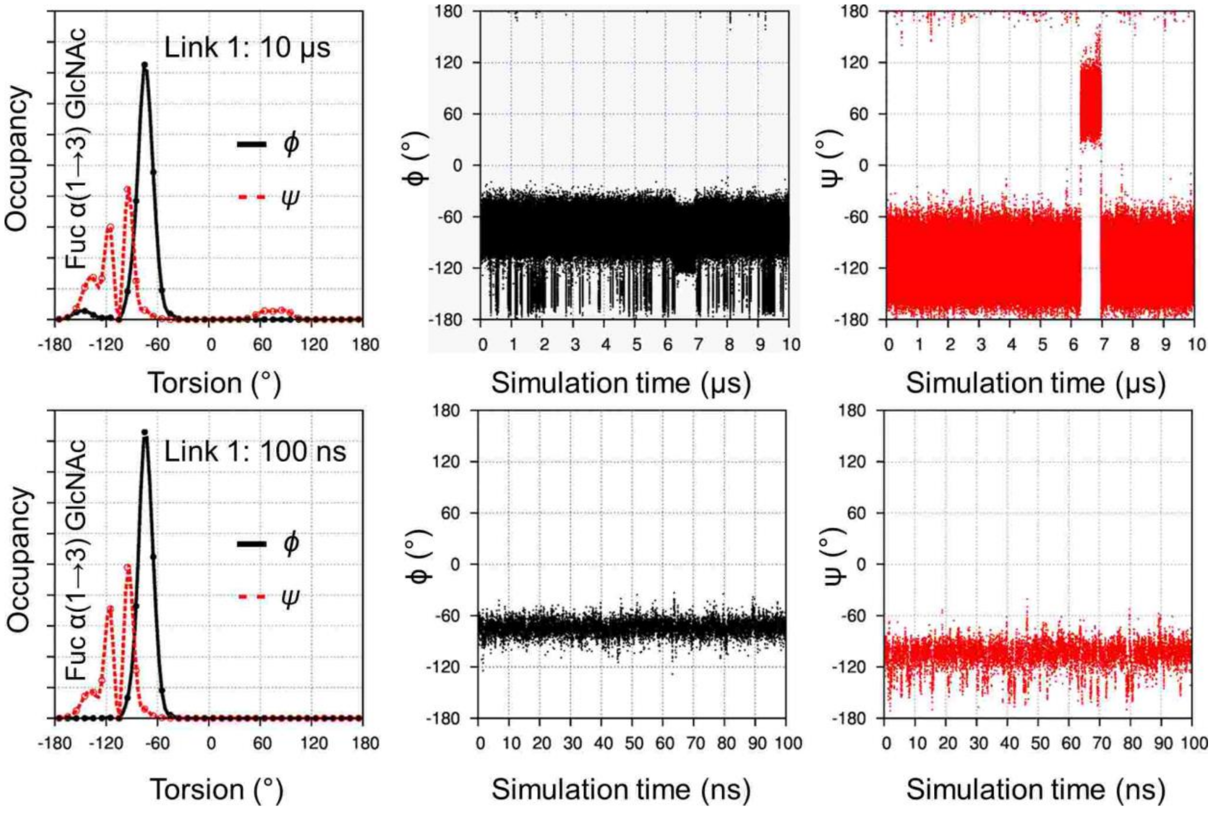

Figure S8. Time series of linkage torsions (10 µs and initial 100 ns): molecule **7**

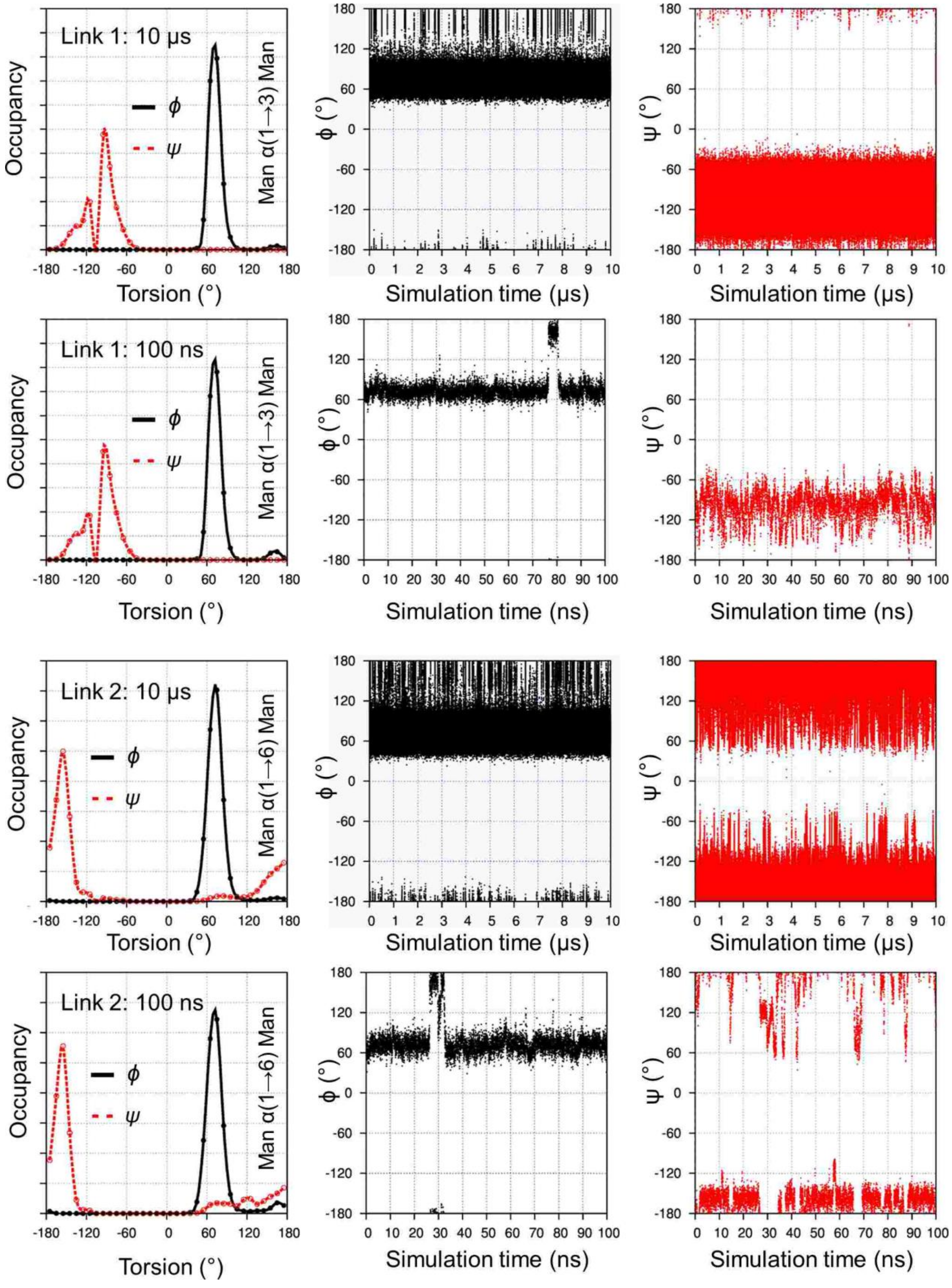

Figure S9. Time series of linkage torsions (10 µs and initial 100 ns): molecule **8**

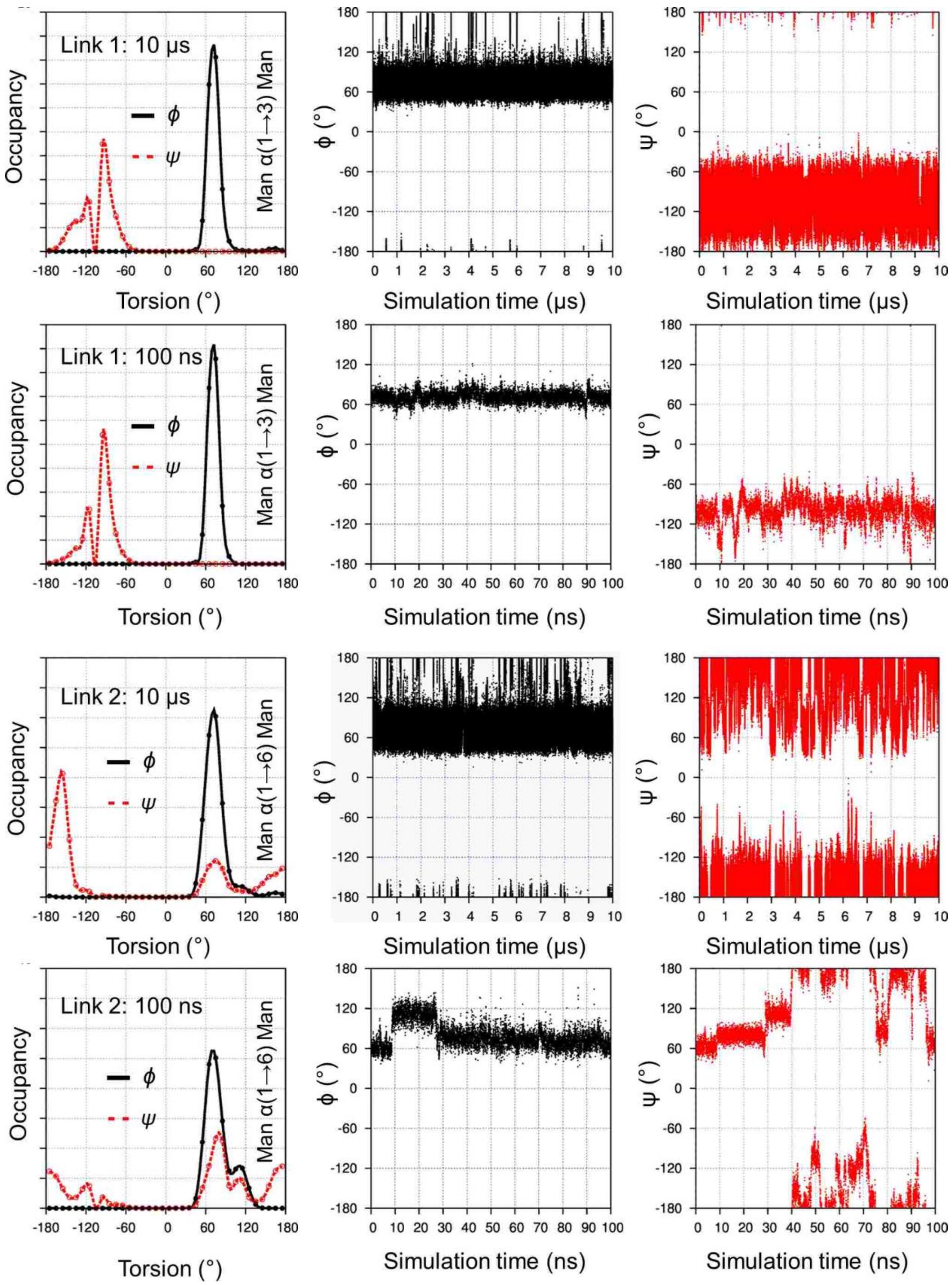

Figure S9 continued

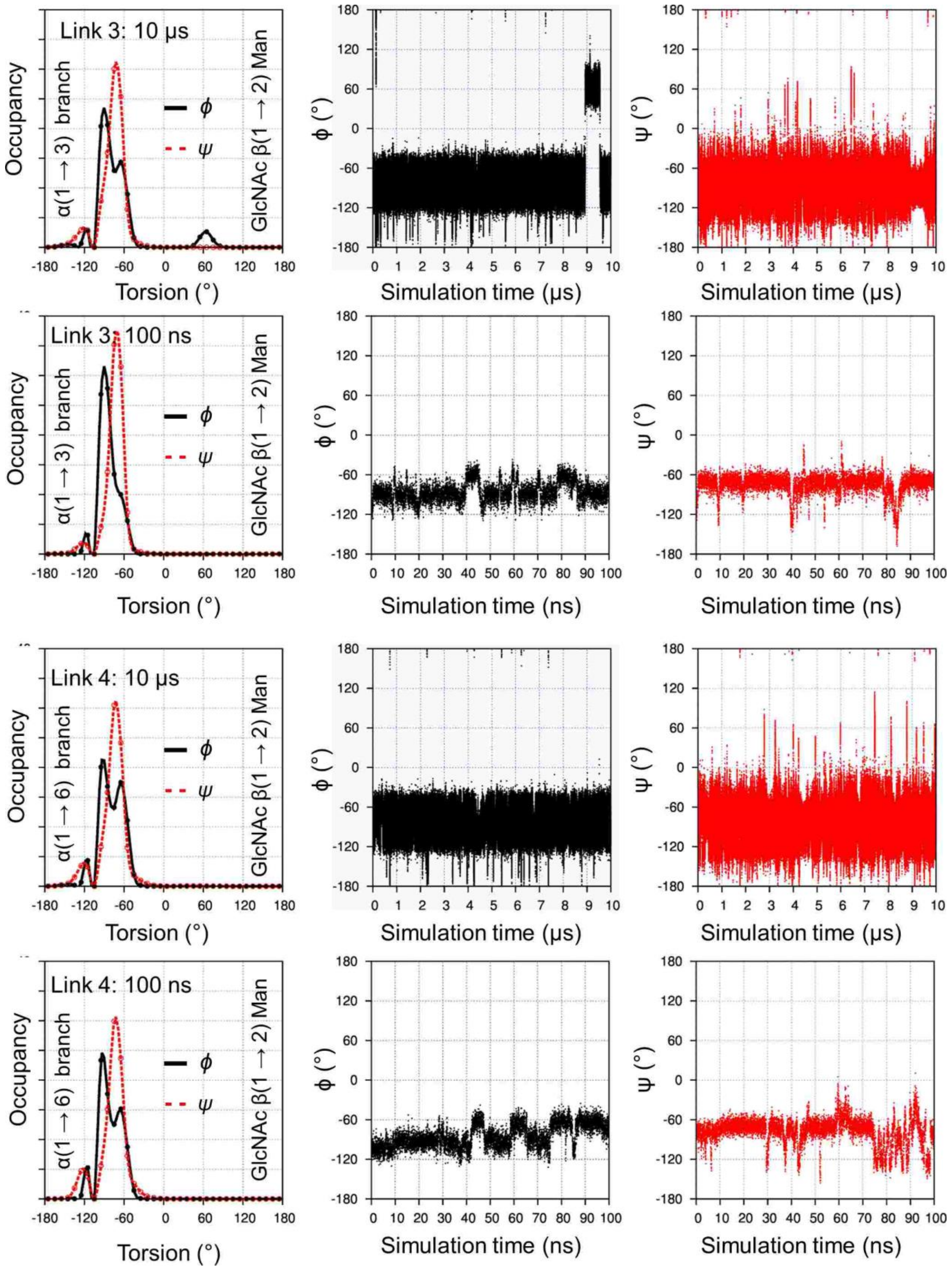

Figure S10. Linkage free energy surfaces (10 µs): molecules **1-4** (contours at 2, 4 and 6 kcal mol^{-1})

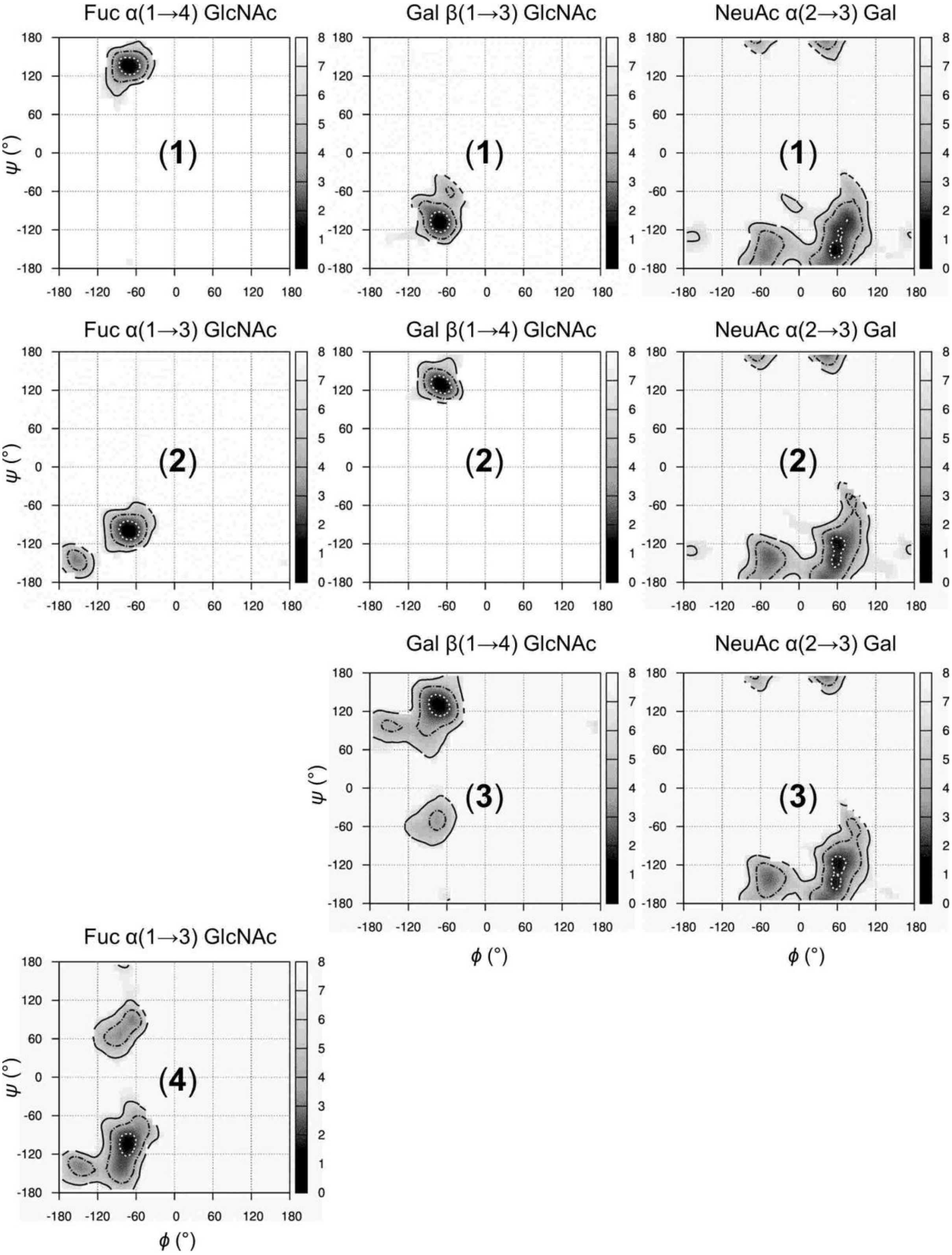

Figure S11. Linkage free energy surfaces (100 ns): molecules **1-4** (contours at 2, 4 and 6 kcal mol^{-1})

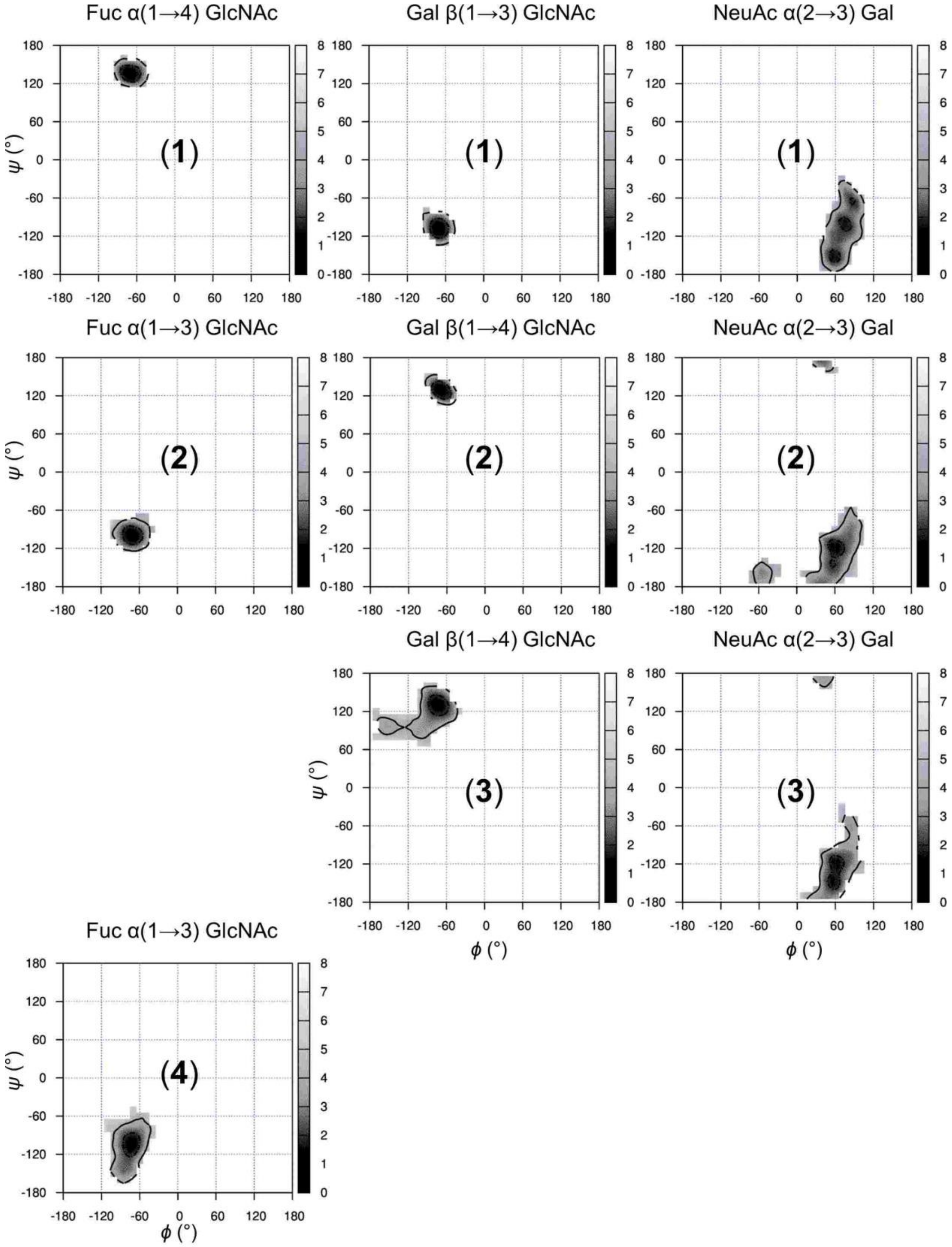

Figure S12. Linkage free energy surfaces (10 μs): molecules **7-8** (contours at 2, 4 and 6 kcal mol^{-1})

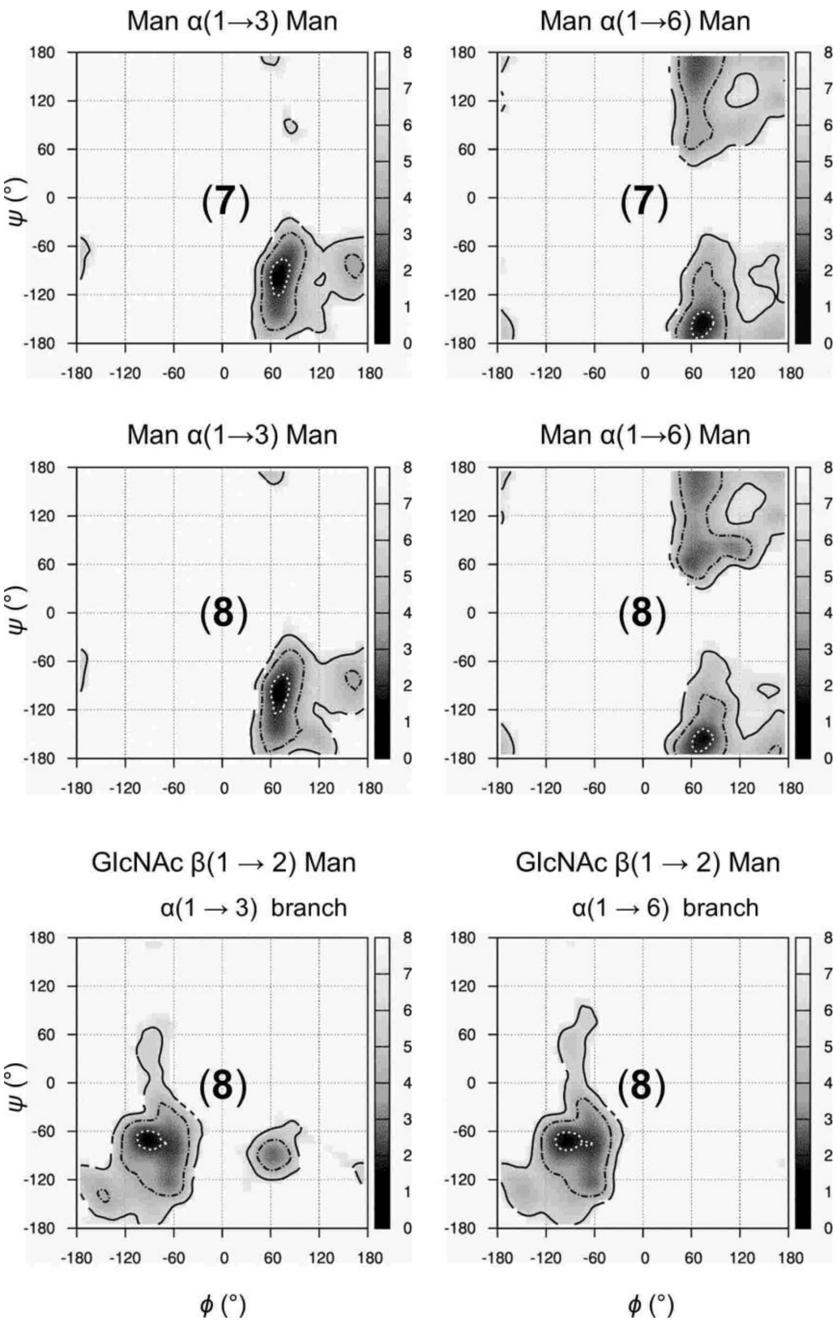

Computed average α(1-6) ω torsions in **7** and **8**: (also see Table 1 Legend)

**Torsion**		**CAL (μs)**		**CAL (ns)**		**NMR**[1]	
**α(1-6)**	**Mol**	***ω*** **(±σ)**	**min**	***ω*** **(±σ)**	**min**	***ω*** **(±σ)**	**min**
Man-Man	**7**	54 (52)	2	53 (10)	1	60	>1
Man-Man	**8**	51 (41)	2	-120 (99)	2	60	>1

(1) Sayers, E. W.; Prestegard, J. H. *Biophys J* **2000**, *79*, 3313.

Figure S13. Linkage free energy surfaces (100 ns): molecules **7-8** (contours at 2, 4 and 6 kcal mol^{-1})

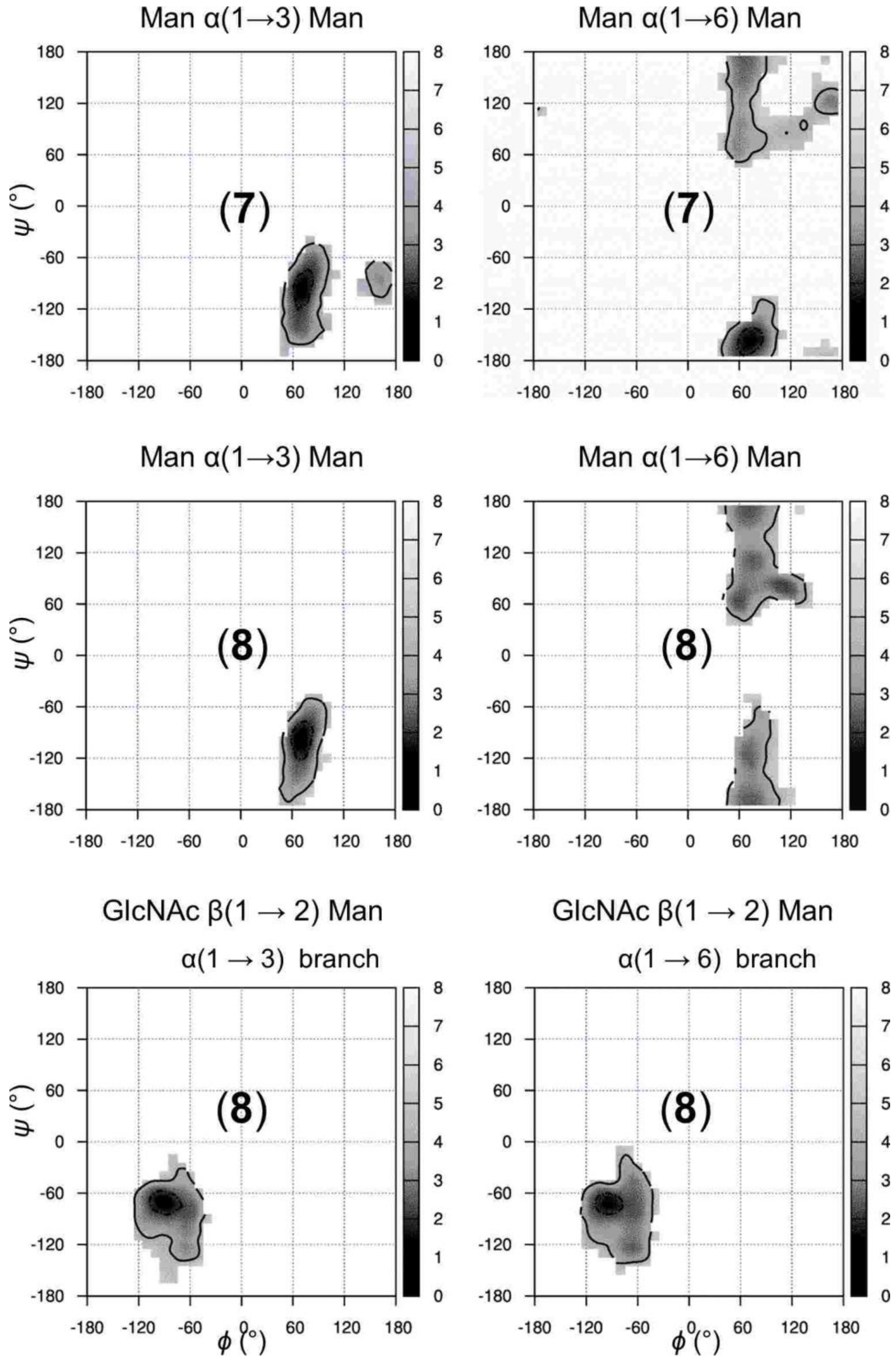

Figure S14. Puckering convergence (red line denotes simulation average) and time series: molecule **1**

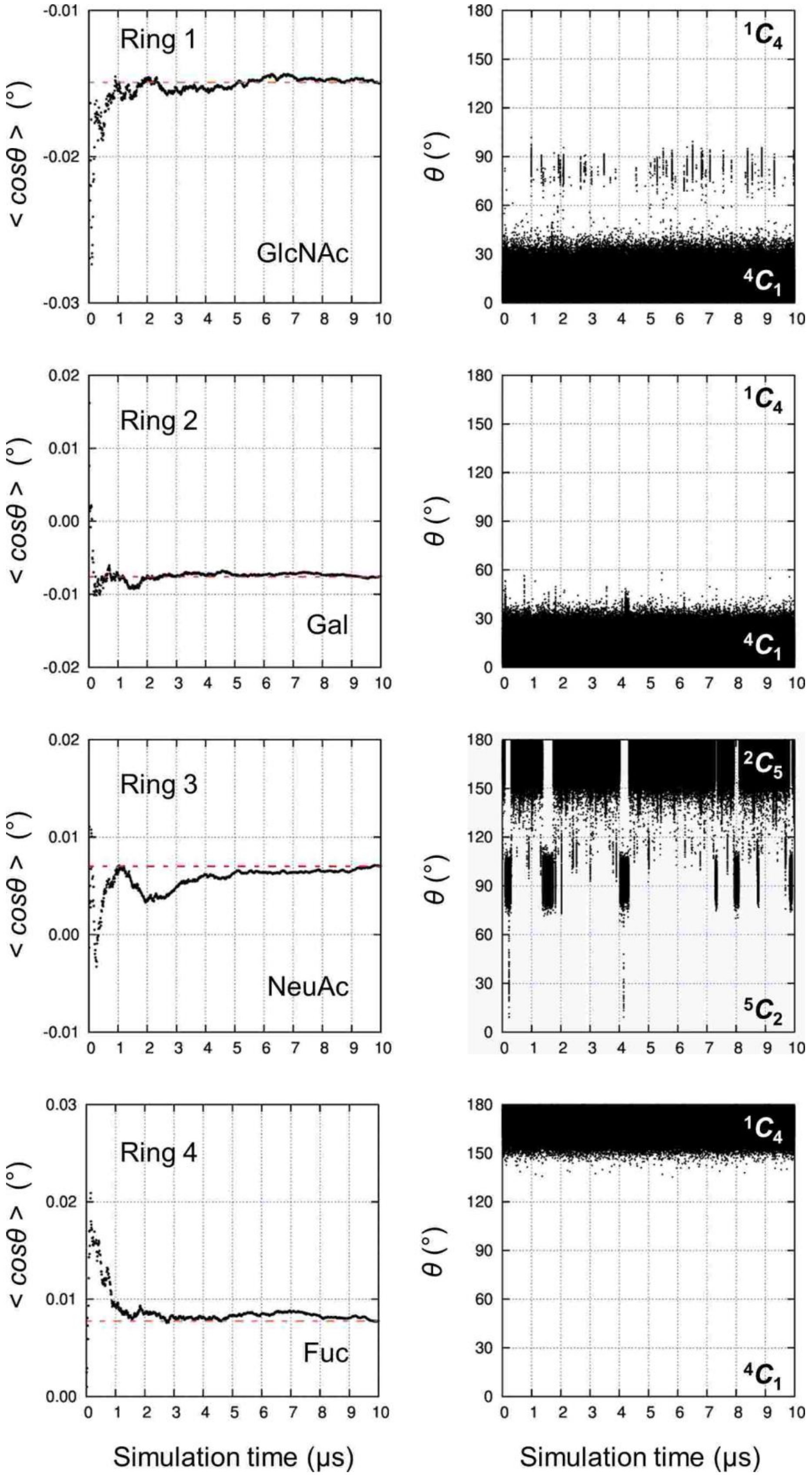

Figure S15. Puckering convergence (red line denotes simulation average) and time series: molecule **2**

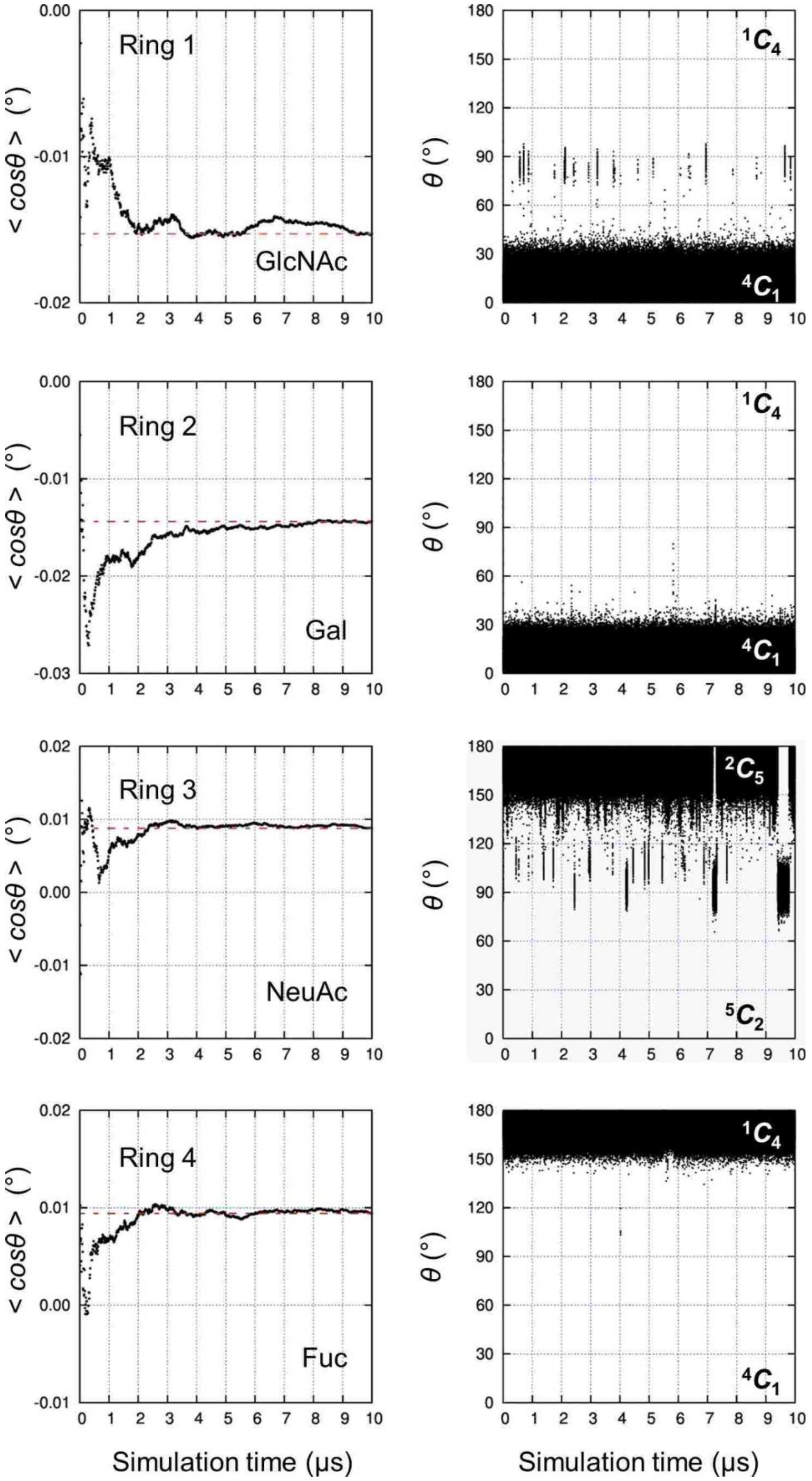

Figure S16. Puckering convergence (red line denotes simulation average) and time series: molecule **2**

25 μs simulation

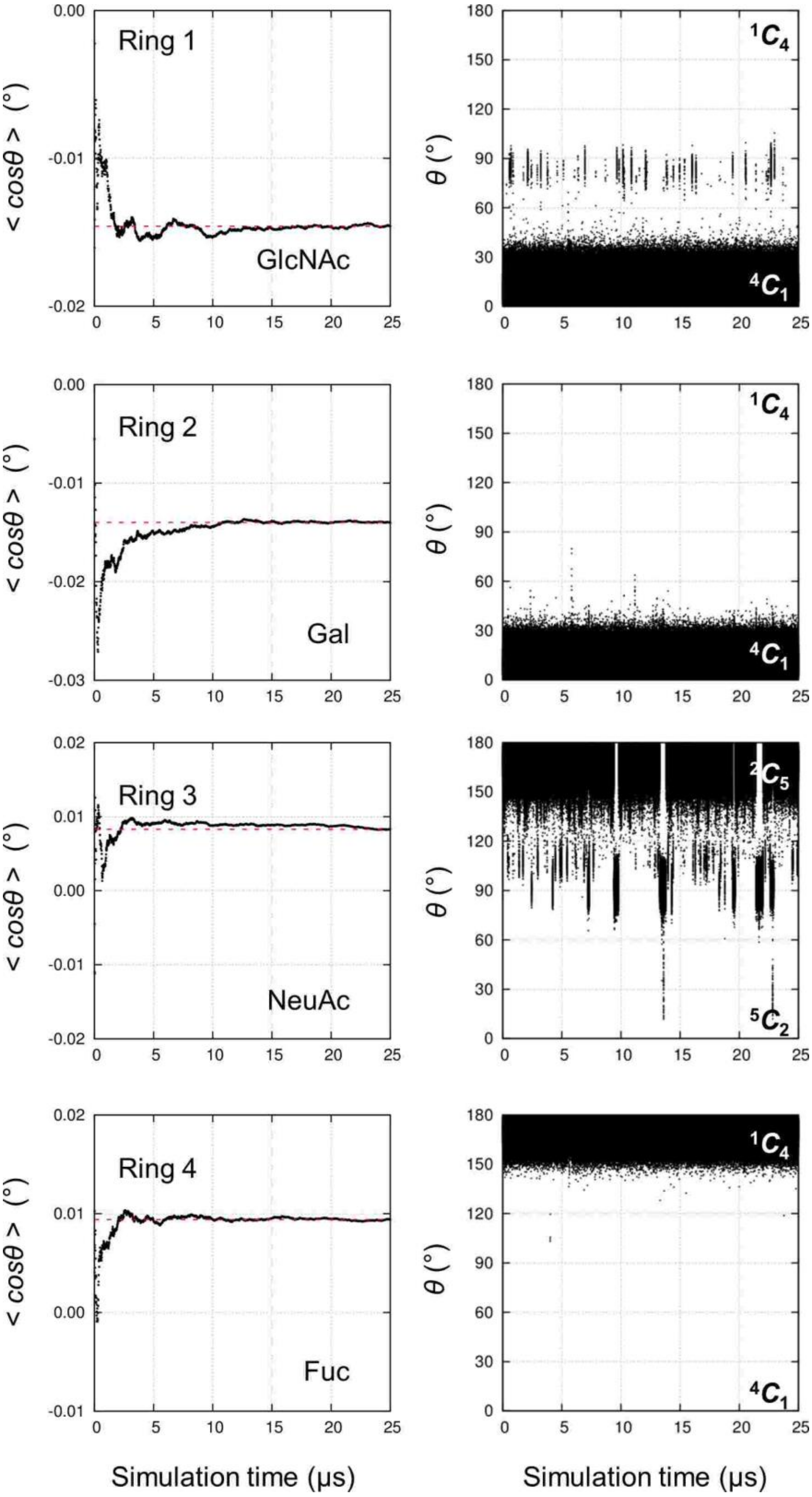

Figure S17. Puckering convergence (red line denotes simulation average) and time series: molecule **3**

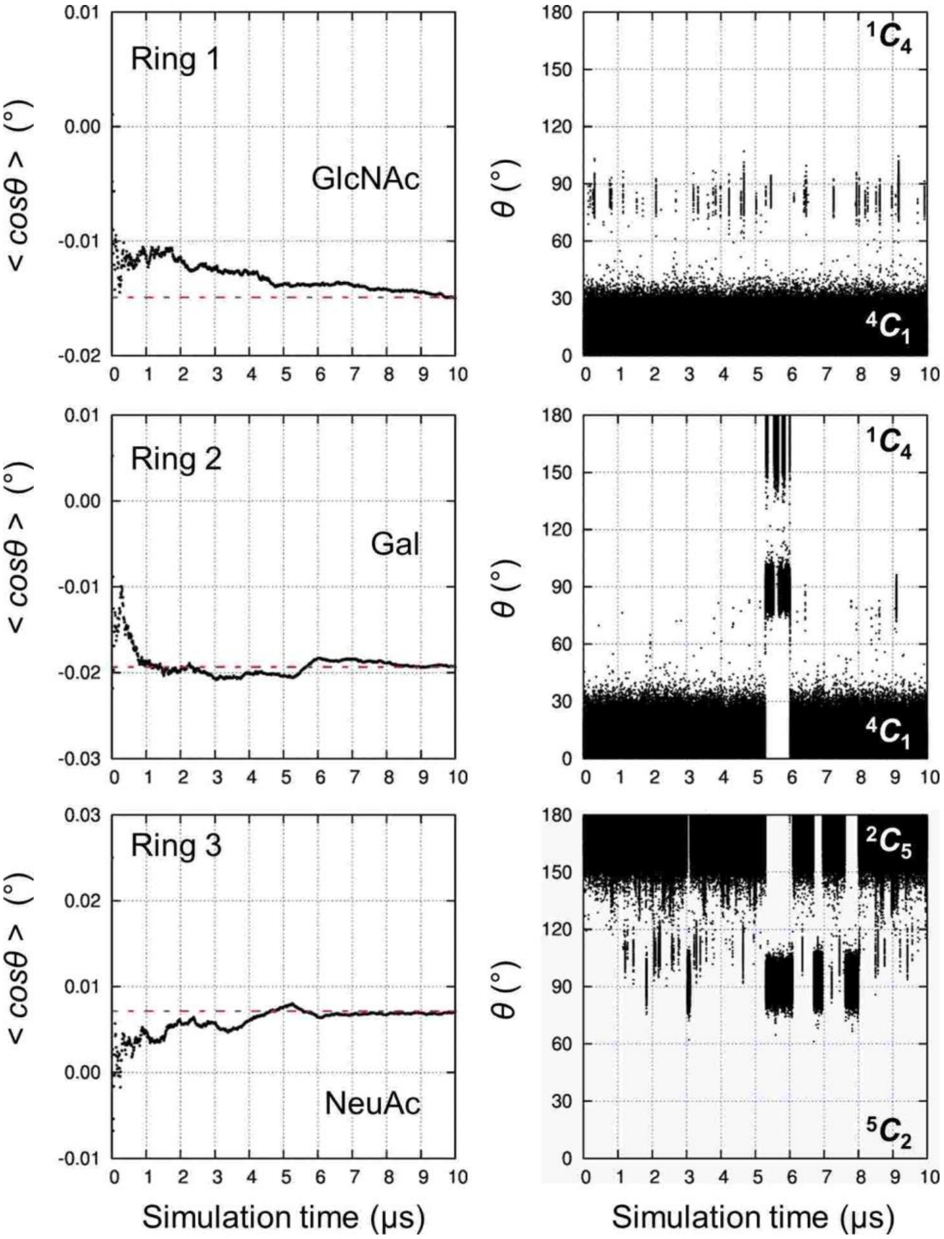

Figure S18. Puckering convergence (red line denotes simulation average) and time series: mols **4**-**6**

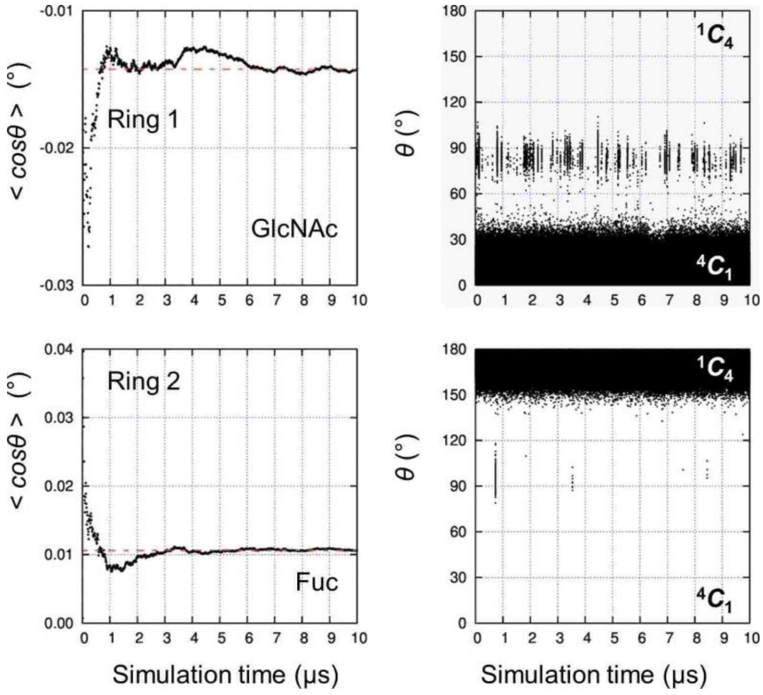

GlcNAc (**5**)

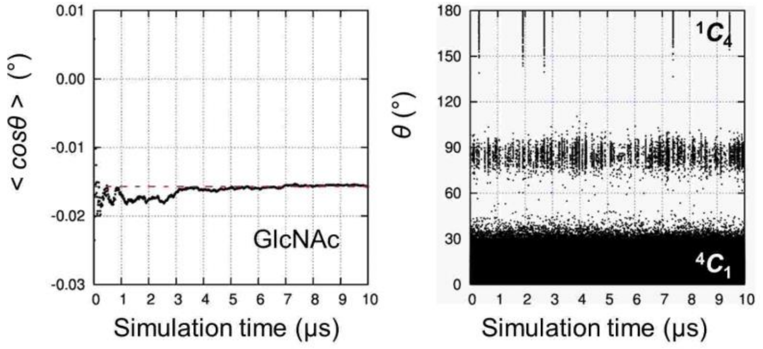

NeuAc (**6**)

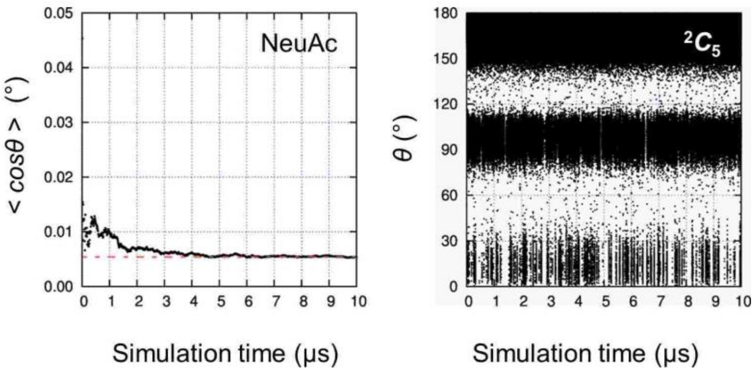

Figure S19. Puckering convergence (red line denotes simulation average) and time series: molecule **7**

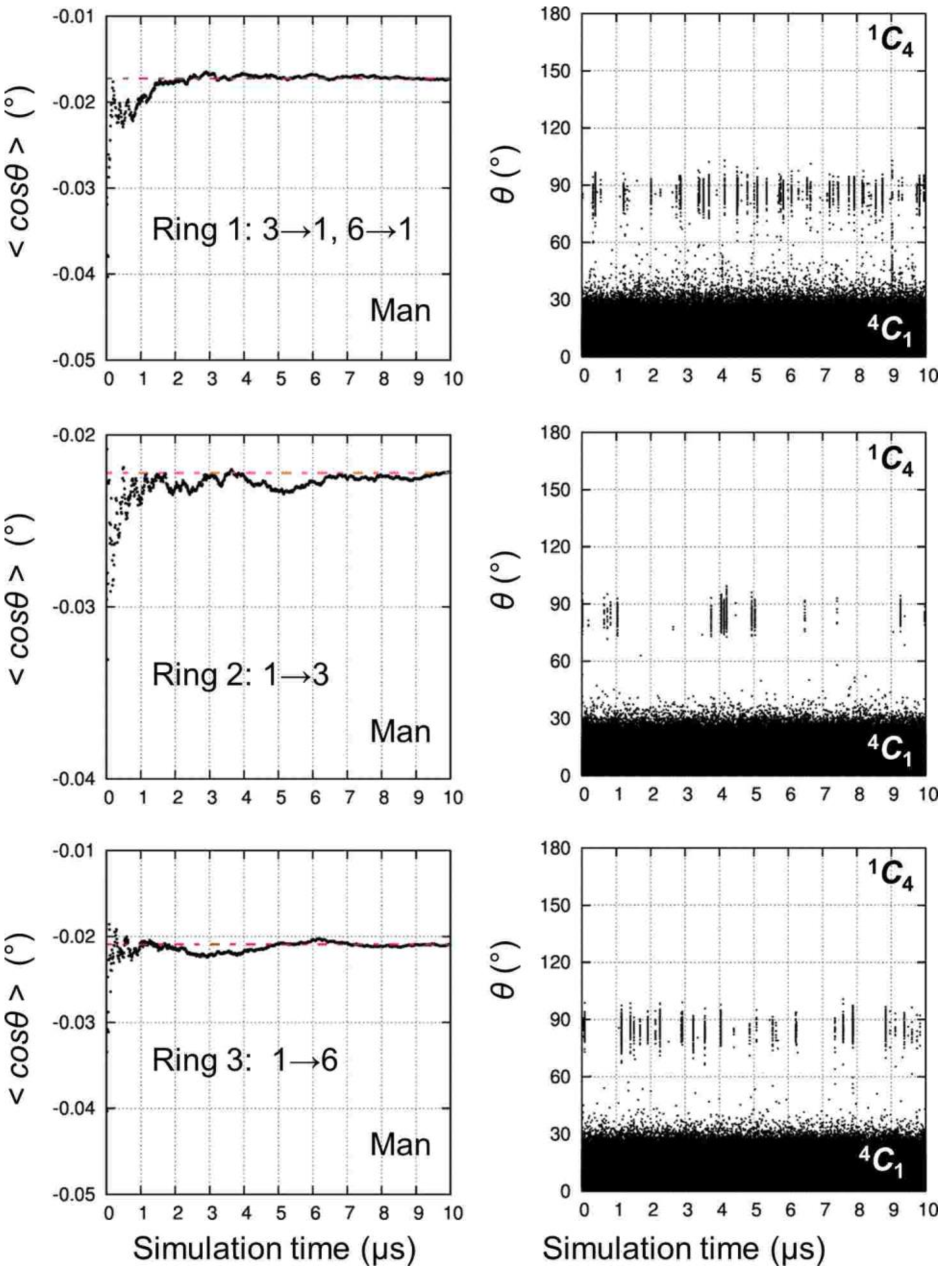

Figure S20. Puckering convergence (red line denotes simulation average) and time series: molecule **8**

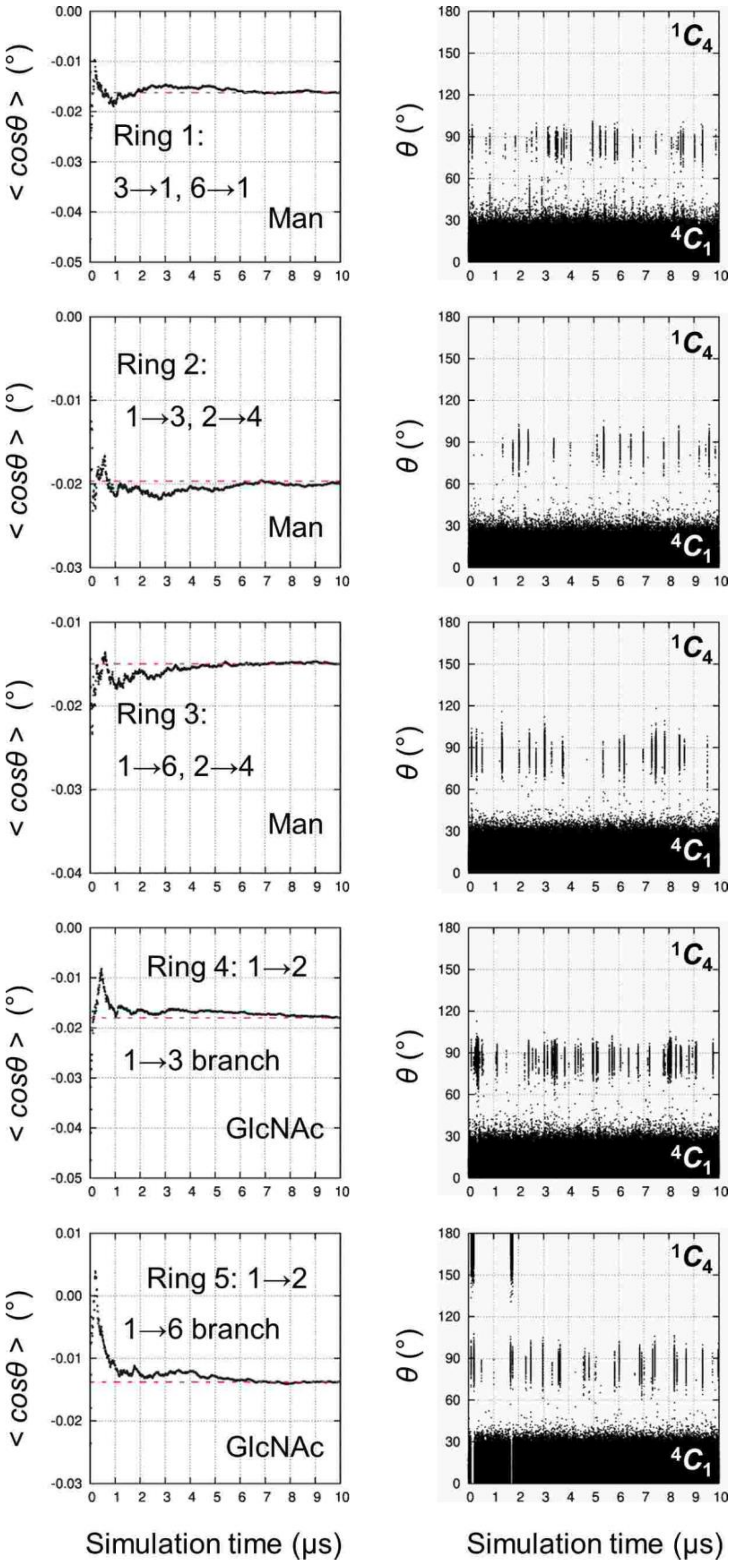

Table S21. Calculated pyranose ring puckering populations (%) and relative free energies (Δ*G*, kcal mol^{-1}) from the 10 µs simulations: GlcNAc

Mol (**1**) GlcNAc

**Puck**	**%**	**Δ*G***
**4C1**	99.7310	0.00
**1S3**	0.0717	4.29
**B3O**	0.0369	4.69
**14B**	0.0277	4.86
**E5**	0.0241	4.94
**4H5**	0.0177	5.12
**OH5**	0.0158	5.19
**2SO**	0.0140	5.26
**4H3**	0.0131	5.30
**4E**	0.0127	5.32
**E3**	0.0081	5.59
**OE**	0.0072	5.66
**2H3**	0.0053	5.84
**1S5**	0.0038	6.04
**OH1**	0.0037	6.05
**2E**	0.0031	6.16
**2H1**	0.0024	6.31
**E1**	0.0016	6.55
**25B**	0.0001	8.19

Mol (**2**) GlcNAc

**Puck**	**%**	**Δ*G***
**4C1**	99.8474	0.00
**1S3**	0.0289	4.83
**14B**	0.0282	4.85
**1S5**	0.0181	5.11
**B3O**	0.0126	5.32
**4H5**	0.0112	5.39
**E5**	0.0109	5.41
**OH5**	0.0089	5.53
**4H3**	0.0064	5.73
**4E**	0.0060	5.76
**E3**	0.0049	5.88
**OE**	0.0045	5.94
**2SO**	0.0036	6.07
**2H3**	0.0028	6.22
**2E**	0.0018	6.48
**OH1**	0.0016	6.55
**E1**	0.0014	6.63
**2H1**	0.0008	6.96

Mol (**3**) GlcNAc

**Puck**	**%**	**Δ*G***
**4C1**	99.6651	0.00
**OS2**	0.0579	4.42
**1S3**	0.0562	4.44
**3OB**	0.0444	4.58
**14B**	0.0323	4.77
**3S1**	0.0298	4.81
**B3O**	0.0230	4.97
**1S5**	0.0141	5.26
**2SO**	0.0113	5.39
**4H3**	0.0099	5.47
**4E**	0.0070	5.67
**OH5**	0.0065	5.72
**4H5**	0.0061	5.75
**E3**	0.0061	5.75
**B25**	0.0056	5.81
**OE**	0.0056	5.81
**E5**	0.0055	5.82
**OH1**	0.0038	6.04
**2H3**	0.0028	6.22
**2E**	0.0027	6.24
**E1**	0.0026	6.26
**2H1**	0.0009	6.89
**B14**	0.0007	7.04
**25B**	0.0001	8.19

Mol (**4**) GlcNAc

**Puck**	**%**	**Δ*G***
**4C1**	99.4440	0.00
**1S3**	0.1346	3.92
**B3O**	0.0640	4.36
**3S1**	0.0637	4.36
**2SO**	0.0568	4.43
**14B**	0.0453	4.57
**3OB**	0.0223	4.99
**OH5**	0.0160	5.18
**OE**	0.0154	5.21
**1S5**	0.0145	5.24
**E3**	0.0144	5.25
**OH1**	0.0141	5.26
**B14**	0.0137	5.28
**E5**	0.0111	5.40
**2H3**	0.0111	5.40
**4H3**	0.0106	5.43
**5S1**	0.0084	5.57
**4H5**	0.0075	5.63
**2E**	0.0070	5.67
**4E**	0.0065	5.72
**E1**	0.0064	5.73
**OS2**	0.0047	5.91
**2H1**	0.0039	6.02
**25B**	0.0021	6.39
**B25**	0.0018	6.48
**1HO**	0.0001	8.19

Mol (**8**) 1→3 branch GlcNAc

**Puck**	**%**	**Δ*G***
**4C1**	98.8646	0.01
**B3O**	0.3325	3.38
**2SO**	0.2553	3.54
**1S3**	0.1865	3.73
**14B**	0.1283	3.95
**3S1**	0.0579	4.42
**1S5**	0.0497	4.51
**3OB**	0.0252	4.91
**OS2**	0.0139	5.27
**OH5**	0.0121	5.35
**OE**	0.0100	5.46
**E5**	0.0076	5.62
**B14**	0.0072	5.66
**E3**	0.0072	5.66
**4H3**	0.0057	5.80
**2H3**	0.0053	5.84
**5S1**	0.0049	5.88
**OH1**	0.0042	5.98
**4H5**	0.0040	6.01
**2E**	0.0038	6.04
**2H1**	0.0035	6.08
**4E**	0.0030	6.18
**25B**	0.0028	6.22
**E1**	0.0026	6.26
**B25**	0.0017	6.51
**E4**	0.0003	7.54
**EO**	0.0001	8.19
**3H2**	0.0001	8.19

Mol (**8**) 1→6 branch GlcNAc

**Puck**	**%**	**Δ*G***
**4C1**	97.1438	0.02
**1C4**	1.8959	2.35
**B3O**	0.2735	3.50
**1S3**	0.1770	3.76
**2SO**	0.1449	3.88
**14B**	0.0728	4.28
**3S1**	0.0715	4.30
**3OB**	0.0582	4.42
**1S5**	0.0438	4.59
**OS2**	0.0347	4.72
**E5**	0.0098	5.47
**OH5**	0.0090	5.52
**OE**	0.0090	5.52
**B14**	0.0071	5.66
**E3**	0.0071	5.66
**OH1**	0.0069	5.68
**4H3**	0.0068	5.69
**4H5**	0.0053	5.84
**2H3**	0.0049	5.88
**E1**	0.0040	6.01
**2E**	0.0038	6.04
**2H1**	0.0022	6.36
**B25**	0.0021	6.39
**5S1**	0.0016	6.55
**25B**	0.0015	6.59
**4E**	0.0014	6.63
**1H2**	0.0007	7.04
**5HO**	0.0002	7.78
**5H4**	0.0002	7.78
**E2**	0.0002	7.78
**5E**	0.0001	8.19

Table S22. Calculated pyranose ring puckering populations (%) and relative free energies (Δ*G*, kcal mol^{-1}) from the 10 µs simulations: Gal and Fuc

Mol (**1**) Gal

**Puck**	**%**	**Δ*G***
**4C1**	99.9168	0.00
**E5**	0.0383	4.67
**4H5**	0.0152	5.21
**OH5**	0.0114	5.38
**4H3**	0.0084	5.57
**4E**	0.0072	5.66
**E3**	0.0023	6.33
**2H3**	0.0004	7.37

Mol (**2**) Gal

**Puck**	**%**	**Δ*G***
**4C1**	99.981	0.00
**E5**	0.005	5.83
**OH5**	0.004	5.99
**4E**	0.003	6.20
**4H5**	0.002	6.31
**4H3**	0.002	6.48
**E3**	0.002	6.59
**2H3**	0.001	7.24
**1S3**	0.000	7.78

Mol (**3**) Gal

**Puck**	**%**	**Δ*G***
**4C1**	92.6621	0.05
**OS2**	4.2504	1.87
**1C4**	2.3229	2.23
**B25**	0.2715	3.50
**3OB**	0.2158	3.64
**1S5**	0.1216	3.98
**1S3**	0.0496	4.51
**B3O**	0.0282	4.85
**14B**	0.0175	5.13
**E5**	0.0126	5.32
**4H5**	0.0120	5.35
**4E**	0.0102	5.45
**4H3**	0.0079	5.60
**EO**	0.0044	5.95
**OH5**	0.0039	6.02
**2SO**	0.0036	6.07
**E3**	0.0014	6.63
**5HO**	0.0011	6.77
**1HO**	0.0011	6.77
**5E**	0.0009	6.89
**OE**	0.0004	7.37
**2H3**	0.0004	7.37
**5H4**	0.0002	7.78
**3S1**	0.0001	8.19
**OH1**	0.0001	8.19
**2E**	0.0001	8.19

Mol (**1**) Fuc

**Puck**	**%**	**Δ*G***
**1C4**	99.9951	0.00
**1E**	0.0017	6.51
**1HO**	0.0011	6.77
**EO**	0.0009	6.89
**1H2**	0.0006	7.13
**5HO**	0.0003	7.54
**E2**	0.0002	7.78
**5E**	0.0001	8.19

Mol (**2**) Fuc

**1C4**	99.9959	0.00
**1H2**	0.0015	6.59
**EO**	0.0009	6.89
**1E**	0.0007	7.04
**5HO**	0.0003	7.54
**1HO**	0.0003	7.54
**3OB**	0.0002	7.78
**3S1**	0.0001	8.19
**E2**	0.0001	8.19

Mol (**4**) Fuc

**Puck**	**%**	**Δ*G***
**1C4**	99.9514	0.00
**3S1**	0.0352	4.72
**B14**	0.0040	6.01
**1E**	0.0028	6.22
**1HO**	0.0021	6.39
**3OB**	0.0016	6.55
**1H2**	0.0013	6.67
**5S1**	0.0004	7.37
**E2**	0.0004	7.37
**EO**	0.0003	7.54
**5E**	0.0002	7.78
**3H2**	0.0002	7.78
**5HO**	0.0001	8.19

Table S23. Calculated pyranose puckering populations (%) and relative free energies (Δ*G*, kcal mol^{-1}) from the 10 µs simulations: NeuAc

NB: In this table *θ*=O5-C1-C2-C3-C4-C5 (not O6-C2-C3-C4-C5-C6), therefore the carbon number should be incremented by 1 (*i.e.* $^{1}C_{4}$=$^{2}C_{5}$, $^{5}S_{1}$=$^{6}S_{2}$ etc.)

Mol (**1**) NeuAc			Mol (**2**) NeuAc			Mol (**3**) NeuAc			Mol (**6**) NeuAc		
**Puck**	**%**	**Δ*G***	**Puck**	**%**	**Δ*G***	**Puck**	**%**	**Δ*G***	**Puck**	**%**	**Δ*G***
**1C4**	86.9965	0.08	**1C4**	93.7913	0.04	**1C4**	83.9744	0.10	**1C4**	87.8913	0.08
**5S1**	10.4665	1.34	**5S1**	4.8863	1.79	**5S1**	12.1260	1.25	**3OB**	2.7366	2.13
**B14**	1.5216	2.48	**B14**	0.3887	3.29	**25B**	2.3730	2.22	**3S1**	2.0671	2.30
**25B**	0.3201	3.41	**25B**	0.2434	3.57	**B14**	0.7873	2.87	**OS2**	2.0656	2.30
**2SO**	0.2003	3.68	**1HO**	0.1390	3.90	**2SO**	0.1705	3.78	**5S1**	1.4890	2.49
**EO**	0.1144	4.02	**EO**	0.1382	3.90	**1HO**	0.1240	3.97	**4C1**	1.1169	2.67
**1HO**	0.0957	4.12	**5HO**	0.1260	3.96	**5HO**	0.1182	4.00	**B14**	0.8522	2.83
**5HO**	0.0949	4.13	**2SO**	0.0989	4.10	**EO**	0.1135	4.02	**25B**	0.6225	3.01
**OS2**	0.0382	4.67	**OS2**	0.0776	4.25	**OS2**	0.0945	4.13	**EO**	0.3871	3.29
**5H4**	0.0349	4.72	**5H4**	0.0296	4.82	**5H4**	0.0268	4.88	**2SO**	0.3608	3.34
**E4**	0.0192	5.07	**E4**	0.0194	5.07	**E4**	0.0179	5.12	**5HO**	0.1805	3.75
**B3O**	0.0158	5.19	**1E**	0.0181	5.11	**1E**	0.0148	5.23	**1HO**	0.0488	4.52
**5E**	0.0157	5.19	**5E**	0.0149	5.23	**5E**	0.0145	5.24	**5H4**	0.0372	4.68
**1E**	0.0144	5.25	**3OB**	0.0081	5.59	**3OB**	0.0098	5.47	**5E**	0.0329	4.76
**1S3**	0.0083	5.57	**B3O**	0.0066	5.71	**B3O**	0.0084	5.57	**2H1**	0.0209	5.02
**3S1**	0.0068	5.69	**1H2**	0.0033	6.12	**3S1**	0.0068	5.69	**E4**	0.0164	5.17
**1H2**	0.0068	5.69	**3H4**	0.0029	6.20	**1H2**	0.0039	6.02	**E1**	0.0158	5.19
**E2**	0.0066	5.71	**E2**	0.0017	6.51	**1S3**	0.0032	6.14	**B25**	0.0102	5.45
**4C1**	0.0060	5.76	**1S5**	0.0012	6.72	**1S5**	0.0031	6.16	**B3O**	0.0098	5.47
**3OB**	0.0053	5.84	**3S1**	0.0011	6.77	**B25**	0.0025	6.28	**1E**	0.0087	5.54
**B25**	0.0031	6.16	**3H2**	0.0011	6.77	**3H4**	0.0022	6.36	**2E**	0.0061	5.75
**2H3**	0.0031	6.16	**1S3**	0.0009	6.89	**E2**	0.0019	6.45	**1H2**	0.0046	5.92
**3H4**	0.0019	6.45	**2H3**	0.0006	7.13	**3H2**	0.0012	6.72	**E2**	0.0042	5.98
**3H2**	0.0012	6.72	**14B**	0.0004	7.37	**2H1**	0.0004	7.37	**2H3**	0.0036	6.07
**E3**	0.0009	6.89	**2H1**	0.0003	7.54	**2H3**	0.0004	7.37	**3H4**	0.0035	6.08
**1S5**	0.0007	7.04	**B25**	0.0002	7.78	**E3**	0.0003	7.54	**1S3**	0.0032	6.14
**3E**	0.0005	7.24	**3E**	0.0002	7.78	**E1**	0.0002	7.78	**E3**	0.0010	6.83
**2H1**	0.0003	7.54				**3E**	0.0002	7.78	**3H2**	0.0010	6.83
**4H3**	0.0002	7.78				**14B**	0.0001	8.19	**1S5**	0.0008	6.96
**2E**	0.0001	8.19							**3E**	0.0006	7.13
									**14B**	0.0005	7.24
									**E5**	0.0002	7.78
									**4H3**	0.0002	7.78
									**4E**	0.0001	8.19
									**OH1**	0.0001	8.19

Table S24. Calculated pyranose ring puckering populations (%) and relative free energies (Δ*G*, kcal mol^{-1}) from the 10 μs simulations: Man

Mol (**7**) Man (1→3,1→6)		
**Puck**	**%**	**Δ*G***
**4C1**	99.6457	0.00
**B3O**	0.2304	3.60
**2SO**	0.0398	4.64
**2E**	0.0270	4.87
**2H3**	0.0242	4.94
**2H1**	0.0110	5.41
**1S3**	0.0106	5.43
**E3**	0.0068	5.69
**4H3**	0.0032	6.14
**E1**	0.0006	7.13
**4E**	0.0004	7.37
**25B**	0.0002	7.78
**E5**	0.0001	8.19

Mol (**7**) Man (1→3)		
**Puck**	**%**	**Δ*G***
**4C1**	99.8996	0.00
**B3O**	0.0579	4.42
**2SO**	0.0255	4.91
**2H3**	0.0059	5.77
**2E**	0.0058	5.78
**2H1**	0.0025	6.28
**E3**	0.0010	6.83
**1S3**	0.0005	7.24
**E1**	0.0004	7.37
**4H3**	0.0004	7.37
**25B**	0.0003	7.54
**4E**	0.0002	7.78

Mol (**7**) Man (1→6)		
**Puck**	**%**	**Δ*G***
**4C1**	99.7982	0.00
**B3O**	0.1127	4.03
**2SO**	0.0675	4.33
**2H3**	0.0075	5.63
**2E**	0.0060	5.76
**E3**	0.0032	6.14
**2H1**	0.0021	6.39
**1S3**	0.0015	6.59
**4H3**	0.0008	6.96
**E1**	0.0003	7.54
**4E**	0.0002	7.78

Mol (**8**) Man (1→3,1→6)		
**Puck**	**%**	**Δ*G***
**4C1**	99.5905	0.00
**B3O**	0.2858	3.47
**2SO**	0.0417	4.62
**2E**	0.0285	4.84
**2H3**	0.0233	4.96
**2H1**	0.0129	5.31
**E3**	0.0079	5.60
**1S3**	0.0047	5.91
**4H3**	0.0028	6.22
**4E**	0.0006	7.13
**25B**	0.0005	7.24
**E1**	0.0003	7.54
**5S1**	0.0002	7.78
**4H5**	0.0002	7.78
**OH1**	0.0001	8.19

Mol (**8**) Man (1→3)		
**Puck**	**%**	**Δ*G***
**4C1**	99.4711	0.00
**B3O**	0.3989	3.28
**2SO**	0.0856	4.19
**2H3**	0.0113	5.39
**2E**	0.0112	5.39
**1S3**	0.0074	5.64
**2H1**	0.0055	5.82
**E3**	0.0033	6.12
**5S1**	0.0021	6.39
**4H3**	0.0012	6.72
**E5**	0.0004	7.37
**4H5**	0.0004	7.37
**4E**	0.0004	7.37
**25B**	0.0003	7.54
**E1**	0.0003	7.54
**OH5**	0.0003	7.54
**OH1**	0.0002	7.78
**B14**	0.0001	8.19

Mol (**8**) Man (1→6)		
**Puck**	**%**	**Δ*G***
**4C1**	97.9142	0.01
**B3O**	1.3841	2.54
**2SO**	0.6024	3.03
**1S3**	0.0398	4.64
**2H3**	0.0204	5.04
**E3**	0.0134	5.29
**2E**	0.0115	5.38
**2H1**	0.0048	5.90
**4H3**	0.0030	6.18
**E5**	0.0013	6.67
**4E**	0.0011	6.77
**OH5**	0.0008	6.96
**4H5**	0.0008	6.96
**E1**	0.0007	7.04
**5S1**	0.0005	7.24
**25B**	0.0005	7.24
**OH1**	0.0003	7.54
**OE**	0.0002	7.78
**14B**	0.0001	8.19
**3H4**	0.0001	8.19

Table S25: Calculated and experimental (EXP) NMR three-bond vicinal couplings (Hz): molecules **1-8**

All calculated data for **1-8** are derived from 10 μs simulations (also see Computational Methods).

Key:

- Reference calculated and experimental values not from this work are italicised and underlined
- Mono: data for the free aqueous monosaccharide (calculated or experimental)
- DP3: the trisaccharide NeuAc-α(2→3)-Gal-β(1→4)-Glc
- HA: mean values from NMR for internal GlcNAc rings in a hyaluronan oligosaccharide
- Experimental errors are estimated to be ± 0.2 Hz
- 10 μs simulations were split into 1 μs sub-sets to compute standard deviations (at most ± 0.4 Hz)

A. GlcNAc

	(**1**)	(**2**)	(**3**)	(**4**)	(**5**)	(**8**) 1→3	(**8**) 1→6	***EXP Mono***[2]	***EXP HA***[2]
$J_{1,2}$	7.5	7.5	7.5	7.5	7.5	7.5	7.4	*8.5*	*8.6*
$J_{2,3}$	8.7	8.5	8.7	8.7	8.6	8.5	8.4	*10.4*	*10.3*
$J_{3,4}$	8.0	7.8	8.0	8.0	7.6	7.6	7.5	*8.8*	*8.7*
$J_{4,5}$	8.4	8.4	8.3	8.3	8.2	8.2	8.1	*n/d*	*9.9*

n/d = not determined

B. Gal

	(**1**)	(**2**)	(**3**)	***CAL Mono***[3]	***EXP MonoA***[4]	***EXP MonoB***[5]	***EXP DP3***[5]
$J_{1,2}$	7.9	8.1	7.7	*8.1*	*8.0*	*7.9*	*7.8*
$J_{2,3}$	8.2	8.3	7.8	*8.3*	*10.0*	*9.9*	*9.0*
$J_{3,4}$	2.4	2.3	2.6	*2.5*	*3.8*	*3.5*	*3.1*
$J_{4,5}$	0.7	0.8	1.0	*0.8*	*1.0*	*1.5*	*1.5*

C. NeuAc

	(**1**)	(**2**)	(**3**)	(**6**)	***EXP MonoA***[5]	***EXP MonoB***[6]	***EXP DP3***[5]
$J_{3ax,4}$	9.8	10.4	9.5	10.4	*12.3*	*11.8*	*11.5*
$J_{3eq,4}$	4.9	4.7	4.8	4.7	*5.0*	*5.0*	*4.5*
$J_{4,5}$	8.2	8.4	8.0	8.3	*10.2*	*10.4*	*10.4*
$J_{5,6}$	8.9	8.9	8.9	8.6	*10.3*	*10.7*	*9.9*

Table S25 continued

D. Fuc

	(**1**)	(**2**)	(**4**)	***CAL Mono***[3]	***EXP Mono***[7]
$J_{1,2}$	1.7	1.6	1.7	*3.5*	*4.0*
$J_{2,3}$	8.4	8.4	8.4	*8.4*	*10.0*
$J_{3,4}$	2.2	2.2	2.3	*2.4*	*4.2*
$J_{4,5}$	0.8	0.9	0.8	*0.9*	*0.9*

E. Man

Mol **7**		(**7**) $^{1\to3,\,1\to6}$	(**7**) $^{1\to3}$	(**7**) $^{1\to6}$	***Mono***[3,4]
**CAL**	$J_{1,2}$	2.3	2.4	2.4	*2.3*
**CAL**	$J_{2,3}$	2.6	2.6	2.6	*2.6*
**CAL**	$J_{3,4}$	8.3	8.3	8.3	*8.3*
**CAL**	$J_{4,5}$	8.3	8.2	8.3	*8.2*
**EXP**[1]	$J_{1,2}$	*1.8*	*1.8*	*1.6*	*1.8*
**EXP**[1]	$J_{2,3}$	*3.3*	*3.6*	*3.3*	*3.8*
**EXP**[1]	$J_{3,4}$	*9.5*	*9.5*	*8.8*	*10.0*
**EXP**[1]	$J_{4,5}$	*9.8*	*8.0*	*10.3*	*9.8*

Mol **8**		(**8**) $^{1\to3,\,1\to6}$	(**8**) $^{1\to3}$	(**8**) $^{1\to6}$
**CAL**	$J_{1,2}$	2.3	2.4	2.4
**CAL**	$J_{2,3}$	2.6	2.6	2.6
**CAL**	$J_{3,4}$	8.3	8.3	8.3
**CAL**	$J_{4,5}$	8.3	8.2	8.3

(1) Sayers, E. W.; Prestegard, J. H. *Biophys J* **2000**, *79*, 3313.
(2) Blundell, C. D.; Roberts, I. S.; Sheehan, J. K.; Almond, A. *J Mol Microb Biotech* **2009**, *17*, 71.
(3) Sattelle, B. M.; Almond, A. *Phys. Chem. Chem. Phys.* **2012**, *14*, 5843.
(4) Dowd, M. K.; French, A. D.; Reilly, P. J. *Carbohyd Res* **1994**, *264*, 1.
(5) Lerner, L.; Bax, A. *Carbohydr Res* **1987**, *166*, 35.
(6) Beau, J. M.; Schauer, R.; Haverkamp, J.; Dorland, L.; Vliegenthart, J. F. G.; Sinay, P. *Carbohyd Res* **1980**, *82*, 125.
(7) Otter, A.; Lemieux, R. U.; Ball, R. G.; Venot, A. P.; Hindsgaul, O.; Bundle, D. R. *Eur J Biochem* **1999**, *259*, 295.

S26. Radial distribution functions for through-space interaction of glycosidic bond oxygen atoms and TIP3P water hydrogen atoms around the α(1→3) and β(1→4) linkages in **2**, **3** and **4**.

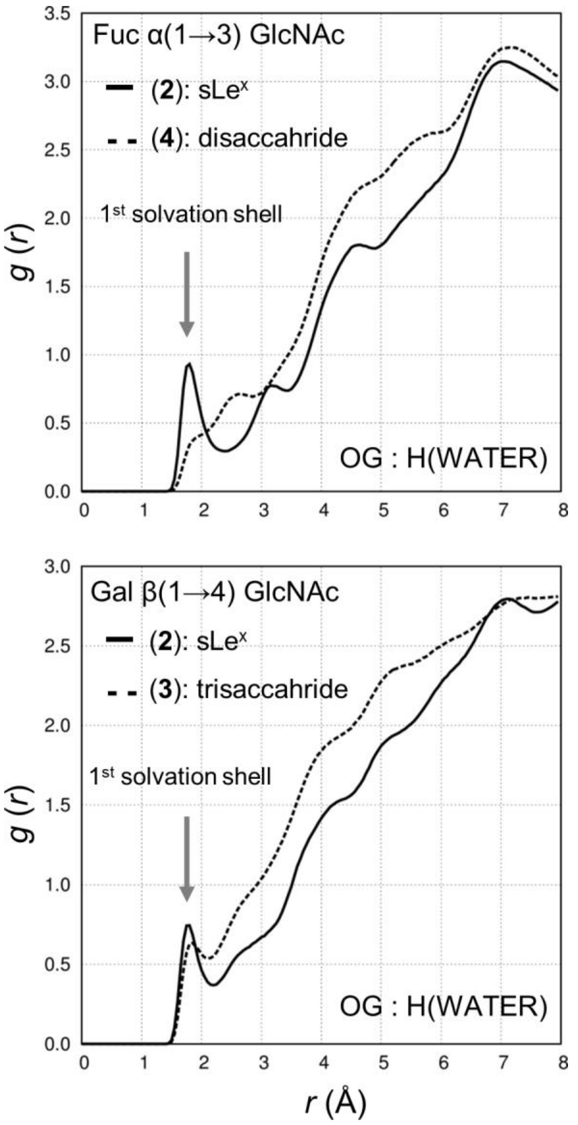

Data are calculated from 10 µs simulations of **1** and **2**.

S27. Calculation of errors in one-dimensional puckering free energy surfaces (Figures 6 and 7)

Errors in computed puckering relative free energies (derived from populations, relative to the lowest energy pucker), were calculated by splitting each 10 µs trajectory into 10 x 1 µs sub-trajectories, in which the 3D-data were randomly selected. The puckering populations and free energies were then calculated using successively more randomly selected data. These analyses showed that using 80% of the randomly selected data (equivalent to an 8 µs trajectory of random 3D-data) gave extremely small errors in computed relative free energies (±0.01 kcal mol^{-1}) for puckers less than or equal to 5 kcal mol^{-1} above the lowest energy pucker. At 7 kcal mol^{-1} above the lowest energy pucker the error was ±0.2 kcal mol^{-1} and the largest calculated errors were ±0.6 kcal mol^{-1}, which were evident at > 9 kcal mol^{-1} above the lowest energy pucker.

S28: Calculation of nuclear Overhauser effects (nOes) from 800 MHz NMR of molecules **1** and **2**

Four conformationally independent reference nOe intensities (from cross-peaks of protons in fixed geometric arrangement: Fuc:H1-H2, Fuc:H4-H5, Gal:H1-H3 and Gal:H1-H5) were used to parameterise a model relating cross-peak intensity (I) to proton-proton distance (r) using the relationship $I = \frac{k}{r^6}$. The constant of proportionality (k) was then derived from a linear plot of $\frac{1}{r^6}$ and intensity (I) for the reference nOes.

<u>Standard (*Std*) and back-predicted (*Pred*) distances (Å) for calibration / reference nOes</u>

	Molecule **1**					Molecule **2**				
<u>nOe</u>	<u>*Std**</u>	<u>*Pred*</u>	<u>*Std-Pred*</u>	<u>*S^2*</u>	<u>*nOe intensity*</u>	<u>*Std**</u>	<u>*Pred*</u>	<u>*Std-Pred*</u>	<u>*S^2*</u>	<u>*Intensity*</u>
Fuc:H1-H2	2.4	1.9	0.5	0.87	*27116350*	2.4	2.2	0.2	0.76	*31682622*
Fuc:H4-H5	2.5	2.0	0.5	0.86	*20723074*	2.5	2.8	-0.3	0.75	*6777185*
Gal:H1-H3	3.1	2.0	1.1	0.96	*20263030*	2.9	2.5	0.4	0.85	*15013054*
Gal:H1-H5	2.5	2.0	0.5	0.89	*20543788*	2.5	2.3	0.2	0.76	*21058458*
STDEV			0.3			0.3				

**Standard (Std) distances were measured from initial (energy minimised) starting structures*

Order parameters (S^2) calculated from 10 µs-dynamics (see S31); smaller value indicates greater mobility

<u>Distances (Å) for an inter-ring nOe (± STDEV)</u>

- <u>derived from NMR (*Pred_NMR*) and 10 µs-dynamics (simulation average, *Pred_MD*)</u>

Molecule **1**

<u>nOe</u>	<u>*Pred_MD*</u>	<u>*Pred_NMR*</u>	<u>*S^2*</u>
Gal:H2-Fuc:H5	2.5 (±0.4)	2.1 (±0.3)	0.84

Molecule **2**

<u>nOe</u>	<u>*Pred_MD*</u>	<u>*Pred_NMR*</u>	<u>*S^2*</u>
Gal:H2-Fuc:H5	2.6 (±0.8)	2.6 (±0.3)	0.90

Order parameters (S^2) calculated from 10 µs-dynamics (see S31); smaller value indicates greater mobility

Table S29. Assigned ^{1}H and ^{13}C chemical shifts in molecules **1** and **2** (800 MHz NMR spectroscopy)

		**Mol (1)**		**Mol (2)**	
	Atom	^{13}C	^{1}H	^{13}C	^{1}H
**Fuc**	1	98.05	5.01	98.59	5.09
**Fuc**	2	67.82	3.79	67.71	3.67
**Fuc**	3	69.12	3.88	69.21	3.89
**Fuc**	4	71.95	3.78	71.90	3.77
**Fuc**	5	66.83	4.88	66.65	4.82
**Fuc**	Me	15.34	1.17	15.24	1.16
**Gal**	1	102.84	4.54	101.62	4.52
**Gal**	2	68.79	3.50	69.25	3.53
**Gal**	3	75.64	4.05	75.64	4.08
**Gal**	4	66.90	3.91	67.29	3.93
**Gal**	5	74.65	3.53	74.92	3.58
**Gal**	6	61.63	3.69	61.52	3.69
**NeuAc**	3	40.04	1.76	39.79	1.79
**NeuAc**	3	40.04	2.77	39.79	2.76
**NeuAc**	4	68.43	3.67	68.24	3.68
**NeuAc**	5	51.65	3.84	51.69	3.84
**NeuAc**	6	72.73	3.62	72.90	3.65
**NeuAc**	7	68.02	3.61	68.12	3.59
**NeuAc**	8	71.86	3.85	71.86	3.89
**NeuAc**	9	62.27	3.82	62.60	3.87
**NeuAc**	9	62.27	3.65	62.60	3.64
**NeuAc**	Me	22.08	2.04	21.88	2.02
**α-GlcN**	1	94.77	4.71	94.77	4.72
**α-GlcN**	2	56.84	3.85	56.92	3.87
**α-GlcN**	3	76.02	4.06	74.84	3.84
**α-GlcN**	4	72.32	3.74	73.29	3.95
**α-GlcN**	5	75.59	3.55	75.37	3.59
**α-GlcN**	6	59.72	3.95	59.65	3.97
**α-GlcN**	6	59.72	3.87	59.65	3.91
**α-GlcN**	Me	22.16	2.03	22.10	2.02
**β-GlcN**	1	91.00	5.11	91.11	5.09
**β-GlcN**	2	54.00	4.14	54.12	4.15
**β-GlcN**	3	74.13	4.17	72.74	4.00
**β-GlcN**	4	72.37	3.75	73.29	3.95
**β-GlcN**	5	71.43	3.98	71.18	4.01
**β-GlcN**	6	59.74	3.95	59.58	3.93
**β-GlcN**	6	59.68	3.87	59.59	3.89
**β-GlcN**	Me	22.16	2.03	22.10	2.03

Figure S30. Example nOes evidencing Gal and Fuc ring stacking in **1** and **2** from 800 MHz NMR

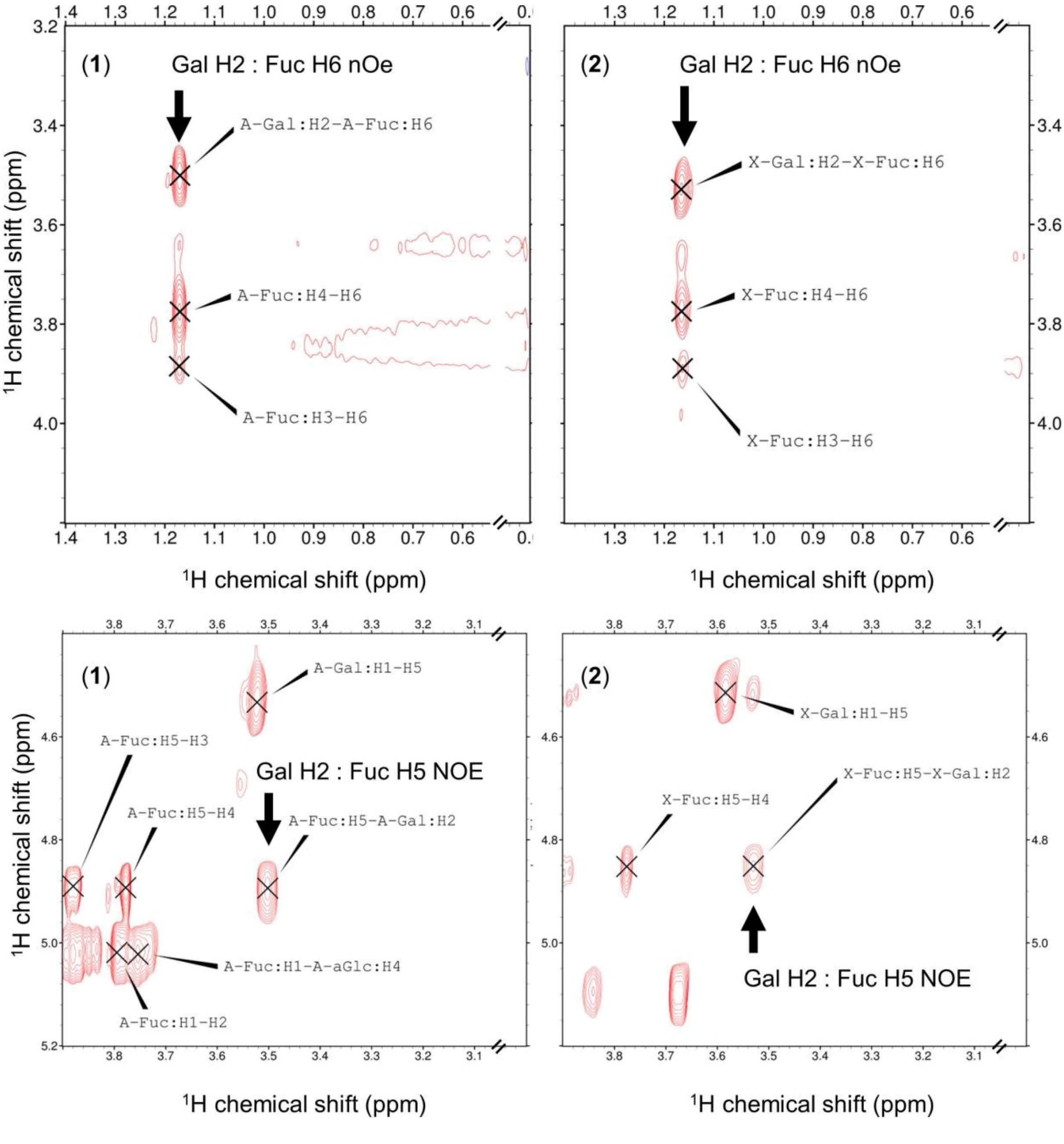

Table S31. Calculation of order parameters

Order parameters (S^2, tabulated in S28) were derived using the Amber tool ptraj and the method of Prompers & Brüschweiler (JACS 2002, 124, 4522). A step and correlation time of 10 ps and 10 ns were employed. The following double exponential function was used to fit the time correlation curve and extract S^2 (as parameter c, where x=time).

$$f(x) = c+a*exp(-t/k1)+b*exp(-t/k2)$$

Autocorrelation function and the fitting function f(t) for the Gal-H2LFuc-H5 bond vector in molecule **1**.

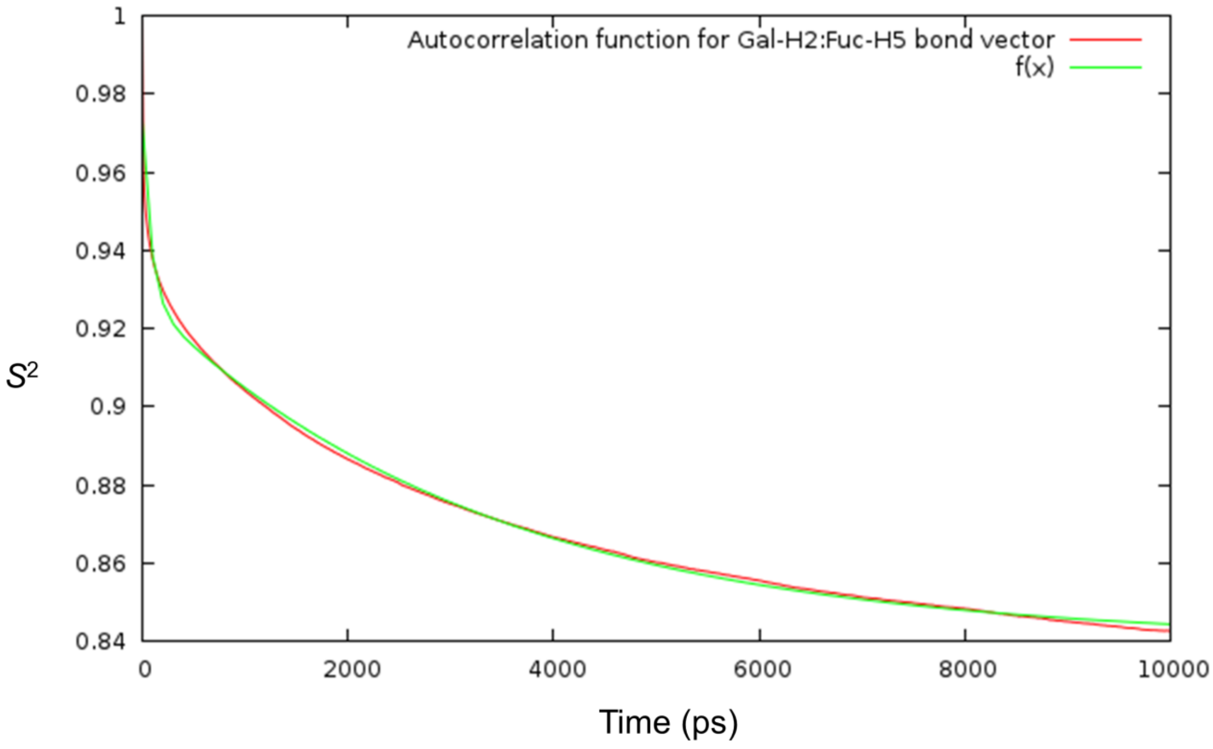

Figure S32: Cartesian coordinates of most populated conformer (GLYCAM naming convention): **1**

Derived from the 10 μs simulation

ATOM	1	HO1	ROH	X	1	2.453	-21.454	0.987
ATOM	2	O1	ROH	X	1	1.664	-21.529	0.446
ATOM	3	C1	WYB	X	2	1.638	-20.402	-0.437
ATOM	4	H1	WYB	X	2	1.990	-20.652	-1.438
ATOM	5	O5	WYB	X	2	2.476	-19.490	0.258
ATOM	6	C5	WYB	X	2	2.710	-18.257	-0.528
ATOM	7	H5	WYB	X	2	3.112	-18.466	-1.520
ATOM	8	C6	WYB	X	2	3.747	-17.455	0.347
ATOM	9	H62	WYB	X	2	3.997	-16.576	-0.247
ATOM	10	H61	WYB	X	2	4.657	-18.045	0.461
ATOM	11	O6	WYB	X	2	3.118	-17.009	1.519
ATOM	12	H6O	WYB	X	2	3.771	-16.429	1.918
ATOM	13	C4	WYB	X	2	1.305	-17.513	-0.686
ATOM	14	H4	WYB	X	2	0.830	-17.227	0.253
ATOM	15	O4	WYB	X	2	1.663	-16.392	-1.475
ATOM	16	C3	WYB	X	2	0.393	-18.486	-1.473
ATOM	17	H3	WYB	X	2	0.836	-18.577	-2.465
ATOM	18	C2	WYB	X	2	0.266	-19.804	-0.713
ATOM	19	H2	WYB	X	2	-0.264	-19.732	0.236
ATOM	20	N2	WYB	X	2	-0.494	-20.771	-1.588
ATOM	21	H2N	WYB	X	2	0.012	-21.370	-2.226
ATOM	22	C2N	WYB	X	2	-1.788	-20.994	-1.381
ATOM	23	O2N	WYB	X	2	-2.409	-20.295	-0.565
ATOM	24	CME	WYB	X	2	-2.514	-22.125	-2.092
ATOM	25	H3M	WYB	X	2	-3.512	-22.202	-1.662
ATOM	26	H2M	WYB	X	2	-2.649	-22.029	-3.170
ATOM	27	H1M	WYB	X	2	-1.956	-22.999	-1.757
ATOM	28	O3	WYB	X	2	-0.892	-17.836	-1.511
ATOM	29	C1	3LB	X	3	-1.558	-17.765	-2.815
ATOM	30	H1	3LB	X	3	-1.645	-18.773	-3.223
ATOM	31	O5	3LB	X	3	-0.711	-16.953	-3.728
ATOM	32	C5	3LB	X	3	-1.289	-16.773	-5.058
ATOM	33	H5	3LB	X	3	-1.359	-17.759	-5.516
ATOM	34	C6	3LB	X	3	-0.418	-15.964	-5.977
ATOM	35	H62	3LB	X	3	-0.040	-15.096	-5.437
ATOM	36	H61	3LB	X	3	-0.987	-15.766	-6.885
ATOM	37	O6	3LB	X	3	0.678	-16.867	-6.265
ATOM	38	H6O	3LB	X	3	1.398	-16.606	-5.687
ATOM	39	C4	3LB	X	3	-2.659	-16.153	-4.958
ATOM	40	H4	3LB	X	3	-3.055	-16.072	-5.971
ATOM	41	O4	3LB	X	3	-2.431	-14.843	-4.448
ATOM	42	H4O	3LB	X	3	-3.319	-14.481	-4.389
ATOM	43	C3	3LB	X	3	-3.617	-16.892	-4.080
ATOM	44	H3	3LB	X	3	-3.853	-17.895	-4.436
ATOM	45	C2	3LB	X	3	-2.943	-17.134	-2.694
ATOM	46	H2	3LB	X	3	-2.851	-16.128	-2.287
ATOM	47	O2	3LB	X	3	-3.804	-17.942	-1.868
ATOM	48	H2O	3LB	X	3	-3.889	-18.790	-2.311
ATOM	49	O3	3LB	X	3	-4.900	-16.262	-3.827
ATOM	50	C2	0SA	X	4	-5.940	-16.560	-4.683
ATOM	51	C1	0SA	X	4	-5.677	-15.980	-6.180
ATOM	52	O1A	0SA	X	4	-5.530	-14.770	-6.340
ATOM	53	O1B	0SA	X	4	-5.586	-16.842	-7.109
ATOM	54	C3	0SA	X	4	-7.139	-15.877	-4.065
ATOM	55	H3E	0SA	X	4	-7.061	-14.791	-4.111
ATOM	56	H3A	0SA	X	4	-7.151	-16.104	-2.999
ATOM	57	C4	0SA	X	4	-8.372	-16.442	-4.823
ATOM	58	H4	0SA	X	4	-8.267	-16.185	-5.876
ATOM	59	C5	0SA	X	4	-8.533	-17.922	-4.711
ATOM	60	H5	0SA	X	4	-8.726	-18.104	-3.654
ATOM	61	C6	0SA	X	4	-7.292	-18.575	-5.293
ATOM	62	H6	0SA	X	4	-7.253	-18.345	-6.358
ATOM	63	C7	0SA	X	4	-7.280	-20.106	-5.109
ATOM	64	H7	0SA	X	4	-8.223	-20.465	-5.520
ATOM	65	C8	0SA	X	4	-5.999	-20.712	-5.778
ATOM	66	H8	0SA	X	4	-5.050	-20.298	-5.437
ATOM	67	C9	0SA	X	4	-5.830	-22.156	-5.731

ATOM	68	H9S	0SA	X	4	-6.865	-22.465	-5.585
ATOM	69	H9R	0SA	X	4	-5.493	-22.493	-6.712
ATOM	70	O9	0SA	X	4	-5.065	-22.592	-4.600
ATOM	71	H9O	0SA	X	4	-5.236	-23.531	-4.506
ATOM	72	O8	0SA	X	4	-6.084	-20.420	-7.163
ATOM	73	H8O	0SA	X	4	-5.274	-19.928	-7.311
ATOM	74	O7	0SA	X	4	-7.405	-20.385	-3.674
ATOM	75	H7O	0SA	X	4	-7.898	-21.169	-3.421
ATOM	76	O6	0SA	X	4	-6.204	-17.989	-4.620
ATOM	77	N5	0SA	X	4	-9.717	-18.312	-5.517
ATOM	78	H5N	0SA	X	4	-9.691	-18.190	-6.519
ATOM	79	C5N	0SA	X	4	-10.658	-19.109	-5.026
ATOM	80	CME	0SA	X	4	-11.887	-19.307	-5.928
ATOM	81	H3M	0SA	X	4	-11.721	-20.194	-6.538
ATOM	82	H2M	0SA	X	4	-11.851	-18.471	-6.626
ATOM	83	H1M	0SA	X	4	-12.848	-19.346	-5.415
ATOM	84	O5N	0SA	X	4	-10.594	-19.558	-3.879
ATOM	85	O4	0SA	X	4	-9.591	-15.728	-4.276
ATOM	86	H4O	0SA	X	4	-10.197	-15.839	-5.012
ATOM	87	C1	0fA	X	5	1.630	-15.073	-0.868
ATOM	88	H1	0fA	X	5	2.310	-15.185	-0.024
ATOM	89	O5	0fA	X	5	0.268	-14.766	-0.416
ATOM	90	C5	0fA	X	5	-0.735	-14.538	-1.550
ATOM	91	H5	0fA	X	5	-0.818	-15.437	-2.161
ATOM	92	C6	0fA	X	5	-2.060	-14.287	-0.850
ATOM	93	H61	0fA	X	5	-2.813	-14.032	-1.595
ATOM	94	H62	0fA	X	5	-2.010	-13.515	-0.082
ATOM	95	H63	0fA	X	5	-2.484	-15.119	-0.288
ATOM	96	C4	0fA	X	5	-0.172	-13.443	-2.370
ATOM	97	H4	0fA	X	5	-0.917	-13.158	-3.114
ATOM	98	O4	0fA	X	5	0.081	-12.217	-1.486
ATOM	99	H4O	0fA	X	5	-0.758	-11.759	-1.394
ATOM	100	C3	0fA	X	5	1.147	-13.891	-3.003
ATOM	101	H3	0fA	X	5	1.070	-14.848	-3.521
ATOM	102	O3	0fA	X	5	1.501	-12.863	-3.973
ATOM	103	H3O	0fA	X	5	2.417	-13.042	-4.200
ATOM	104	C2	0fA	X	5	2.150	-14.079	-1.906
ATOM	105	H2	0fA	X	5	2.393	-13.104	-1.483
ATOM	106	O2	0fA	X	5	3.286	-14.535	-2.560
ATOM	107	H2O	0fA	X	5	3.937	-13.910	-2.232

Figure S33: Cartesian coordinates of most populated conformer (GLYCAM naming convention): **2**

Derived from the 25 μs simulation

ATOM	1	HO1	ROH	X	1	-22.801	-7.834	11.511	0	0
ATOM	2	O1	ROH	X	1	-23.416	-7.107	11.633	0	0
ATOM	3	C1	WYB	X	2	-22.903	-6.059	10.816	0	0
ATOM	4	H1	WYB	X	2	-23.772	-5.769	10.224	0	0
ATOM	5	O5	WYB	X	2	-21.879	-6.644	10.035	0	0
ATOM	6	C5	WYB	X	2	-21.135	-5.712	9.145	0	0
ATOM	7	H5	WYB	X	2	-21.781	-5.101	8.513	0	0
ATOM	8	C6	WYB	X	2	-20.133	-6.534	8.275	0	0
ATOM	9	H62	WYB	X	2	-19.822	-5.912	7.435	0	0
ATOM	10	H61	WYB	X	2	-20.656	-7.403	7.873	0	0
ATOM	11	O6	WYB	X	2	-19.038	-7.095	9.012	0	0
ATOM	12	H6O	WYB	X	2	-18.99	-8.048	8.902	0	0
ATOM	13	C4	WYB	X	2	-20.411	-4.709	9.963	0	0
ATOM	14	H4	WYB	X	2	-19.817	-5.259	10.692	0	0
ATOM	15	O4	WYB	X	2	-19.66	-3.81	9.083	0	0
ATOM	16	C3	WYB	X	2	-21.395	-3.846	10.754	0	0
ATOM	17	H3	WYB	X	2	-21.998	-3.26	10.061	0	0
ATOM	18	C2	WYB	X	2	-22.221	-4.838	11.588	0	0
ATOM	19	H2	WYB	X	2	-21.519	-5.214	12.332	0	0
ATOM	20	N2	WYB	X	2	-23.219	-4.153	12.369	0	0
ATOM	21	H2N	WYB	X	2	-24.009	-3.852	11.817	0	0
ATOM	22	C2N	WYB	X	2	-23.306	-3.949	13.711	0	0
ATOM	23	O2N	WYB	X	2	-22.382	-4.374	14.382	0	0
ATOM	24	CME	WYB	X	2	-24.487	-3.422	14.209	0	0
ATOM	25	H3M	WYB	X	2	-24.359	-3.123	15.25	0	0
ATOM	26	H2M	WYB	X	2	-24.619	-2.427	13.782	0	0
ATOM	27	H1M	WYB	X	2	-25.298	-4.1	13.941	0	0
ATOM	28	O3	WYB	X	2	-20.633	-2.872	11.557	0	0
ATOM	29	C1	3LB	X	3	-18.21	-3.793	9.162	0	0
ATOM	30	H1	3LB	X	3	-17.866	-4.825	9.227	0	0
ATOM	31	O5	3LB	X	3	-17.759	-3.106	10.324	0	0
ATOM	32	C5	3LB	X	3	-16.306	-2.968	10.519	0	0
ATOM	33	H5	3LB	X	3	-15.902	-3.975	10.417	0	0
ATOM	34	C6	3LB	X	3	-16.049	-2.515	11.967	0	0
ATOM	35	H62	3LB	X	3	-16.88	-1.896	12.304	0	0
ATOM	36	H61	3LB	X	3	-15.077	-2.033	12.072	0	0
ATOM	37	O6	3LB	X	3	-16.053	-3.755	12.83	0	0
ATOM	38	H6O	3LB	X	3	-15.483	-4.42	12.438	0	0
ATOM	39	C4	3LB	X	3	-15.855	-1.928	9.416	0	0
ATOM	40	H4	3LB	X	3	-14.781	-1.781	9.528	0	0
ATOM	41	O4	3LB	X	3	-16.466	-0.671	9.628	0	0
ATOM	42	H4O	3LB	X	3	-16.032	-0.118	8.974	0	0
ATOM	43	C3	3LB	X	3	-16.206	-2.566	8.041	0	0
ATOM	44	H3	3LB	X	3	-15.487	-3.364	7.859	0	0
ATOM	45	C2	3LB	X	3	-17.661	-3.028	7.888	0	0
ATOM	46	H2	3LB	X	3	-18.374	-2.215	7.75	0	0
ATOM	47	O2	3LB	X	3	-17.698	-3.893	6.772	0	0
ATOM	48	H2O	3LB	X	3	-17.457	-3.386	5.993	0	0
ATOM	49	O3	3LB	X	3	-15.963	-1.52	7.023	0	0
ATOM	50	C2	0SA	X	4	-14.967	-1.906	6.09	0	0
ATOM	51	C1	0SA	X	4	-13.45	-1.837	6.721	0	0
ATOM	52	O1A	0SA	X	4	-12.645	-2.79	6.561	0	0
ATOM	53	O1B	0SA	X	4	-13.138	-0.88	7.458	0	0
ATOM	54	C3	0SA	X	4	-15.143	-0.719	5.164	0	0
ATOM	55	H3E	0SA	X	4	-14.736	0.219	5.542	0	0
ATOM	56	H3A	0SA	X	4	-16.206	-0.513	5.048	0	0
ATOM	57	C4	0SA	X	4	-14.532	-1.043	3.793	0	0
ATOM	58	H4	0SA	X	4	-13.455	-1.062	3.96	0	0
ATOM	59	C5	0SA	X	4	-15	-2.436	3.226	0	0
ATOM	60	H5	0SA	X	4	-16.08	-2.37	3.087	0	0
ATOM	61	C6	0SA	X	4	-14.722	-3.559	4.192	0	0
ATOM	62	H6	0SA	X	4	-13.637	-3.639	4.248	0	0
ATOM	63	C7	0SA	X	4	-15.271	-4.912	3.855	0	0
ATOM	64	H7	0SA	X	4	-14.962	-5.057	2.82	0	0
ATOM	65	C8	0SA	X	4	-14.685	-5.994	4.753	0	0
ATOM	66	H8	0SA	X	4	-14.977	-5.818	5.788	0	0
ATOM	67	C9	0SA	X	4	-15.056	-7.424	4.306	0	0

ATOM	68	H9S	0SA	X	4	-16.143	-7.5	4.283	0	0
ATOM	69	H9R	0SA	X	4	-14.609	-7.588	3.325	0	0
ATOM	70	O9	0SA	X	4	-14.517	-8.386	5.188	0	0
ATOM	71	H9O	0SA	X	4	-14.987	-9.223	5.154	0	0
ATOM	72	O8	0SA	X	4	-13.273	-5.961	4.626	0	0
ATOM	73	H8O	0SA	X	4	-12.936	-6.488	5.354	0	0
ATOM	74	O7	0SA	X	4	-16.71	-4.854	3.827	0	0
ATOM	75	H7O	0SA	X	4	-16.911	-3.937	4.027	0	0
ATOM	76	O6	0SA	X	4	-15.269	-3.162	5.49	0	0
ATOM	77	N5	0SA	X	4	-14.322	-2.65	1.884	0	0
ATOM	78	H5N	0SA	X	4	-13.337	-2.432	1.837	0	0
ATOM	79	C5N	0SA	X	4	-15.018	-3.198	0.928	0	0
ATOM	80	CME	0SA	X	4	-14.26	-3.296	-0.426	0	0
ATOM	81	H3M	0SA	X	4	-13.3	-3.809	-0.361	0	0
ATOM	82	H2M	0SA	X	4	-14.141	-2.298	-0.848	0	0
ATOM	83	H1M	0SA	X	4	-14.872	-3.78	-1.186	0	0
ATOM	84	O5N	0SA	X	4	-16.183	-3.592	0.996	0	0
ATOM	85	O4	0SA	X	4	-14.797	0.116	2.972	0	0
ATOM	86	H4O	0SA	X	4	-14.531	0.904	3.452	0	0
ATOM	87	C1	0fA	X	5	-21.211	-1.564	11.766	0	0
ATOM	88	H1	0fA	X	5	-22.269	-1.695	11.994	0	0
ATOM	89	O5	0fA	X	5	-21.139	-0.76	10.564	0	0
ATOM	90	C5	0fA	X	5	-19.754	-0.147	10.406	0	0
ATOM	91	H5	0fA	X	5	-19.141	-1.034	10.244	0	0
ATOM	92	C6	0fA	X	5	-19.685	0.541	9.018	0	0
ATOM	93	H61	0fA	X	5	-18.778	0.179	8.533	0	0
ATOM	94	H62	0fA	X	5	-19.696	1.629	8.967	0	0
ATOM	95	H63	0fA	X	5	-20.572	0.35	8.413	0	0
ATOM	96	C4	0fA	X	5	-19.321	0.619	11.635	0	0
ATOM	97	H4	0fA	X	5	-18.377	1.102	11.381	0	0
ATOM	98	O4	0fA	X	5	-20.321	1.607	11.912	0	0
ATOM	99	H4O	0fA	X	5	-20.172	2.333	11.302	0	0
ATOM	100	C3	0fA	X	5	-19.331	-0.36	12.806	0	0
ATOM	101	H3	0fA	X	5	-18.772	-1.239	12.486	0	0
ATOM	102	O3	0fA	X	5	-18.729	0.231	13.961	0	0
ATOM	103	H3O	0fA	X	5	-19.049	1.121	14.126	0	0
ATOM	104	C2	0fA	X	5	-20.766	-0.837	13.072	0	0
ATOM	105	H2	0fA	X	5	-21.409	0.015	13.294	0	0
ATOM	106	O2	0fA	X	5	-20.597	-1.811	14.152	0	0
ATOM	107	H2O	0fA	X	5	-19.691	-2.13	14.141	0	0

Figure S34: Cartesian coordinates of most populated conformer (GLYCAM naming convention): **7**

Derived from the 10 μs simulation

ATOM	1	HO1	ROH	X	1	6.960	-11.486	11.865
ATOM	2	O1	ROH	X	1	6.146	-11.519	11.358
ATOM	3	C1	VMA	X	2	5.726	-10.257	11.411
ATOM	4	H1	VMA	X	2	5.204	-10.046	12.345
ATOM	5	C2	VMA	X	2	4.773	-10.083	10.192
ATOM	6	H2	VMA	X	2	4.125	-10.959	10.152
ATOM	7	O2	VMA	X	2	3.898	-8.920	10.424
ATOM	8	H2O	VMA	X	2	3.274	-8.788	9.706
ATOM	9	C3	VMA	X	2	5.509	-10.075	8.839
ATOM	10	H3	VMA	X	2	5.779	-11.125	8.730
ATOM	11	C4	VMA	X	2	6.722	-9.149	8.888
ATOM	12	H4	VMA	X	2	6.315	-8.138	8.892
ATOM	13	C5	VMA	X	2	7.688	-9.485	10.042
ATOM	14	H5	VMA	X	2	8.135	-10.470	9.907
ATOM	15	C6	VMA	X	2	8.820	-8.421	10.243
ATOM	16	H62	VMA	X	2	9.420	-8.592	11.137
ATOM	17	H61	VMA	X	2	9.546	-8.508	9.435
ATOM	18	O6	VMA	X	2	8.260	-7.089	10.229
ATOM	19	O5	VMA	X	2	6.858	-9.422	11.281
ATOM	20	O4	VMA	X	2	7.413	-9.240	7.652
ATOM	21	H4O	VMA	X	2	7.691	-10.153	7.552
ATOM	22	O3	VMA	X	2	4.622	-9.742	7.764
ATOM	23	C1	0MA	X	3	4.312	-10.873	6.935
ATOM	24	H1	0MA	X	3	5.245	-11.361	6.651
ATOM	25	C2	0MA	X	3	3.552	-10.350	5.672
ATOM	26	H2	0MA	X	3	4.086	-9.555	5.154
ATOM	27	C3	0MA	X	3	2.202	-9.853	6.090
ATOM	28	H3	0MA	X	3	2.369	-9.002	6.751
ATOM	29	C4	0MA	X	3	1.318	-10.852	6.883
ATOM	30	H4	0MA	X	3	1.101	-11.653	6.175
ATOM	31	C5	0MA	X	3	2.123	-11.308	8.043
ATOM	32	H5	0MA	X	3	2.162	-10.488	8.761
ATOM	33	C6	0MA	X	3	1.279	-12.551	8.609
ATOM	34	H62	0MA	X	3	0.240	-12.267	8.777
ATOM	35	H61	0MA	X	3	1.357	-13.268	7.792
ATOM	36	O6	0MA	X	3	1.720	-13.120	9.850
ATOM	37	H6O	0MA	X	3	2.034	-14.001	9.634
ATOM	38	O5	0MA	X	3	3.458	-11.782	7.784
ATOM	39	O4	0MA	X	3	0.202	-10.190	7.465
ATOM	40	H4O	0MA	X	3	-0.643	-10.516	7.146
ATOM	41	O3	0MA	X	3	1.496	-9.292	4.956
ATOM	42	H3O	0MA	X	3	1.864	-8.417	4.813
ATOM	43	O2	0MA	X	3	3.436	-11.434	4.754
ATOM	44	H2O	0MA	X	3	3.031	-11.076	3.961
ATOM	45	C1	0MA	X	4	9.362	-6.171	9.856
ATOM	46	H1	0MA	X	4	10.256	-6.505	10.384
ATOM	47	C2	0MA	X	4	8.767	-4.747	10.282
ATOM	48	H2	0MA	X	4	8.491	-4.814	11.334
ATOM	49	C3	0MA	X	4	7.496	-4.410	9.444
ATOM	50	H3	0MA	X	4	6.798	-5.234	9.595
ATOM	51	C4	0MA	X	4	7.879	-4.479	7.911
ATOM	52	H4	0MA	X	4	8.668	-3.801	7.583
ATOM	53	C5	0MA	X	4	8.327	-5.919	7.608
ATOM	54	H5	0MA	X	4	7.546	-6.646	7.834
ATOM	55	C6	0MA	X	4	8.731	-6.151	6.092
ATOM	56	H62	0MA	X	4	9.033	-7.155	5.790
ATOM	57	H61	0MA	X	4	7.840	-5.891	5.521
ATOM	58	O6	0MA	X	4	9.889	-5.304	5.760
ATOM	59	H6O	0MA	X	4	9.749	-5.074	4.839
ATOM	60	O5	0MA	X	4	9.533	-6.265	8.388
ATOM	61	O4	0MA	X	4	6.642	-4.253	7.220
ATOM	62	H4O	0MA	X	4	6.472	-3.316	7.095
ATOM	63	O3	0MA	X	4	6.923	-3.162	9.907
ATOM	64	H3O	0MA	X	4	5.989	-3.235	9.696
ATOM	65	O2	0MA	X	4	9.787	-3.714	10.213

ATOM	66	H2O	0MA	X	4	9.621	-3.113	10.943

Figure S35: Cartesian coordinates of most populated conformer (GLYCAM naming convention): **8**

Derived from the 10 μs simulation

ATOM	1	HO1	ROH	X	1	5.944	11.587	-12.385
ATOM	2	O1	ROH	X	1	5.196	11.245	-12.879
ATOM	3	C1	VMA	X	2	5.144	11.793	-14.157
ATOM	4	H1	VMA	X	2	5.462	12.834	-14.098
ATOM	5	C2	VMA	X	2	3.702	11.832	-14.694
ATOM	6	H2	VMA	X	2	3.006	12.277	-13.983
ATOM	7	O2	VMA	X	2	3.693	12.603	-15.914
ATOM	8	H2O	VMA	X	2	4.046	13.476	-15.731
ATOM	9	C3	VMA	X	2	3.115	10.505	-14.922
ATOM	10	H3	VMA	X	2	3.003	9.997	-13.964
ATOM	11	C4	VMA	X	2	3.977	9.640	-15.759
ATOM	12	H4	VMA	X	2	4.062	10.004	-16.783
ATOM	13	C5	VMA	X	2	5.418	9.542	-15.344
ATOM	14	H5	VMA	X	2	5.470	8.918	-14.451
ATOM	15	C6	VMA	X	2	6.292	8.890	-16.439
ATOM	16	H62	VMA	X	2	7.357	8.844	-16.213
ATOM	17	H61	VMA	X	2	6.054	7.832	-16.546
ATOM	18	O6	VMA	X	2	6.107	9.499	-17.792
ATOM	19	O5	VMA	X	2	5.931	10.960	-15.042
ATOM	20	O4	VMA	X	2	3.437	8.285	-15.845
ATOM	21	H4O	VMA	X	2	2.748	8.314	-16.512
ATOM	22	O3	VMA	X	2	1.660	10.534	-15.512
ATOM	23	C1	2MA	X	3	0.705	9.946	-14.596
ATOM	24	H1	2MA	X	3	0.979	9.037	-14.059
ATOM	25	C2	2MA	X	3	-0.554	9.655	-15.336
ATOM	26	H2	2MA	X	3	-0.362	9.096	-16.251
ATOM	27	C3	2MA	X	3	-1.223	10.965	-15.711
ATOM	28	H3	2MA	X	3	-0.662	11.564	-16.430
ATOM	29	C4	2MA	X	3	-1.339	11.845	-14.527
ATOM	30	H4	2MA	X	3	-1.980	11.326	-13.814
ATOM	31	C5	2MA	X	3	0.004	12.278	-13.998
ATOM	32	H5	2MA	X	3	0.542	12.661	-14.865
ATOM	33	C6	2MA	X	3	-0.101	13.339	-12.898
ATOM	34	H62	2MA	X	3	-0.388	14.297	-13.330
ATOM	35	H61	2MA	X	3	-0.826	13.069	-12.130
ATOM	36	O6	2MA	X	3	1.197	13.491	-12.277
ATOM	37	H6O	2MA	X	3	1.496	12.669	-11.883
ATOM	38	O5	2MA	X	3	0.625	11.016	-13.633
ATOM	39	O4	2MA	X	3	-2.068	13.058	-15.009
ATOM	40	H4O	2MA	X	3	-2.998	12.890	-15.175
ATOM	41	O3	2MA	X	3	-2.558	10.579	-16.307
ATOM	42	H3O	2MA	X	3	-3.044	10.088	-15.640
ATOM	43	O2	2MA	X	3	-1.494	8.923	-14.484
ATOM	44	C1	0YB	X	4	-1.241	7.567	-14.259
ATOM	45	H1	0YB	X	4	-0.167	7.516	-14.428
ATOM	46	O5	0YB	X	4	-1.996	6.696	-15.082
ATOM	47	C5	0YB	X	4	-1.776	5.291	-14.735
ATOM	48	H5	0YB	X	4	-0.702	5.148	-14.843
ATOM	49	C6	0YB	X	4	-2.617	4.340	-15.605
ATOM	50	H62	0YB	X	4	-2.566	3.360	-15.129
ATOM	51	H61	0YB	X	4	-2.232	4.235	-16.620
ATOM	52	O6	0YB	X	4	-4.044	4.805	-15.715
ATOM	53	H6O	0YB	X	4	-4.592	4.518	-14.981
ATOM	54	C4	0YB	X	4	-2.149	5.075	-13.233
ATOM	55	H4	0YB	X	4	-3.215	5.165	-13.024
ATOM	56	O4	0YB	X	4	-1.748	3.718	-13.021
ATOM	57	H4O	0YB	X	4	-2.128	3.452	-12.180
ATOM	58	C3	0YB	X	4	-1.347	6.005	-12.271
ATOM	59	H3	0YB	X	4	-0.301	5.722	-12.397
ATOM	60	O3	0YB	X	4	-1.806	5.757	-10.890
ATOM	61	H3O	0YB	X	4	-2.758	5.883	-10.890
ATOM	62	C2	0YB	X	4	-1.607	7.404	-12.809
ATOM	63	H2	0YB	X	4	-2.669	7.613	-12.678
ATOM	64	N2	0YB	X	4	-0.838	8.345	-12.004
ATOM	65	H2N	0YB	X	4	0.111	8.040	-11.840
ATOM	66	C2N	0YB	X	4	-1.409	9.451	-11.568

ATOM	67	O2N	0YB	X	4	-2.499	9.773	-11.965
ATOM	68	CME	0YB	X	4	-0.611	10.211	-10.461
ATOM	69	H3M	0YB	X	4	-0.503	11.241	-10.800
ATOM	70	H2M	0YB	X	4	-1.179	10.196	-9.531
ATOM	71	H1M	0YB	X	4	0.375	9.746	-10.427
ATOM	72	C1	2MA	X	5	6.494	8.570	-18.855
ATOM	73	H1	2MA	X	5	7.510	8.301	-18.566
ATOM	74	C2	2MA	X	5	6.549	9.291	-20.231
ATOM	75	H2	2MA	X	5	7.321	10.059	-20.257
ATOM	76	C3	2MA	X	5	5.127	9.772	-20.482
ATOM	77	H3	2MA	X	5	4.782	10.461	-19.712
ATOM	78	C4	2MA	X	5	4.204	8.607	-20.543
ATOM	79	H4	2MA	X	5	4.390	7.973	-21.409
ATOM	80	C5	2MA	X	5	4.344	7.733	-19.286
ATOM	81	H5	2MA	X	5	3.849	8.236	-18.455
ATOM	82	C6	2MA	X	5	3.563	6.431	-19.573
ATOM	83	H62	2MA	X	5	2.690	6.611	-20.200
ATOM	84	H61	2MA	X	5	4.213	5.671	-20.005
ATOM	85	O6	2MA	X	5	3.108	6.056	-18.251
ATOM	86	H6O	2MA	X	5	3.729	6.252	-17.545
ATOM	87	O5	2MA	X	5	5.703	7.374	-19.057
ATOM	88	O4	2MA	X	5	2.875	9.134	-20.568
ATOM	89	H4O	2MA	X	5	2.264	8.553	-21.029
ATOM	90	O3	2MA	X	5	5.171	10.362	-21.727
ATOM	91	H3O	2MA	X	5	4.274	10.598	-21.973
ATOM	92	O2	2MA	X	5	6.956	8.287	-21.252
ATOM	93	C1	0YB	X	6	8.395	7.888	-21.269
ATOM	94	H1	0YB	X	6	8.813	7.969	-20.266
ATOM	95	O5	0YB	X	6	9.066	8.868	-22.114
ATOM	96	C5	0YB	X	6	10.513	8.552	-22.168
ATOM	97	H5	0YB	X	6	10.870	8.630	-21.141
ATOM	98	C6	0YB	X	6	11.259	9.692	-22.905
ATOM	99	H62	0YB	X	6	12.294	9.363	-23.013
ATOM	100	H61	0YB	X	6	11.231	10.702	-22.495
ATOM	101	O6	0YB	X	6	10.647	9.891	-24.142
ATOM	102	H6O	0YB	X	6	10.380	10.813	-24.164
ATOM	103	C4	0YB	X	6	10.760	7.195	-22.784
ATOM	104	H4	0YB	X	6	10.535	7.153	-23.850
ATOM	105	O4	0YB	X	6	12.239	6.976	-22.678
ATOM	106	H4O	0YB	X	6	12.608	7.256	-23.519
ATOM	107	C3	0YB	X	6	10.046	6.114	-22.056
ATOM	108	H3	0YB	X	6	10.530	5.972	-21.090
ATOM	109	O3	0YB	X	6	10.201	4.904	-22.796
ATOM	110	H3O	0YB	X	6	11.130	4.685	-22.903
ATOM	111	C2	0YB	X	6	8.548	6.494	-21.717
ATOM	112	H2	0YB	X	6	8.010	6.487	-22.665
ATOM	113	N2	0YB	X	6	8.008	5.490	-20.844
ATOM	114	H2N	0YB	X	6	8.563	5.224	-20.043
ATOM	115	C2N	0YB	X	6	6.829	5.059	-20.954
ATOM	116	O2N	0YB	X	6	6.100	5.531	-21.786
ATOM	117	CME	0YB	X	6	6.393	4.202	-19.851
ATOM	118	H3M	0YB	X	6	5.750	3.373	-20.148
ATOM	119	H2M	0YB	X	6	7.268	3.661	-19.489
ATOM	120	H1M	0YB	X	6	6.092	4.921	-19.090
